# [1,5]-Hydride Shift-Cyclization *versus* C(sp^2^)-H Functionalization in the Knoevenagel-Cyclization Domino Reactions of 1,4- and 1,5-Benzoxazepines

**DOI:** 10.3390/molecules25061265

**Published:** 2020-03-11

**Authors:** Dóra Szalóki Vargáné, László Tóth, Balázs Buglyó, Attila Kiss-Szikszai, Attila Mándi, Péter Mátyus, Sándor Antus, Yinghan Chen, Dehai Li, Lingxue Tao, Haiyan Zhang, Tibor Kurtán

**Affiliations:** 1 Department of Organic Chemistry, University of Debrecen, Debrecen, P. O. Box 400, Debrecen 4002, Hungary; szalokido@gmail.com (D.S.V.); tothlaszlochemist@gmail.com (L.T.); buglyo.balazs@science.unideb.hu (B.B.); kiss.attila@science.unideb.hu (A.K.-S.); mandi.attila@science.unideb.hu (A.M.); 2 Doctoral School of Chemistry, University of Debrecen, Egyetem tér 1, Debrecen 4032, Hungary; 3 Department of Organic Chemistry, Semmelweis University, Budapest 1094, Hungary; 4 Institute of Digital Health Sciences, Faculty of Health and Public Services, Semmelweis University, Ferenc tér 15, Budapest 1094, Hungary; peter.maty@gmail.com; 5 Key Laboratory of Marine Drugs, Chinese Ministry of Education, School of Medicine and Pharmacy, Ocean University of China, Qingdao 266003, China; qd_yinghan@163.com (Y.C.); dehaili@ouc.edu.cn (D.L.); 6 CAS Key Laboratory of Receptor Research, Shanghai Institute of Materia Medica, Chinese Academy of Sciences, 555 Zu Chong Zhi Road, Zhang Jiang Hi-Tech Park, Shanghai 201203, China; lingxuetao@simm.ac.cn (L.T.); hzhang@simm.ac.cn (H.Z.)

**Keywords:** domino Knoevenagel-[1,5]-hydride shift-cyclization, 1,4-benzoxazepine, 1,5-benzoxazepine, TDDFT-ECD calculation, acetylcholinesterase inhibitory activity

## Abstract

Domino cyclization reactions of *N*-aryl-1,4- and 1,5-benzoxazepine derivatives involving [1,5]-hydride shift or C(sp^2^)-H functionalization were investigated. Neuroprotective and acetylcholinesterase activities of the products were studied. Domino Knoevenagel-[1,5]-hydride shift-cyclization reaction of *N*-aryl-1,4-benzoxazepine derivatives with 1,3-dicarbonyl reagents having active methylene group afforded the 1,2,8,9-tetrahydro-7b*H*-quinolino [1,2-*d*][1,4]benzoxazepine scaffold with different substitution pattern. The C(sp^3^)-H activation step of the tertiary amine moiety occurred with complete regioselectivity and the 6-*endo* cyclization took place in a complete diastereoselective manner. In two cases, the enantiomers of the chiral condensed new 1,4-benzoxazepine systems were separated by chiral HPLC, HPLC-ECD spectra were recorded, and absolute configurations were determined by time-dependent density functional theory- electronic circular dichroism (TDDFT-ECD) calculations. In contrast, the analogue reaction of the regioisomeric *N*-aryl-1,5-benzoxazepine derivative did not follow the above mechanism but instead the Knoevenagel intermediate reacted in an S_E_Ar reaction [C(sp^2^)-H functionalization] resulting in a condensed acridane derivative. The AChE inhibitory assays of the new derivatives revealed that the acridane derivative had a 6.98 μM IC_50_ value.

## 1. Introduction

**Scheme 1:**
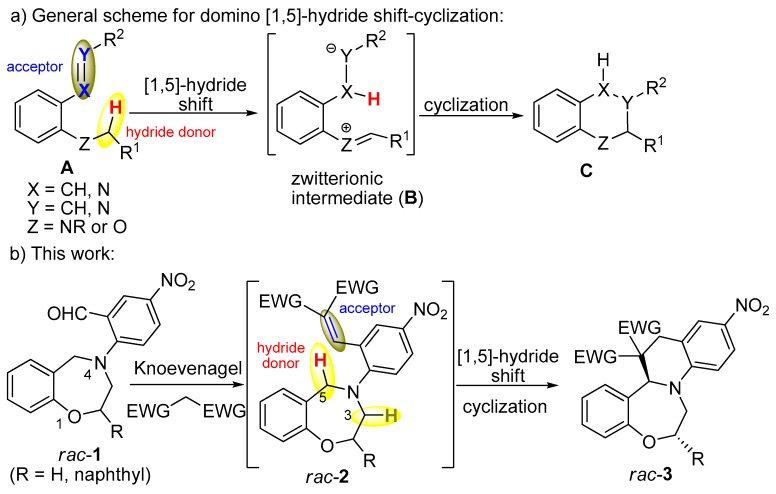
(**a**) General scheme for the C (sp^3^)-H activation-cyclization sequence induced by [1,5]-hydride shift of a tertiary amine or ether. (**b**) [1,5]-Hydride shift-cyclization sequence of *N*-aryl-1,4-benzoxazepines carried out in our present work.

C-H activation and functionalization of sp^3^- and sp^2^-hybridized carbons offer atom-economical and step-efficient ways for the preparation of polyfunctionalized condensed heterocycles, which have been intensely investigated recently [1,2,3,4]. The metal-free sp^3^ C-H functionalization via internal redox processes or hydride transfer has attracted much attention due to its unique features. Instead of using an external oxidant such as a high-valent transition metal [5,6], the sp^3^ C-H activation can be induced by an internal [1,5]-hydride shift between a *π* electron-poor double bond as acceptor and a C-H bond adjacent to an oxygen or nitrogen as a hydride donor (**A**→**B**, Scheme 1a) [7,8]. The resultant zwitterionic intermediate **B** can undergo further cyclization producing condensed heterocycles **C**.

In this work, the substrate (*rac*-**2**) able to undergo a [1,5]-hydride shift was obtained as a Knoevenagel intermediate in the reaction of *N*-aryl-1,4-benzoxazepine derivatives *rac-***1** with active methylene reagents. The 5-H benzylic hydrogen of *rac-***2**, adjacent to a tertiary amine nitrogen atom, acted as a hydride donor and a zwitterionic intermediate, formed in a [1,5]-hydride shift, cyclized affording the condensed tetracyclic 1,4-benzoxazepines *rac*-**3** with complete regio- and diastereoselectivity.

Condensed 1,4-benzoxazepines are common synthetic targets, since they are core structures in numerous biologically active compounds possessing antihelmentic [9], antitumor [10], anti-HIV [11], transient receptor potential ankyrin 1 (TRPA1) agonist [12], Rab geranylgeranyl transferase (RabGGTase) inhibitory [13], and antipsychotic [14] activities.

**Scheme 2:**
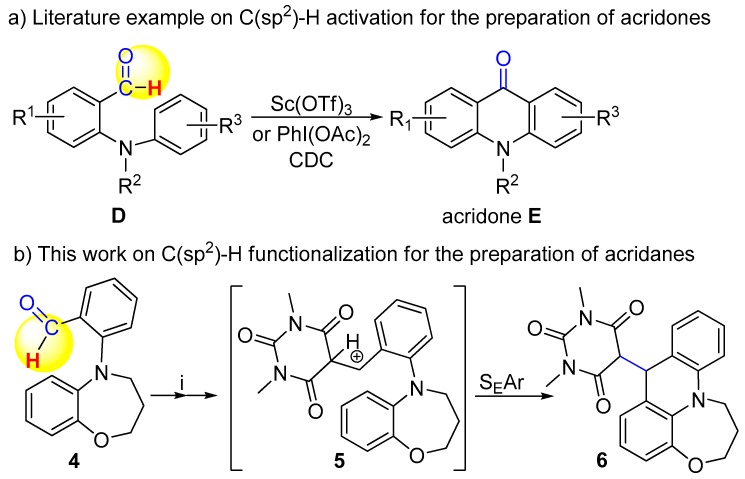
(**a**) C(sp^2^)-H activation and intramolecular cross-dehydrogenative coupling (CDC) reaction for the preparation of acridones. (**b**) This work on the C(sp^2^)-H functionalization of the *N*-aryl-1,5-benzoxazepine **4**; i: 1,3-dimethylbarbituric acid, MgSO_4_, CHCl_3_.

In reported examples, the C(sp^2^)-H activation of 2-(*N*-arylamino)benzaldehyde derivatives **D** was utilized to synthesize acridone derivatives **E** in an intramolecular cross-dehydrogenative coupling (CDC) reaction with PhI(OAc)_2_ or Sc(OTf)_3_ reagent (Scheme 2a) [15,16].

In this work, the *N*-aryl-1,5-benzoxazepine derivative **4** was converted to the carbocationic intermediate **5** with 1,3-dimethylbarbituric acid in a Knoevenagel reaction, which cyclized with the activated benzene ring to produce a condensed 9,10-dihydroacridine (acridane) derivative **6** in an S_E_Ar reaction. The different position of the nitrogen atom in the benzoxazepine scaffold induced a sequence different from those depicted in Scheme 1b for the regioisomeric *rac*-**1**; a C(sp^2^)-H functionalization took place instead of the [1,5]-hydride shift-cyclization, which was observed for *N*-aryl-1,4-benzoxazepines. The transformation of **4** to **6** involves the activation of the carbonyl carbon during the Knoevenagel reaction and a C(sp^2^)-H functionalization during the S_E_Ar reaction. 

Acridane derivatives have potent biological activity consisting of HIV reverse transcriptase inhibitory [17] and neuroleptic activity [18], and activation of K2P potassium channels [19]. Moreover, acridanes have been utilized as host materials in OLEDs [20], chemiluminescent sensors in immunoassays [21], as molecular motors [22] and NADH analogues in hydride transfer reactions [23].

Acetylchloninesterae (AChE) inhibitors are used as drugs for the symptomatic treatment of Alzheimer’s disease (AD), and some of them such as galantamine contain condensed *O,N*-heterocyclic skeleton [24]. AChE inhibitors prevent the action of cholinesterases (ChEs) and thus increase the levels of acetylcholine in the brain and improve the cholinergic functions in AD patients [25]. The inhibition of AChE is a potential therapeutic target for the treatment of AD but targeting ChEs alone is not sufficient and thus exploration of multi-target molecules focusing also on other proposed molecular mechanisms of AD such as *β*-amyloid cascade or oxidative stress is required. The multi-target approach was pursued in recently reported works by exploring synthetic heterocyclic derivatives, which exhibited both AChE and *β*-secretase inhibitory activity [26,27].

Oxidative stress, deriving from the accumulation of reactive oxygen species or an excess of free radicals, is involved in the progression of several neurodegenerative diseases, including the AD [28]. Recently, we reported the synthesis of condensed tetrahydro-1,4-benzoxazepines and an isochroman-2*H*-chromene conjugate, which showed neuroprotective activities against hydrogen peroxide (H_2_O_2_) and *β*-amyloid_25–35_ (A*β*_25–35_)-induced cellular injuries in human neuroblastoma SH-SY5Y cells [29,30]. In this work, acethylcholinesterase (AChE) inhibitory activity of new condensed 1,4- and 1,5-benzoxazepines was tested as well as neuroprotective activities against hydrogen peroxide (H_2_O_2_), *β*-amyloid_25–35_ fragment (A*β*_25–35_) and oxygen-glucose deprivation (OGD)-induced neurotoxicity in human neuroblastoma SH-SY5Y cells and a 1,5-benzoxazepine derivative was identified to have AChE inhibitory activity with 6.98 × 10^−6^ mol/L IC_50_ value.

## 2. Results and Discussion

### 2.1. Knoevenagel-[1,5]-Hydride Shift-Cyclization Domino Reaction

**Scheme 3:**
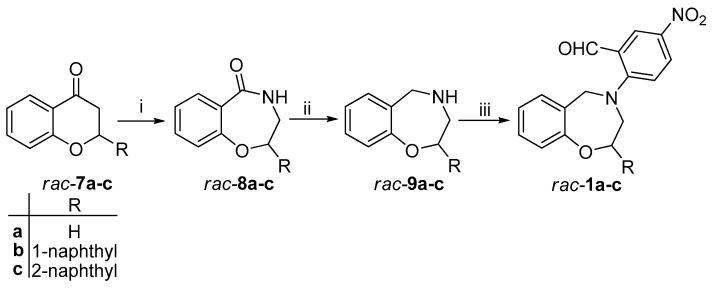
i: (1) NaN_3_/TFA or H_2_SO_4_ (2) H_2_O (*rac*-**8a**: 78%, *rac*-**8b**: 73%, *rac*-**8c**: 76%); ii: LAH/THF, reflux (*rac*-**9a**: 80%, *rac*-**9b**: 91%, *rac*-**9c**: 94%); iii: 2-fluoro-5-nitrobenzaldehyde or 2-chloro-5-nitrobenzaldehyde, K_2_CO_3_, toluene, reflux (*rac*-**1a**: 95%, *rac*-**1b**: 78%, *rac*-**1c**: 81%).

The synthesis of 4-aryl-1,4-benzoxazepine derivatives *rac-***1a**–**c**, the starting materials of the domino Knoevenagel-[1,5]-hydride shift-cyclization reaction, was achieved in a simple three-step sequence from the readily available chroman-4-one (*rac*-**7a**), 2-(1-naphthyl)-chroman-4-one (*rac*-**7b**) [31] and 2-(2-naphthyl)-chroman-4-one (*rac*-**7c**) [31] (Scheme 3).

**Scheme 4:**
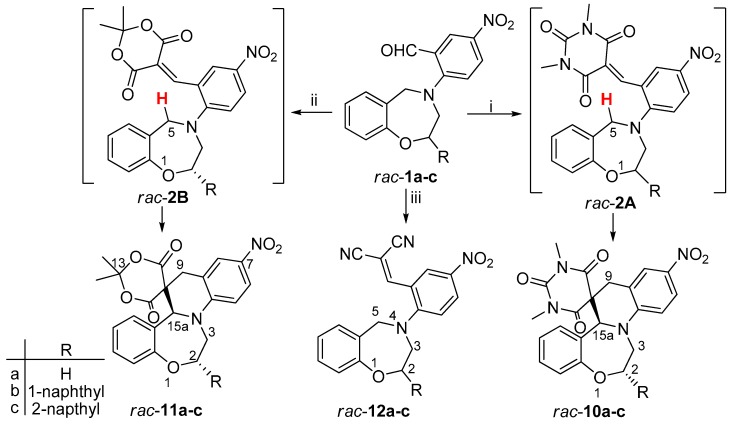
i: 1,3-dimetylbarbituric acid, MgSO_4_, CHCl_3_, reflux, 6 h (**10a**: 95%, **10b**: 36%, **10c**: 52%); ii: Meldrum’s acid, MgSO_4_, CHCl_3_, reflux, 6 h (**11a**: 87%, **11b**: 50%, **11c**: 98%); iii: malonitrile, MgSO_4_, CHCl_3_, reflux, 6 h (**12a**: 50%, **12b**: 53% **12c**: 61%).

In the first step, a regioselective Schmidt reaction of *rac*-**7a**–**c** was carried out with NaN_3_ affording the corresponding 3,4-dihydro-1,4-benzoxazepine-5(2*H*)-ones *rac*-**8a**–**c** (Scheme 3), which were reduced with LiAlH_4_ under reflux to provide 2,3,4,5-tetrahidro-1,4-benzoxepines (*rac*-**9a**–**c**). In the final step, the *N*-arylation of *rac*-**9a** was performed with 2-fluoro-5-nitrobenzaldehyde to result in *rac*-**1a** in 95% yield. For the *N*-arylation of *rac*-**9b** and *rac*-**9c**, the more economical but less reactive 2-chloro-5-nitrobenzaldehyde could be used instead. Furthermore, *rac*-**1a**–**c** were reacted with 1,3-dimetylbarbituric and Meldrum’s acids, which initiated a Knoevenagel-[1,5]-hydride shift-cyclization domino reaction (Scheme 4). The 1- and 2-naphthyl groups are known to adopt different orientations relatively to the condensed heterocyclic skeleton and they also exhibit different rotational energy barriers for their rotations, which may result in different bioactivities as demonstrated recently for our synthetic pterocarpans having antiproliferative activity [32].

The Knoevenagel intermediates **2A** and **2B** formed with 1,3-dimethylbarbituric acid and Meldrum’s acid, respectively, underwent a regioselective [1,5]-hydride shift with the participation of the 5-H and the resultant zwitterionic intermediate of type **B** (Scheme 1a) reacted further in a diastereoselective 6-*endo* cyclization affording the *rac*-*trans*-**10b**,**c**, *rac*-*trans*-**11b**,**c** and *rac*-**10a** and *rac*-**11a** (Scheme 4). The formation of the other regio- or diastereomeric products was not observed. The complete regioselectivity could be readily explained on the basis of the larger reactivity of the benzylic C-H bond compared to that of the 3-C-H, while the *trans*-diastereoselectivity was favored by the attack of the nucleophile from the face opposite to the orientation of the bulky naphthyl group linked at C-2. The relative configuration of the C-2 and C-15a chirality centers could not be determined on the basis of NOE correlations, since neither diastereomers would show NOE correlation between 2-H and 15a-H due to the large interatomic distance and orientation. In the *trans*-diastereomers, the tetrahydrooxazepine ring is expected to adopt a boat conformation with *trans-diaxial* orientation of the 2-H_ax_ and 3-H_ax_ protons giving rise to a large coupling ^3^*J*_2-H,3-H_ constant (~11 Hz) similarly to the 2-phenyl analogues [29], which could be observed in the ^1^H NMR spectra of **10b**,**c** and **11b**,**c**. In contrast, the tetrahydrooxazepine ring of the *cis* diastereomer would adopt a twist boat conformation with *gauche* arrangement of the 2-H and 3-Hs, which would produce small values for both ^3^*J*_2-H,3-H_ coupling constants.

With the malonitrile reagent, the reaction stopped at the stage of the Knoevenagel products **12a**–**c**. Stronger reaction conditions or microwave activation produced only partial cyclization or degradations.

**Figure 1:**
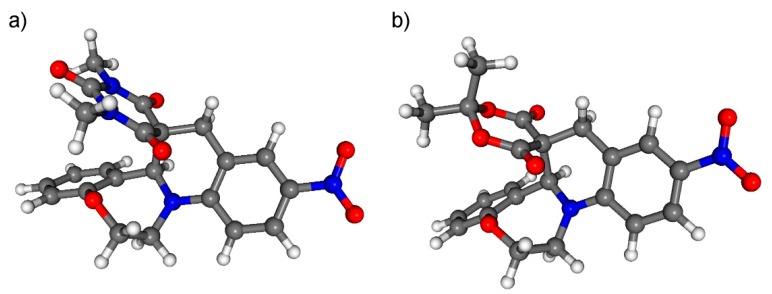
(**a**) Lowest-energy conformers of (*S*)-**10a** (99.1% population) and (**b**) (*S*)-**11a** (>99.9% population). Level of DFT optimization: CAM-B3LYP/TZVP PCM/*n*-octanol.

Since the **1a** starting material was achiral and its domino reaction introduced a new chirality center, the formation of **10a** and **11a** offered the possibility to test enantioselective organocatalytic reactions with non-covalent optically active organocatalysts. According to literature data, enantioselective organocatalytic versions of [1,5]-hydride shift-cyclization sequences are limited to substrates, in which the alkene double bond of the acceptor unit contains two alkoxycarbonyl substituents able to interact with the organocatalyst in stereoselective manner [33,34,35]. In order to determine the absolute configuration of the enantiomers of **10a** and **11a** and correlate it with the characteristic electronic circular dichroism (ECD) transitions, the enantiomers of **10a** and **11a** were separated by chiral HPLC using Chiralpack IC stationary phase and online HPLC-ECD spectra of the separated enantiomers were recorded. HPLC-ECD measurements aided with time-dependent density functional theory electronic circular dichroism (TDDFT-ECD) calculations represent an efficient method to study the enantiomers in scalemic or racemic mixtures of natural [36,37] or synthetic derivatives [38,39]. The solution TDDFT-ECD approach [40] was applied to determine the absolute configuration of the separated enantiomers by comparing the experimental HPLC-ECD spectra with the computed ones. The Merck Molecular Force Field (MMFF) conformational search of the arbitrarily chosen (*S*) enantiomers of **10a** and **11a** resulted in 5 and 2 conformers in a 21 kJ/mol energy window, respectively. These conformers were re-optimized at various density functional theory (DFT) levels (B3LYP/6-31G(d), B97D/TZVP PCM/*n*-octanol and CAM-B3LYP/TZVP PCM/*n*-octanol) yielding only one major conformer at all the applied levels (Figure 1).

**Figure 2:**
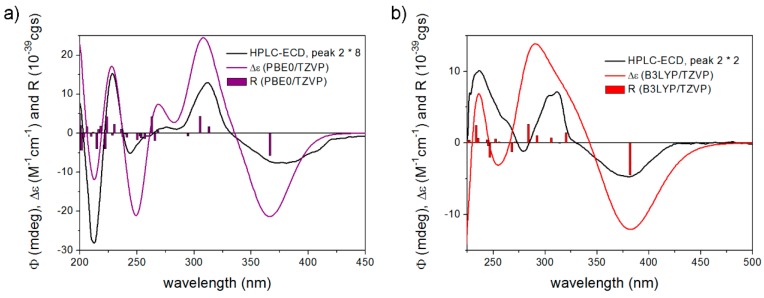
(**a**) Experimental HPLC-ECD spectra of the second-eluting enantiomer of **10a** (black line) compared with the PBE0/TZVP PCM/*n*-octanol spectrum of (*S*)-**10a** (purple line). (**b**) Experimental HPLC-ECD spectra of the second-eluting enantiomer of **11a** (black line) compared with the B3LYP/TZVP PCM/*n*-octanol spectrum of (*S*)-**11a** (red line). Level of DFT optimization: CAM-B3LYP/TZVP PCM/*n*-octanol. Bars represent the computed rotational strength values.

ECD spectra were then calculated at the B3LYP/TZVP, BH&HLYP/TZVP, CAM-B3LYP/TZVP and PBE0/TZVP levels with or without solvent model and all the applied combinations of levels gave moderate to good agreement with the experimental HPLC-ECD spectra of the second-eluting enantiomers allowing unambiguous determination of the absolute configuration of the second-eluting enantiomer as (*S*)-**10a** and (*S*)-**11a** (Figure 2). The (*S*)-**10a** and (*S*)-**11a** enantiomers are correlated with a broad negative Cotton effect (CE) above 350 nm in the HPLC-ECD spectra.

Although enantioselective [1,4]-hydride shift-cyclization cascade reaction of Knoevenagel intermediates prepared with dialkylmalonate were reported using optically active metal complexes [41] or organocatalysts [33,34,35], enantioselective transformations have not been published yet for Knoevenagel intermediates prepared with 1,3-dimethylbarbituric acid and Meldrum’s acid. Thus, we performed the **1a** → **10a** and **1a** → **11a** transformations in the presence of optically active TADDOL- and chinchona-type non-covalent organocatalysts to test the possibility of enantioselective reactions but unfortunately no notable enantiomeric excess (ee ≤ 7.3%) could be achieved with slow and small conversion of the starting material (see Supporting Information for details).

### 2.2. Knoevenagel- C(sp^2^)-H Functionalization Domino Reaction

**Scheme 5:**
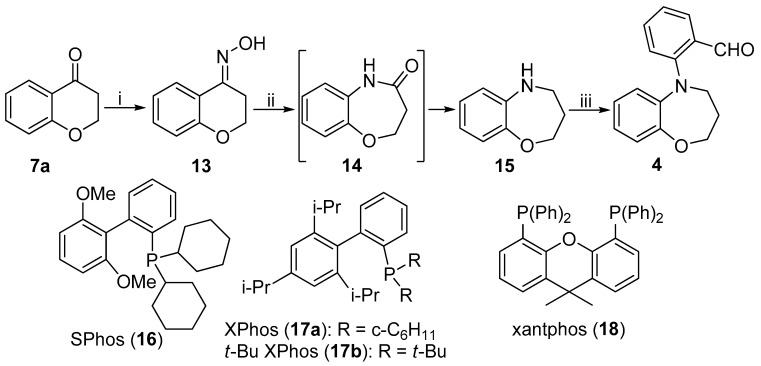
i: NH_2_OH∙HCl, pyridine (94%); ii: DIBAL-H, anhydrous CH_2_Cl_2_ (97% for two steps); iii: 2-bromo-benzaldehyde, Pd(OAc)_2_ (7.5 mol%), S-Phos (**16**, 10 mol%), Cs_2_CO_3,_ TBAB, anhydrous 1,4-dioxane, reflux (87%).

The reaction condition of the Knoevenagel-[1,5]-hydride shift-cyclization cascade was also tested on the analogous 1,5-benzoxazepine derivative **4**, in which only a less activated C-4 methylene group is available for the hydride shift. The *N*-aryl-1,5-benzoxazepine analogue **4**, starting material of the domino reaction, was synthesized in four steps from chromanone (Scheme 5). The classical Beckmann rearrangement of the (*E*)-ketoxime (**13**) afforded the 2,3-dihydro-1,5-benzoxazepin-4(5*H*)-one (**14**) [42], which without isolation, could be readily reduced to **15** with DIBAL-H [43]. ***N***-arylation of **15** with 2-fluoro-4-nitrobenzaldehyde could not be achieved in an S_N_Ar reaction with potassium or cesium carbonate in dry DMF at 140 °C due to the reduced nucleophilicity of the arylamine nitrogen. Thus, Buchwald-Hartwig *N*-arylation of **15** was carried out with 2-bromobenzaldehyde, Pd(OAc)_2_, a sterically hindered phosphine ligand and tetra*-n*-butylammonium bromide (TBAB) [44]. The reaction was optimized by testing different phosphine ligands [SPhos (**16**), XPhos (**17a**), *t*-butyl XPhos (**17b**), xantphos (**18**)] anhydrous solvents (1,4-dioxane, THF, toluene) and bases (Cs_2_CO_3_, K_2_CO_3_, KOtBu) and the best condition [2-bromo-benzaldehyde, Pd(OAc)_2_ (7.5 mol%), SPhos (**16**, 10 mol%), Cs_2_CO_3,_ TBAB, anhydrous 1,4-dioxane, reflux] afforded the *N*-aryl derivative **4** with 87% yield.

**Scheme 6:**
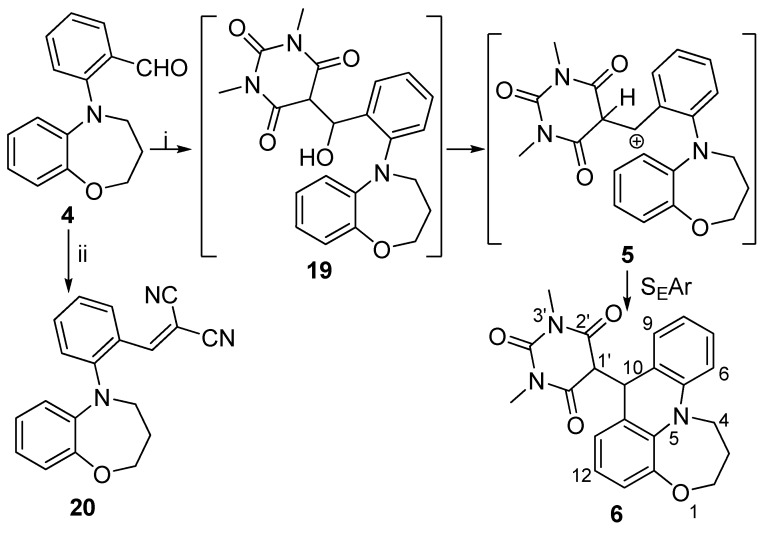
i. 1,3-dimetylbarbituric acid, MgSO_4_, CHCl_3_, rt (64%); ii. malonitrile, MeNH_2_∙HCl, CHCl_3_, rt (96%).

The *N*-aryl derivative **4** was reacted with 1,3-dimethylbarbituric acid and instead of the expected domino Knoevenagel-[1,5]-hydride shift-cyclization sequence, the **19** precursor of the Knoevenagel product formed a stabilized benzylic carbocation (**5**), which initiated an S_E_Ar reaction with the activated benzene ring affording the condensed acridane derivative **6** with 64% isolated yield (Scheme 6). According to the NMR data, all the methylene protons of the oxazepine moiety remained intact, while an aromatic C-H group was changed to a quaternary carbon. Two methine carbons appeared in the DEPT ^13^C NMR spectrum at 49.5 and 59.0 ppm corresponding to C-10 and C-1′, respectively, the protons of wich had a 4.4 Hz ^3^*J*_10-H,1′-H_ coupling constant. The condensed 2,3-dihydro-1*H*,8*H*-[1,4]oxazepino[2,3,4-*de*]acridine moiety has not been reported yet and direct conversion of 2-(*N*-arylamino)benzaldehyde derivatives to acridanes under mild conditions represents a new synthetic potential. The reaction of **4** with malonitrile stopped at the stage of the Knoevenagel product **20**, which could not be converted to a cyclized product even under stronger reaction condition (higher temperature, microwave activation). The reaction with Meldrum’s acid produced a complex reaction mixture, which has not been analyzed further.

### 2.3. Bioactivity Studies

The neuroprotective and AChE inhibitory activities of the new compounds were tested to compare neuroprotective activity with reported benzoxazepines and to identify candidates for multi-target approach. Neuroprotective activities were tested against hydrogen peroxide (H_2_O_2_), *β*-amyloid_23-35_ fragment (A*β*_23–35_) and oxygen-glucose deprivation (OGD)-induced neurotoxicity in human neuroblastoma SH-SY5Y cells [45,46]. The preliminary screenings showed that the tested compounds did not have remarkable neuroprotective activity at 10 µM concentration, which suggested that the lack of a C-2 aryl moiety or its extension to naphthyl groups results in the loss of activity [29]. Moderate AChE inhibitory activity was identified for the *N*-aryl-1,4-benzoxazepine **1a** at 4.0 × 10^−5^ M concentration (58.26% inhibition), while the acridane derivative **6** showed AChE inhibitory activity with 6.98 × 10^−6^ mol/L IC_50_ value.

## 3. Materials and Methods

Melting points were determined on a Kofler hot-stage apparatus (Wagner and Munz, Munich, Germany) and are uncorrected. The reactions were monitored by thin layer chromatography (TLC). TLC plates were visualized under a UV lamp and developed by phosphomolybdic acid solution. The NMR spectra were recorded on Bruker-AMX 400 (^1^H: 400 MHz; ^13^C: 100 MHz, Bruker, Karlsruhe, Germany; Billerica, MA, USA) and Bruker Aspect 3000 (^1^H: 360 MHz, ^13^C: 90 MHz, Bruker, Karlsruhe, Germany; Billerica, MA, USA) spectrometers using TMS and the solvent peak as internal standard. Chemical shifts were reported as *δ* in ppm and ^3^*J*_H,H_ coupling constants in Hz. Chiral HPLC separation of **10a** and **11a** were performed on a Jasco HPLC system with Chiralpak IC column (5 μm, 150 × 4.6 mm, hexan/propan-2-ol 1:1 eluent, 1 mLmin^−1^ flow rate, Daicel Chemical Industries Ltd., Tokyo, Japan) and HPLC-ECD spectra were recorded in stopped-flow mode on a JASCO J-810 electronic circular dichroism spectropolarimeter (JASCO Inc. Tokyo, Japan) equipped with a 10 mm HPLC flow cell. ECD ellipticity (*ϕ*) values were not corrected for concentration. For an HPLC-ECD spectrum, three consecutive scans were recorded and averaged with 2 nm bandwidth, 1 s response, and standard sensitivity. The HPLC-ECD spectrum of the eluent recorded in the same way was used as background. The concentration of the injected sample was set so that the HT value did not exceed 500 V in the HT channel down to 230 nm. IR spectra were recorded on a JASCO FT/IR-4100 spectrometer (JASCO Inc. Tokyo, Japan) and absorption bands are presented in cm^−1^. Electrospay Quadrupole Time-of-Flight HRMS measurements were performed with a MicroTOF-Q type QqTOF MS instrument equipped with an ESI source from Bruker (Bruker Daltoniks, Bremen, Germany).

### 3.1. Computational Section

Mixed torsional/low mode conformational searches were carried out by means of the Macromodel 9.7.211 software using Merck Molecular Force Field (MMFF) force field with implicit solvent model for chloroform applying a 21 kJ/mol energy window [47]. Geometry reoptimizations [B3LYP/6-31G(d) level in gas phase, B97D/TZVP and CAM-B3LYP/TZVP levels with PCM solvent model for *n*-octanol] and TDDFT-ECD calculations were performed with Gaussian 09 using various functionals (B3LYP, BH&HLYP, CAM-B3LYP, PBE0) and TZVP basis set for ECD calculations [48]. ECD spectra were generated as the sum of Gaussians with 1800**–**2400 cm^−1^ half-height width (corresponding to ca. 16–22 nm at 300 nm), using dipole-velocity computed rotational strength values [49]. Boltzmann distributions were estimated from the ZPVE corrected B3LYP energies in the gas phase calculations and from the uncorrected B97D and CAM-B3LYP energies in the PCM ones. The MOLEKEL software package was used for visualization of the results [50].

### 3.2. Bioassay on AChE Inhibitory Activity

The AChE assay was performed using the method of Ellman et al. with slight modification [51]. The cortex of Sprague–Dawley rat (400**–**500 g, obtained from the Animal Center of Shanghai Institute of Materia Medica) was homogenized in cold sodium phosphate buffer (75 mM, pH 7.4) as the AChE source. All the experimental procedures were approved by the Animal Care and Use Committee of Shanghai Institute of Materia Medica (#2012-02-ZHY-32 and 2015-03-ZHY-64). The assay solution consisted of 50 μL of 0.1 M phosphate buffer, 50 μL of 0.2% 5,5′-dithiobis(2-nitrobenzoic acid) (DTNB, Sigma), 109 μL of deionized water, 10 μL of rat cortex homogenate, and 30 μL of 2 mM acetylthiocholine iodide (Sigma) as the substrate of the AChE enzymatic reaction. An appropriate concentration of compound (0.1 mM to 5 mM; 1 μL) and the assay solution were incubated for 20 min at room temperature and the reaction was terminated with 50 μL of 3% (*w*/*v*) sodium dodecylsulfate (SDS). The production of the yellow anion of 5-thio-2-nitrobenzoic acid was measured with a microplate reader (DTX 880, Beckman Coulter, Brea, CA, USA) at 450 nm. The inhibition percentage caused by the presence of test compound was calculated, and the IC_50_ was defined as the concentration of the compound that reduced 50% of the enzymatic activity without inhibitor. The bioassay on neuroprotective activity was carried out according to the reference 25.

*3,4-Dihydro-1,4-benzoxazepin-5(2H)-one* (*rac*-**8a**): To a stirred solution of chroman-4-one (**7a**) (10.0 g, 67.49 mmol) in water and sulfuric acid 1:1 (100 mL), sodium azide (6.6 g, 101.5 mmol) was added in two equal portions at 0 °C and stirred at room temperature. The reaction was monitored by TLC,visualized under a UV lamp and developed by phosphomolybdic acid solution. After 5 h, the reaction mixture was neutralized with sodium hydroxide solution and the aqueous layer was extracted with dichloromethane (5 × 50 mL). The combined organic layers were washed with water (50 mL), dried over MgSO_4_, filtered and concentrated under reduced pressure. The crude product was purified by column chromatography on silica (EtOAc/hexane 2:5) to afford the *rac*-**8a** product as white solid [8.60 g, 52.71 mmol, 78%, mp 109–110 °C (lit. [52] mp 115–116 °C)]. ^1^H NMR (400 MHz, CDCl_3_): δ = 3.50 (dd, *J* = 9.6 Hz and 5.2 Hz, 3-H, 2 H), 4.39 (m, *J* = 9.6 Hz and 5.2 Hz, 2-H, 2 H), 7.03 (d, *J* = 8.0 Hz, 9-H, 1 H), 7.10 (m, 7-H, 1 H), 7.4 (m, 8-H, 1 H), 7.9 (dd, *J* = 8.0 Hz and 1.6 Hz, 6-H, 1 H), 8.2 (br s, 4-H, 1 H). ^13^C NMR (100 MHz, CDCl_3_): δ = 41.3 (C-3), 73.2 (C-2), 121.3 (C-9), 122.8 (C-7), 123.9 (C-5a), 131.6 (C-6), 133.3 (C-8), 155.3 (C-9a), 170.9 (C-5). IR (KBr): 1661, 3060, 3183, 3287 cm^−1^. HRMS-ESI (*m*/*z*): [M + Na]^+^ calc’d for C_9_H_9_NO_2_Na, 186.053; found: 186.053.

*2,3,4,5-Tetrahydro-1,4-benzoxazepine* (*rac*-**9a**): To a stirred solution of *rac*-**8a** (4.0 g, 24.51 mmol) in dry THF (60 mL), 2.0 M lithium aluminium hydride solution in THF (5 mL, 0.38 g, 10.02 mmol) was added dropwise and the mixture was refluxed for 3 h. After cooling to room temperature, ethyl-acetate (5 mL), methanol (5 mL) and water (50 mL) were added and the mixture was concentrated under reduced pressure. The residue was extracted with dichloromethane (3 × 50 mL). The combined organic layers were washed with water (20 mL), dried over MgSO_4_, filtered and concentrated under reduced pressure. The *rac*-**9a** product was isolated as yellow oil (2.92 g, 80%) [53]. ^1^H NMR (400 MHz, CDCl_3_): δ = 2.17 (s, 4-H, 1 H), 3.15 (m, 3-H, 2 H), 3.90 (s, 5-H, 2 H), 3.96 (m, 2-H, 2 H), 6.92–7.00 (m, 7-H and 9-H, 2 H), 7.08–7.20 (m, 6-H and 8-H, 2 H). ^13^C NMR (100 MHz, CDCl_3_): δ = 52.0 (C-5), 52.8 (C-3), 74.8 (C-2), 120.8 (C-9), 123.2 (C-7), 128.1 (C-6), 129.1 (C-8), 134.6 (C-5a), 159.8 (C-9a). IR (KBr): 766, 1226, 1488, 2858, 2932, 3312 cm^−1^. HRMS-ESI (*m*/*z*): [M + Na]^+^ calc’d for C_9_H_11_NONa, 172.074; found: 186.074.

*2-(2,3-Dihydro-1,4-benzoxazepin-4(5H)-yl)-5-nitrobenzaldehyde* (*rac*-**1a**): To the stirred solution of *rac*-**9a** (1.50 g, 9.19 mmol) in dry toluene (25 mL), anhydrous K_2_CO_3_ (1.85 g, 13.39 mmol) and 2-fluoro-5-nitrobenzaldehyde (2.04 g, 12.06 mmol) were added and the mixture was refluxed for 5 h. After cooling to room temperature, the K_2_CO_3_ was filtered off and toluene was removed under reduced pressure. The residue was purified by column chromatography on silica gel (ethyl-acetate/hexane 4:1) to give *rac*-**1a** as yellow solid (2.61 g, 95%, mp 101–102 °C). ^1^H NMR (400 MHz CDCl_3_): δ = 3.79 (m, *J* = 4.8 Hz, 3-H, 2 H), 4.35 (m, *J* = 4.8 Hz, 2-H, 1 H), 4.73 (s, 5-H, 2 H), 6.97 (d, *J* = 7.6 Hz, 9-H, 1 H), 7.01 (d, *J* = 9.2 Hz, 6′-H, 1 H), 7.09 (m, 7-H, 1 H), 7.23–7.27 (m, 6-H and 8-H, 2 H), 8.13 (dd, *J* = 9.2 Hz and 2.8 Hz, 5′-H, 1 H), 8.61 (d, *J* = 2.8 Hz, 3′-H, 1 H), 9.99 (s, 1H, -CHO). ^13^C-NMR (100 MHz, CDCl_3_): δ = 55.9 (C-3), 58.21 (C-5), 70.34 (C-2), 117.3 (C-9), 120.4 (C-6′), 123.4 (C-7), 124.0 (C-6a), 126.7 (C-2′), 129.0 (C-3′), 129.3* (C-8), 129.4* (C-6), 129.8* (C-5′), 139.7 (C-4′), 156.7 (C-9a), 158.7 (C-1′), 188.4 (-CHO). *exchangable signals. IR (KBr): 761, 1330, 1491, 1600, 1684 cm^−1^. HRMS-ESI (*m*/*z*): [M + Na]^+^ calc’d for C_16_H_14_N_2_O_4_Na, 321.085; found: 321.085.

*2-(2,3-Dihydro-1,4-benzoxazepin-4(5H)-yl)-5-nitrobenzylidene]propanedinitrile* (*rac*-**12a**): To a stirred solution of *rac*-**1a** (100 mg, 0.34 mmol) in chloroform (10 mL), anhydrous MgSO_4_ (150 mg, 1.24 mmol) and malononitrile (130 mg, 1.97 mmol) were added and the mixture was refluxed for 8 h. After cooling to room temperature, the MgSO_4_ was filtered off and chloroform was removed under reduced pressure. Water (10 mL) and dichloromethane (20 mL) were added, the two layers were separated and the aqueous phase was washed with dichloromethane (2 × 10 mL). The combined organic layers were washed with concentrated NaHCO_3_ solution, dried over MgSO_4_, filtered and concentrated under reduced pressure. The oily product was crystallized with ether to give *rac*-**12a** as brown solid (72 mg, 62%, mp 246–248 °C). ^1^H NMR (400 MHz CDCl_3_): δ = 3.75 (m, 3-H, 2 H), 4.34 (m, 2-H, 2 H), 4.45 (s, 5-H, 2 H), 7.07 (d, *J* = 8.0 Hz, 6-H, 1 H), 7.15–7.20 (m, 3′-H,7-H, 9-H, 3 H), 7.26-7.33 (m, 8-H, 1 H), 7.73 (s, 7′-H, 1 H), 8.2 (dd, *J* = 9.2 Hz and 2.4 Hz, 4′-H, 1 H), 8.84 (d, *J* = 2.4 Hz, 6′-H, 1 H). ^13^C NMR (100 MHz, CDCl_3_): δ = 56.8 (C-3), 59.1 (C-5), 70.6 (C-2), 84.1 (C-2”),111.8 (CN), 112.9 (CN), 118.8 (C-9), 120.8 (C-3′), 121.5 (C-1′), 124.1 (C-7),125.9 (C-6′), 127.6 (C-5a), 128.9 (C-4′), 129.4 (C-6), 129.8 (C-8), 140.9 (C-5′), 156.9 (C-7′), 158.2 (C-9a), 159.2 (C-2′). IR (KBr): 781, 1509, 1570, 2232, 2925 cm^−1^. HRMS-ESI (*m*/*z*): [M + Na]^+^ calc’d for C_19_H_14_N_4_O_3_Na, 369.096; found: 369.096.

*1,3-Dimethyl-11’-nitro-1’,2’-dihydro-2H,7b’H,9’H-spiro[pyrimidine-5,8’-quinolino[1,2-d][1,4]benzoxazepine]-2,4,6(1H,3H)-trione* (*rac*-**10a**): To a stirred solution of *rac*-**1a** (100 mg, 0.34 mmol) in chloroform (10 mL), anhydrous MgSO_4_ (150 mg, 1.25 mmol) and 1,3-dimetylbarbituric acid (80 mg, 0.51 mmol) were added and the mixture was refluxed for 6 h. After cooling to room temperature, the MgSO_4_ was filtered off and chloroform was removed under reduced pressure. Water (10 mL) and dichloromethane (20 mL) were added and two layers were separated. The aqueous phase was extracted with dichloromethane (2 × 10 mL). The combined organic layers were washed with concentrated NaHCO_3_ solution, dried over MgSO_4_, filtered and concentrated under reduced pressure. The oily product was crystallized with ether to give *rac*-**10a** as yellow solid (139 mg, 95%, mp 247–250 °C). ^1^H NMR (400 MHz DMSO-*d_6_*): δ = 2.76 (s, N-CH_3_, 3 H), 2.89 (s, N-CH_3_, 3 H), 3.20 (d, *J* = 17.2 Hz, 9-H_a_, 1 H), 3.48 (m, 3-H_a_, 1 H), 3.71–3.76 (overlapping m, 9-H_b_ and 3-H_b_, 2 H), 3.99–4.05 (m, 2-H_a_, 1 H), 4.22 (m, *J* = 3.6 Hz and 16.4 Hz, 2-H_b_, 1 H), 4.82 (s, 15-H_a_, 1 H), 6.98–7.02 (m, 16-H, 19-H, 2 H), 7.10 (d, *J* = 9.2 Hz, 5-H, 1 H), 7.17 (m, 17-H, 1 H), 7.39 (m, 18-H, 1 H), 7.97 (dd, *J* = 9.2 Hz and 2.8 Hz, 6-H, 1 H), 8.02 (bs, 8-H, 1 H). ^13^C NMR (100 MHz, DMSO-*d_6_*): δ = 28.0 (N-CH_3_), 28.1 (N-CH_3_), 33.6 (C-9), 42.8 (C-3), 51.3 (C-10), 69.8 (C-2), 70.5 (C-15a), 109.9 (C-5), 121.9 (C-15b), 122.4 (C-6), 123.1 (C-8), 124.4 (C-17, C-19), 127.2 (C-8a), 129.7 (C-18), 131.7 (C-16), 136.1 (C-7), 148.4 (C-13), 149.9 (C-19a), 152.9 (C-4a), 166.8 (C-15), 168.8 (C-11). IR (KBr): 1320, 1680, 2924 cm^−1^. HRMS-ESI (m/z): [M + Na]^+^ calc’d for C_22_H_20_N_4_O_6_Na, 459.128; found: 459.128.

*2,2-Dimethyl-11’-nitro-1’,2’-dihydro-7b’H,9’H-spiro[1,3-dioxane-5,8’-quinolino[1,2-d][1,4]benzoxazepine]-4,6-dione* (*rac*-**11a**): To the stirred solution of *rac*-**1a** (100 mg, 0.34 mmol) in chloroform (10 mL), anhydrous MgSO_4_ (150 mg, 1.25 mmol) and Meldrum’s acid (98 mg, 0.68 mmol) were added and the mixture was refluxed for 6 h. After cooling to room temperature, the MgSO_4_ was filtered off and chloroform was removed under reduced pressure. Water (10 mL) and dichloromethane (20 mL) were added and layers were separated. The aqueous phase was washed with dichloromethane (2 × 10 mL) and the combined organic layers were washed with concentrated NaHCO_3_ solution, dried over MgSO_4_, filtered and concentrated under reduced pressure. The oily product was crystallized with ether to give *rac*-**11a** as yellow solid (123 mg, 87%, mp 236–238 °C). ^1^H NMR (360 MHz, DMSO-*d_6_*): δ = 0.94 (s, CH_3,_ 3 H), 1.58 (s, CH_3_, 3 H), 3.57 (m, 3-H_a_, 9-H, 3 H), 3.71 (m, 2-H_a_, 1 H), 4.03 (m, 3-H_b_, 1 H), 4.20 (m, 2-H_b_, 1 H), 5.01 (s, 15a-H, 1 H), 7.07-7.14 (m, H-16, H-17, H-19, 3 H), 7.21 (m, 18-H, 1 H), 7.45 (m, 6-H, 1 H), 8.00 (m, 5-H, 8-H, 2 H). ^13^C NMR (90 MHz, CDCl_3_): δ = 26.4 (CH_3_), 29.5 (CH_3_), 34.6 (C-9), 43.2 (C-3), 49.3 (C-10), 68.9 (C-2), 69.7 (C-15a), 105.2 (C-13), 110.0 (C-19), 119.9 (C-8a), 122.8 (C-5), 123.7 (C-6), 124.6 (C-8), 124.7 (C-17), 130.4 (C-18), 131.5 (C-16), 135.9 (C-7), 148.3 (C-19a), 153.6 (C-4a), 163.6 (C-15), 167.7 (C-11). IR (KBr): 1262, 1313, 1736, 2924 cm^−1^.

*2-(Naphthalen-1-yl)-chroman-4-one* (*rac*-**7b**): To the stirred solution of the corresponding chalcone derivative (4.3 g, 16mmol) in EtOH, 130 mL of cc. NaOAc solution was added and refluxed for 5 h. After cooling it down to room temperature, EtOH was removed under reduced pressure. After addition of water, the aqueous layer was extracted with dichloromethane (5 × 50 mL). The combined organic layers were dried over MgSO_4_, filtered and concentrated under reduced pressure. The oily crude product was crystallized with cold diisopropyl-ether affording yellow crystals (3.14 g, 73%, mp 74–76 °C) [54]. ^1^H-NMR (360 MHz, CDCl_3_): δ = 3.05 (dd, *J* = 14.4 and 3.6 Hz, 1 H, 3-H_a_), 3.24 (dd, *J* = 14.4 and 10.8 Hz, 1 H, 3-H_b_), 6.19 (dd, *J* = 10.8 Hz and 3.6 Hz, 1 H, 2-H), 7.07-7.52 (m, 2 H, 6′-H, 7′-H), 7.53–7.57 (m, 4 H, 3′-H, 4′-H, 5′-H, 8′-H), 7.75 (d, 1 H, 8-H), 7.89 (m, 2 H, 2′-H, 6′-H), 8.00 (m, 2 H, 5-H, 7-H). ^13^C-NMR (90 MHz, CDCl_3_): δ = 43.9 (C-3), 76.8 (C-2), 118.2 (C-8), 121.1 (C-4a), 121.7 (C-6), 122.8 (C-8′), 123.8 (C-3′), 125.3 (C-6′), 125.9 (C-2′), 126.6 (C-7′), 127.1 (C-5), 129.1 (C-4′), 129.3 (C-5′), 130.1 (C-8a’), 133.8 (C-4a’), 134.1 (C-1), 136.2 (C-7), 161.7 (C-8a), 192.2 (C-4). IR (KBr): 1222, 1302, 1606, 1684, 3050 cm^−1^.

*2-(Naphthalen-2-yl)-chroman-4-one* (*rac*-**7c**): To a stirred solution of the chalcone derivative (4.3 g, 16 mmol) in EtOH, 130 mL of cc. NaOAc solution was added and refluxed for 5 h. After cooling it down to room temperature, EtOH was removed under reduced pressure. The residue was taken in water and extracted with dichloromethane (5 × 50 mL). The combined organic layers were dried over MgSO_4_, filtered and concentrated under reduced pressure. The oily crude product was crystallized with cold diisopropyl-ether affording yellow crystals (3.27 g, 76%, mp 110–112 °C) [54]. ^1^H-NMR (360 MHz, CDCl_3_): δ = 2.90 (dd, *J* = 16.5 Hz and 2.5 Hz, 1 H, 3-H_a_) 3.09 (dd, *J* = 16.5 Hz and 13.0 Hz, 1 H, 3-H_b_). 5.56 (dd, *J*=13.0 Hz and 2.5 Hz, 1 H, 2-H). 7.01-7.07 (m, 2 H, 6′-H, 7′-H). 7.47–7.55 (m, 4 H, 3′-H, 4′-H, 5′-H, 8′-H). 7.82–7.92 (m, 5 H, 5-H, 1′-H, 5-H, 6-H, 7-H), 7.93 (d, *J* = 7.6 Hz, 1 H, 8-H). ^13^C-NMR (90 MHz, CDCl_3_): δ = 44.5 (C-3), 79.6 (C-2), 118.1 (C-8), 120.9 (C-4a), 121.6 (C-6), 123.6 (C-3′), 125.3 (C-4′), 126.4 (C-6′, C-7′), 127.0 (C-1′), 127.7 (C-5′), 128.1 (C-5), 128.7 (C-8′), 133.1 (C-8a’), 133.3 (C-4a’), 135.9 (C-2′), 136.2 (C-7), 161.4 (C-8a), 191.8 (C-4). IR (KBr): 1063, 1223, 1688, 3025 cm^−1^.

*2-(Naphthalen-1-yl)-3,4-dihydrobenzo[f][1,4]oxazepin-5(2H)-one* (*rac*-**8b**): To the stirred soluton of *rac*-**7b** (3.0 g, 11 mmol) in acetic acid (30 mL), sodium azide (1.1 g, 17 mmol) was added in two parts at 0 °C, then 3.0 mL of cc. H_2_SO_4_ was added dropwise, and the reaction mixture was stirred at room temperature for 5 h. The reaction mixture was neutralized with sodium hydroxide solution and extracted with dichloromethane (5 × 50 mL). The combined organic layers were dried over MgSO_4_, filtered and concentrated under reduced pressure. The crude product was purified by column chromatography on silica (hexane/EtOAc 4:1) to give the product as white powder (3.14 g, 73%, mp 74–76 °C). ^1^H-NMR (400 MHz, CDCl_3_): δ = 3.57–3.62 (m, 1 H, 3-H_a_) 3.65–3.71 (m, 1 H, 3-H_b_), 5.58 (m, 1 H, 2-H), 7.10 (d, *J* = 8.0 Hz 1 H, 9-H), 7.21 (m, 1 H, 7-H), 7.46-7.49 (m, 4 H, 2-H’, 3-H’, 6-H, 8-H), 7.62 (s, 1 H, 4-H), 7.81–7.85 (m, 5 H, 4′-H, 5′-H, 6′-H, 7′-H, 8′-H), ^13^C-NMR (100 MHz, CDCl_3_): δ = 46.2 (C-3), 85.9 (C-2), 122.5 (C-9), 123.8 (C-7), 124.0 (C-8′), 125.5 (C-2′), 125.9 (C-5a), 126.4 (C-6′), 126.5 (C-3′), 127.7 (C-7′), 128.2 (C-4′), 128.5 (C-5′), 131.0 (C-6), 133.1 (C-8a’), 133.3 (C-4a’), 133.4 (C-8), 154.6 (C-9a), 171.2 (C-5), IR (KBr): 1024, 1217, 1462, 1604, 1655, 3062, 3212, 3283 cm^−1^. HRMS-ESI (*m*/*z*): [M + Na]^+^ calc’d for C_19_H_19_NO_2_Na, 312.100; found: 312.099.

*2-(Naphthalen-2-yl)-3,4-dihydrobenzo[f][1,4]oxazepin-5(2H)-one* (*rac*-**8c**): To the stirred solution of *rac*-**7c** (3.0 g, 11 mmol) in acetic acid (30 mL), sodium azide (1.1 g, 17 mmol) was added in two parts at 0 °C, then 3.0 mL of cc. H_2_SO_4_ was added dropwise and stirred at room temperature for 5 h. The reaction mixture was neutralized with sodium hydroxide solution and the aqueous mixture was extracted with dichloromethane (5 × 50 mL). The combined organic layers were dried over MgSO_4_, filtered and concentrated under reduced pressure. The crude product was purified by column chromatography on silica (hexane/EtOAc 4:1) to give the product as white powder (3.27 g, 76%, mp 110–112 °C). ^1^H-NMR (400 MHz, CDCl_3_) δ = 3.55–3.69 (m, 2 H, 3-H), 5.57 (br s, 1H, 2-H), 7.09 (d, *J* = 8.0 Hz, 1 H, 9-H), 7.18 (m, 1 H, 7-H), 7.47 (m, 4 H, 2′-H, 3′-H, 6-H, 8-H), 7.82-7.84 (m, 5 H 4′-H, 5′-H, 6′-H, 7′-H, 8′-H), ^13^C-NMR (100 MHz, CDCl_3_): δ = 46.2 (C-3), 85.9 (C-2), 122.5 (C-9), 123.8 (C-1′), 124.0 (C-9), 125.5 (C-7), 125.9 (C-5a), 126.4 (C-6′), 126.5 (C-7′), 127.7 (C-5′), 128.2 (C-4′), 128.5 (C-8′), 131.0 (C-6), 133.1 (C-8a’), 133.3 (C-4a’), 133.4 (C-8), 136.4 (C-2′), 154.6 (C-9a), 171.2 (C-5). IR (KBr): 1064, 1223, 1299, 1460, 1696, 2958 cm^−1^. HRMS-ESI (*m*/*z*): [M + Na]^+^ calc’d for C_19_H_19_NO_2_Na, 312.100; found: 312.099.

*2-(Naphthalen-1-yl)-2,3,4,5-tetrahydrobenzo[f][1,4]oxazepine* (*rac*-**9b**): To the stirred solution of *rac*-**8b** (2.0 g, 6.9 mmol) in dry THF (10 mL), lithium aluminum hydride solution in hexane (2.0 M) was added dropwise (10 mL, 0.72 g, 20.04 mmol) and the mixture was refluxed for 1 h. After cooling to room temperature, 20 mL 4 M NaOH solution was added and the mixture was concentrated under reduced pressure. The residue was extracted with dichloromethane (3 × 50 mL). The combined organic layers were dried over MgSO_4_, filtered and concentrated under reduced pressure. The product was isolated as brown oil (1.82 g, 91%). ^1^H-NMR (400 MHz, CDCl_3_): δ = 1.86 (br s, 1 H, 4-H) 3.24–3.45 (dd, *J* = 12.0 Hz and 8.0 Hz, 1 H, 3-Ha), 3.44 (d, *J* = 12.0 Hz, 1 H, 3-H_a_), 3.97 (d, *J* = 16.0 Hz, 1 H, 5-H_a_), 4.15 (d, *J* = 12.0 Hz, 1 H, 5-H_b_), 4.80 (d, *J* = 8.0 Hz, 1 H, 2-H), 7.01–7.09 (m, 2 H, 7-H, 9-H), 7.16–7.21 (m, 2 H, 5′-H, 6-H), 7.46–7.52 (m, 3 H, 2′-H, 3′-H, 8-H), 7.82–7.88 (m, 4 H, 4′-H, 6′-H, 7′-H, 8′-H), ^13^C-NMR (100 MHz, CDCl_3_): δ = 52.6 (C-5), 59.0 (C-3), 86.5 (C-2), 121.7 (C-9), 123.8 (C-7), 124.1 (C-8′), 124.7 (C-6′), 126.0 (C-2′), 126.3 (C-3′), 127.8 (C-7), 128.0 (C-4), 128.2 (C-8), 128.5 (C-6), 129.1 (C-5′), 132.9 (C-5a), 133.3 (C-8a’), 135.7 (C-4a’), 137.9 (C-1′), 159.2 (C-9a), IR (KBr): 1047, 1232, 1483, 1599, 2899, 3241, 3315 cm^−1^.

*2-(Naphthalen-2-yl)-2,3,4,5-tetrahydrobenzo[f][1,4]oxazepine* (*rac*-**9c**): To the stirred solution of *rac*-**8c** (2.0 g, 6.9 mmol) in dry THF (10 mL), lithium aluminum hydride solution in hexane (2.0 M) was added dropwise (10 mL, 0.72 g, 20.04 mmol) and the mixture was refluxed for 1 h. After cooling to room temperature, 20 mL 4 M NaOH solution was added and the mixture was concentrated under reduced pressure. The residue was extracted with dichloromethane (3 × 50 mL). The combined organic layers were dried over MgSO_4_, filtered and concentrated under reduced pressure. The product was isolated as brown oil (1.88g, 94%). ^1^H-NMR (400 MHz, CDCl_3_): δ = 1.94 (br s, 1 H, 4-H). 3.25–3.47 (m, 1 H, 3-H_a_), 3.45 (d, *J* = 16.0 Hz, 1 H, 3-H_b_), 3.98 (d, *J* = 16.0 Hz, 1 H, 5-H_a_), 4.16 (d, *J* = 16.0 Hz 1 H, 5-H_b_), 4.81 (d, *J* = 8.0 Hz 1 H, 2-H), 7.02-7.10 (m, 2 H, 6-H, 8-H), 7.17–7.22 (m, 2 H, 7-H, 9-H), 7.45–7.52 (m, 3 H, 3′-H, 6′-H, 8′-H), 7.82-7.89 (m, 4 H, 1′-H, 4′-H, 5′-H, 7′-H), ^13^C-NMR (100 MHz, CDCl_3_): δ = 52.6 (C-5), 59.0 (C-3), 86.5 (C-2), 121.7 (C-9), 123.8 (C-7), 124.1 (C-3′), 124.7 (C-1′), 126.0 (C-6′), 126.3 (C-7′), 127.7 (C-4′), 128.1 (C-8), 128.2 (C-5′), 128.5 (C-8′), 129.1 (C-6), 132.9 (C-5a), 133.3 (C-8a’), 135.7 (C-4a’), 137.9 (C-2′), 159.2 (C-9a), IR (KBr): 1078, 1241, 1470, 2963, 3240, 3350 cm^−1^.

*2-(Naphthalen-1-yl)-4-(4-nitrophenyl)-2,3,4,5-tetrahydrobenzo[f][1,4]oxazepine* (*rac*-**1b**): To the stirred solution of *rac*-**9b** (1.30 g, 4.70 mmol) in dry toluene (20 mL), 2-chloro-5-nitrobenzaldehyde (1.75 g 9.4 mmol) and anhydrous K_2_CO_3_ (3.2 g 2.3 mmol) were added and the mixture was refluxed for 5 h. After cooling to room temperature, the K_2_CO_3_ was filtered off and toluene was removed under reduced pressure. The residue was purified by column chromatography on silica gel (hexane/EtOAc 4:1) affording the product as yellow solid (1.01 g, 78%, mp 179–181 °C). ^1^H-NMR (400 MHz, CDCl_3_): δ = 3.71 (dd, *J* = 14.0 Hz and 9.0 Hz, 1 H, 3-H_a_) 4.12 (dd, *J* = 14.0 Hz and 4.0 Hz, 1 H, 3-H_b_), 4.74 (d, *J* = 16.0 Hz,1 H, 5-H_a_), 4.95 (d, *J* = 16.0 Hz, 1 H, 5-H_b_), 5.45 (dd, *J* = 9.0 Hz and 4.0 Hz, 1 H, 2-H), 7.05 (m, 2 H, 7-H, 9-H), 7.15–7.17 (m, 1 H, 6”-H), 7.30-7.32 (m, 2 H, 6-H, 8-H), 7.49–7.52 (m, 3 H, 2′-H, 3′-H, 4′-H), 7.84–7.89 (m, 4 H, 5′-H, 6′-H, 7′-H, 8′-H), 8.13–8.16 (dd, *J* = 8.0 Hz and 2. 8 Hz, 1 H, 5”-H), 8.60 (d, *J* = 2.8 Hz, 1 H, 3”-H), 9.95 (s, 1 H, CHO), ^13^C-NMR (100 MHz, CDCl_3_): δ = 56.3 (C-5), 64.5 (C-3), 82.9 (C-2), 117.8 (C-9), 121.1 (C-6”), 123.6 (C-7), 124.0 (C-8′), 124.4 (C-5a), 125.1 (C-6′), 126.5 (C-2′), 126.6 (C-3′), 127.8 (C-7′), 128.1 (C-8), 128.7 (C-4′), 129.2 (C-3”), 129.3 (C-5′), 129.5 (C-6), 129.9 (C-5”), 133.1 (C-8a’), 133.2 (C-4a’), 135.5 (C-4”), 140.0 (C-1′), 156.8 (C-9a), 158.3 (C-1”), 188.4 (CHO). IR (KBr): 1489, 1600, 1684, 2750, 2917, 3053 cm^−1^. HRMS-ESI (*m*/*z*): [M + Na]^+^ calc’d for C_26_H_20_N_2_O_4_Na, 447.132; found: 447.132.

*2-(Naphthalen-2-yl)-4-(4-nitrophenyl)-2,3,4,5-tetrahydrobenzo[f][1,4]oxazepine* (*rac*-**1c**): To the stirred solution of *rac*-**9c** (1.30 g, 4.70 mmol) in dry toluene (20 mL), 2-chloro-5-nitrobenzaldehyde (1.75 g, 9.4 mmol) and anhydrous K_2_CO_3_ (3.2 g, 2.3 mmol) were added and the mixture was refluxed for 5 h. After cooling to room temperature, the K_2_CO_3_ was filtered off and toluene was removed under reduced pressure. The residue was purified by column chromatography on silica gel (hexan/EtOAc 4:1) to give the product as yellow solid (1.05 g, 81%, mp 124–126 °C). ^1^H-NMR (400 MHz, CDCl_3_): δ = 3.70 (m, 1 H, 3-H_a_), 4.12 (d, *J* = 12.0 Hz, 1 H, 3-H_b_), 4.73 (d, *J* = 16.0 Hz, 1H, 5-H_a_), 4.95 (d, *J* = 16.0 Hz, 1 H, 5-H_b_), 5.45 (d, *J* = 12.0 Hz, 1 H, 2-H), 7.02–7.16 (m, 3 H, 5′-H, 6”-H, 8′-H), 7.25–7.32 (m, 2 H, 7-H, 9-H), 7.79–7.51 (m, 3 H, 6′-H, 7′-H, 8′-H), 7.83–7.89 (m, 4 H, 1′-H, 3′-H, 4′-H, 6′-H), 8.13 (dd, *J* = 8.0 Hz, 1 H, 5”-H), 8.59 (d, *J* = 2.8 Hz, 1H, C-3”), 9.94 (s, 1 H, CHO), ^13^C-NMR (100 MHz, CDCl_3_): δ = 56.3 (C-5), 64.5 (C-3), 82.9 (C-2), 117.7 (C-9), 121.1 (C-6”), 123.6 (C-7), 123.9 (C-3′), 124.4 (C-5a), 125.1 (C-1′), 126.4 (C-6′), 126.5 (C-6′), 126.6 (C-7′), 127.7 (C-2′), 127.8 (C-4′), 127.9 (C-8), 128.6 (C-3”), 129.1 (C-5′), 129.2 (C-8′), 129.4 (C-6), 129.8 (C-5”), 133.1 (C-8a’), 133.2 (C-4a’), 135.5 (C-4”), 139.9 (C-2′’), 156.8 (C-9a), 158.3 (C-1”), 188.4 (CHO), IR (KBr): 1238, 1326, 1503, 1598, 1680, 2918, 3058 cm^−1^. HRMS-ESI (*m*/*z*): [M + Na]^+^ calc’d for C_26_H_20_N_2_O_4_Na, 447.132; found: 447.132.

*1,3-Dimethyl-2’-(naphthalen-1-yl)-11’-nitro-1’,2’-dihydro-2H,7b’H,9’H-spiro[pyrimidine-5,8’-quinolino[1,2-d][1,4]benzoxazepine]-2,4,6(1H,3H)-trione* (*rac*-**10b**): To the stirred solution of *rac*-**1b** (100 mg, 0.23 mmol) in chloroform (5 mL), anhydrous MgSO_4_ (150 mg, 1.25 mmol) and barbituric acid (38 mg, 0.29 mmol) were added and the mixture was refluxed for 24 h. After cooling to room temperature, the MgSO_4_ was filtered off and chloroform was removed under reduced pressure. The residue was purified by column chromatography on silica gel (hexane/EtOAc 1:1) affording the product as yellow solid (36 mg, 36%, mp 289–291 °C) ^1^H-NMR (400 MHz, DMF-*d*_7_): δ = 2.71 (s, 3 H, CH_3_), 2.98 (s, 3 H, CH_3_), 3.31 (d, *J* = 16.0 Hz, 1 H, 9-Ha), 3.83 (d, *J* = 16.0 Hz, 1H, 9-Hb), 4.10 (m, 1 H, 3-Ha), 4.61 (d, *J* = 16.0 Hz, 1 H, 3-Hb), 4.88 (s, 1 H, 15a-H), 5.79 (m, 1 H, 2-H), 6.09 (m, 1 H, 5-H), 7.00–7.04 (m, 5 H, Ph-H), 7.26–7.33 (m, 3 H, Ph-H), 7.48–7.61 (m, 2 H, Ph-H), 7.85–7.93 (m, 2 H, Ph-H), 8.03 (s, 1H, 8′-H), 8.13 (d, *J* = 8.0 Hz, 1 H, 6-H). ^13^C-NMR (100 MHz, DMF-*d*_7_): δ = 29.0 (CH_3_) 29.1 (CH_3_), 46.7 (C-9), 53.1 (C-15a), 72.2 (C-3), 76.6 (C-10), 111.6 (C-2), 105.5 (C-13), 123.7 (C-19), 124.2 (C-5), 124.6 (C-17), 124.7 (C-15b), 124.8 (C-6) 125.7 (C-8), 125.8 (C-8′), 124.8 (C-2′), 127.1 (C-6′), 127.8 (C-3′), 128.8 (C-7′), 129.9 (C-18), 130.4 (C-4′), 131.2 (C-5′),132.3 (C-16) 132.7 (C-8a’), 133.7 (C-4a’), 135.1 (C-7), 138.3 (C-1′), 149.9 (C-13), 151.4 (C-19a), 152.4 (C-4a), 168.6 (C-15), 170.3 (C-11). IR (KBr): 1322, 1601, 1683, 1698, 2853, 2923 cm^−1^. HRMS-ESI (*m*/*z*): [M + Na]^+^ calc’d for C_32_H_26_N_4_O_6_Na, 585.175; found: 585.175.

*1,3-Dimethyl-2’-(naphthalen-2-yl)-11’-nitro-1’,2’-dihydro-2H,7b’H,9’H-spiro[pyrimidine-5,8’-quinolino[1,2-d][1,4]benzoxazepine]-2,4,6(1H,3H)-trione* (*rac*-**10c**): To the stirred solution of *rac*-**1c** (100 mg, 0.23 mmol) in chloroform (5 mL), anhydrous MgSO_4_ (150 mg, 1.25 mmol) and 1,3-dimethylbarbituric acid (38 mg, 0.29 mmol) were added and the mixture was refluxed for 24 h. After cooling to room temperature, the MgSO_4_ was filtered off and the chloroform was removed under reduced pressure. The residue was crystallized with cold diisopropyl-ether affording the product as yellow solid (52 mg, 52%, mp 312–314 °C) The sample could not be dissolved in NMR solvents. IR (KBr): 1320, 1507, 1680, 1746, 2953, 3057 cm^−1^. HRMS-ESI (*m*/*z*): [M + Na]^+^ calc’d for C_32_H_26_N_4_O_6_Na, 585.175; found: 585.175.

*2,2-Dimethyl-2’-(naphthalen-1-yl)-11’-nitro-1’,2’-dihydro-7b’H,9’H-spiro[1,3-dioxane-5,8’-quinolino[1,2-d][1,4]benzoxazepine]-4,6-dione* (*rac*-**11b**): To the stirred solution of *rac*-**1b** (100 mg, 0.23 mmol) in chloroform (5 mL), anhydrous MgSO_4_ (150 mg, 1.25 mmol) and Meldrum’s acid (43 mg, 0.29 mmol) were added and the mixture was refluxed for 24 h. After cooling to room temperature, the MgSO_4_ was filtered off and the chloroform was removed under reduced pressure. The residue was purified by column chromatography on silica gel (hexane/EtOAc 2:1) affording the product as yellow solid (50 mg, 50%, mp 160–162 °C). ^1^H-NMR (400 MHz, CDCl_3_): δ = 1.05 (s, 3 H, CH_3_), 1.65 (s, 3 H, CH_3_), 3.24 (d, *J* = 16.0 Hz, 1 H, 9-H_a_), 3.90 (d, *J* = 16.0 Hz, 1 H, 9-H_b_), 4.19 (m, 1 H, 3-H_a_), 4.33 (m, 1 H, 3-H_b_), 5.08 (s, 1 H, 15a-H), 6.08 (d, *J* = 8.0 Hz, 1 H, 2-H), 6.55 (d, *J* = 8.0 Hz, 1 H, 5-H), 6.88(d, *J* = 8.0 Hz, 1 H, 17-H), 7.02 (m, 1 H, Ar-H), 7.21–7.34 (m, 4 H, Ar-H), 7.57–7.62 (m, 2 H, Ar-H), 7.89 (m, 1 H, 16-H), 8.10 (m, 2 H, 6-H, 8-H), 8.31 (m, 1 H, 18-H). ^13^C-NMR (100 MHz, CDCl_3_): δ = 26.9 (CH_3_) 30.4 (CH_3_), 35.9 (C-9), 46.5 (C-3), 49.9 (C-10), 70.6 (C-15a), 74.8 (C-2), 105.5 (C-13), 109.9 (C-19), 119.5 (C-5), 123.1 (C-17), 123.6 (C-6), 124.3 (C-8), 124.4 (C-8′), 124.8 (C-2′), 125.1 (C-8a), 125.5 (C-6′), 126.1 (C-3′), 126.9 (C-15b), 126.9 (C-7′), 128.7 (C-18), 129.5 (C-4′), 130.8 (C-16), 131.3 (C-5′), 131.5 (C-8a’), 132.5 (C-4a’), 133.8 (C-7), 137.9 (C-1′), 147.8 (C-19a), 152.2 (C-4a), 164.4 (C-15), 168.2 (C-11). IR (KBr): 1195, 1509, 1603, 1737, 1775, 2930, 3056 cm^−1^. HRMS-ESI (*m*/*z*): [M + Na]^+^ calc’d for C_32_H_20_N_2_O_7_Na, 573.164; found: 573.163.

*2’,2’-Dimethyl-2-(naphtalene-2-yl)11-nitro-1,2,7b,9-tetrahydrospiro[benzo[6,7][1,4]oxazepine[4,5-a]quinoline-8,5’-[1,3]dioxane]-4’,6’-dione* (*rac*-**11c**): To the stirred solution of *rac*-**1c** (100 mg, 0.23 mmol) in chloroform (5 mL), anhydrous MgSO_4_ (150 mg, 1.25 mmol) and Meldrum’s acid (43 mg, 0.29 mmol) were added and the mixture was refluxed for 24 h. After cooling to room temperature, the MgSO_4_ was filtered off and the chloroform was removed under reduced pressure. The residue was purified by column chromatography on silica gel (hexane/EtOAc 2:1) affording the product as yellow solid (98 mg, 98%, mp 188–190 °C). ^1^H-NMR (400 MHz, CDCl_3_): δ = 1.06 (s, 3 H, CH_3_) 1.62 (s, 3 H, CH_3_), 3.18 (d, *J* = 16.0 Hz, 1 H, 9-H_a_), 3.88 (d, *J* = 16.0 Hz, 1 H, 9-H_b_), 4.09 (m, 1 H, 3-H_a_), 4.29 (dd, *J* = 16.0 Hz and 4.0 Hz, 1 H, 3-H_b_), 5.04 (s, 1H, 15a-H), 5.42 (dd, *J* = 12.0 Hz and 4.0 Hz, 1 H, 2-H), 6.81 and 6.89 (2 × d, *J* = 8.0 Hz, 2 H, 5-H and 19-H), 7.15-7.24 (m, 4 H, Ar-H), 7.26-7.32 (m, 3 H, Ar-H), 7.34–7.58 (m, 3 H, Ar-H), 8.12 (m, 2 H, 1′-H, 8-H). ^13^C-NMR (100 MHz): δ = 27.0 (CH_3_), 30.5 (CH_3_), 35.7 (C-9), 47.0 (C-3), 50.1 (C-10), 70.6 (C-15a), 80.1 (C-2), 105.05 (C-13), 110.1 (C-19) 119.5 (C-5), 124.3 (C-17), 124.9 (C-3′), 125,1 (C-1′), 125.2 (C-6), 125.5 (C-8), 126.4 (C-8a), 126.5 (C-6′), 126.6 (C-7′), 126.8 (C-15b), 127.6 (C-4′), 128.1 (C-5′), 128.4 (C-8), 131.0 (C-8′), 131.4 (C-16), 132.9 (C-8a’), 133.3 (C-4a’), 134.6 (C-7), 137.9 (C-2′), 147.7 (C-19a), 152.2 (C-4a), 164.5 (C-15), 168.2 (C-11). IR (KBr): 1201, 1320, 1507, 1736, 1773, 3057 cm^−1^. HRMS-ESI (*m*/*z*): [M + Na]^+^ calc’d for C_32_H_20_N_2_O_7_Na, 573.164; found: 573.163.

*(4E)-N-Hydroxy-2,3-dihydro-4H-chromen-4-imine* (**13**): Hydroxylamine hydrochloride (2.81 g, 40.44 mmol) was added to the stirred solution of chroman-4-one (**7a**, 3.0 g, 20.25 mmol) in pyridine (25 mL) and the mixture was stirred for 1.5 h. Cool water (100 mL) was added to the reaction mixture and the aqueous phase was extracted with ethyl-acetate (3 × 50 mL). The combined organic layers were dried over MgSO_4_, filtered and concentrated under reduced pressure. The residue was purified by column chromatography on silica gel (hexane/EtOAc 3:1) to give **13** as white solid (3.1 g, 94%, mp 140–141 °C). ^1^H NMR (360 MHz, CDCl_3_): δ = 3.01 (m, *J* = 6.1 Hz, 2 H, 3-H), 4.25 (m, *J* = 6.1 Hz, 2 H, 2-H), 6.89–6.96 (m, 2 H, 6-H, 8-H), 7.25 (m, 1 H, 7-H), 7.81 (dd, *J* = 9.7 Hz and 1.8 Hz, 1 H, 5-H), 9.39 (br s, 1 H, OH). ^13^C NMR (90 MHz, CDCl_3_): δ = 23.5 (C-3), 64.9 (C-2), 117.8 (C-8), 118.1 (C-5a), 121.5 (C-6), 123.9 (C-5), 131.2 (C-7), 149.9 (C-4), 156.7 (C-8a). IR (KBr): 1046, 1253, 1454, 1603, 3263 cm^−1^. HRMS-ESI (*m*/*z*): [M + H]^+^ calc’d for C_9_H_9_NO_2_H, 164.071; found: 164.071.

*2,3-Dihydro-1,5-benzoxazepin-4(5H)-one* (**15**): The solution of **13** (1.0 g, 6.13 mmol) in dry dichloromethane (60 mL) was cooled to 5 °C and added to DIBAL-H (40 mL, 1.0M in hexane) under argon atmosphere and the mixture was stirred for 24 h. The reaction mixture was diluted with ethyl-acetate (20 mL), and filtered through celite bench. The organic layer was dried over MgSO_4_, filtered and concentrated under reduced pressure to afford **15** as brown solid (883 mg, 97%, mp 48–52 °C). ^1^H NMR (400 MHz, CDCl_3_): δ = 1.98 (m, *J* = 5.6 Hz, 2H, 3-H), 3.19 (m, *J* = 5.6 Hz, 2 H, 4-H), 3.57 (br s, 1 H, -NH), 4.07 (m, *J* = 5.6 Hz, 2 H, 2-H), 6.73 (dd, *J* = 8.8 Hz and 1.2 Hz, 1 H, 6-H), 6.76 (m, 1 H, 7-H), 6.85 (m, 1 H, 8-H), 6.96 (dd, *J* = 9.2 Hz and 1.2 Hz, 1 H, 9-H). ^13^C NMR (100 MHz, CDCl_3_): δ = 31.9 (C-3), 45.9 (C-4), 71.4 (C-2), 119.5 (C-9), 120.7 (C-6), 121.8 (C-8), 123.3 (C-7), 142.0 (C-6a), 150.1 (C-9a). IR (KBr): 1053, 1250, 1595, 2952, 3282. HRMS-ESI (*m*/*z*): [M + Na]^+^ calc’d for C_9_H_11_NONa, 172.074; found: 172.074.

*2-(3,4-Dihydro-1,5-benzoxazepin-5(2H)-yl)benzaldehyde* (**4**): To the stirred solution of **15** (100 mg, 0.67 mmol) in dry 1,4-dioxane (4 mL), Pd(OAc)_2_ (11 mg, 0.05 mmol), 2-dicyclohexylphosphino-2′,6′-dimetoxybiphenyl (28 mg, 0.07 mmol), Cs_2_CO_3_ (328 mg, 1.00 mmol) and 2-bromobenzaldehyde (0.12 mL, 1.00 mmol) were added and the mixture was refluxed for 18 h under argon atmosphere. The reaction mixture was concentrated under reduced pressure and residue was purified by column chromatography on silica gel (hexane/EtOAc 8:1) to give **4** as yellow solid (97 mg, 87%, mp 89–92 °C). ^1^H NMR (400 MHz, CDCl_3_): δ = 2.07 (m, *J* = 5.6 Hz, 2H, 3-H), 3.83 (m, *J* = 5.6 Hz, 2 H, 4-H), 4.24 (m, *J* = 5.6 Hz, 2 H, 2-H), 6.52 (dd, *J* = 9.2 Hz and 1.2 Hz, 1 H, 9-H), 6.79 (m, 1H, 7-H), 6.88 (m, 1 H, 8-H), 7.01 (dd, *J* = 9.2 Hz and 1.2 Hz, 1 H, 6-H), 7.17 (m, *J* = 7.2 Hz, 1 H, 4′-H), 7.25 (d, *J* = 8.4 Hz, 1 H, 6′-H), 7.56 (m, 1 H, 5′-H), 7.81 (dd, *J* = 8.4 Hz and 1.2 Hz, 1 H, 3′-H), 10.14 (s, 1H, -CHO). ^13^C NMR (100 MHz, CDCl_3_): δ = 29.9 (C-3), 52.6 (C-4), 70.1 (C-2), 121.9 (C-9), 123.4 (C-6), 123.5 (C-6′), 123.6 (C-4′), 124.0 (C-8), 124.7 (C-7), 129.1 (C-3′), 129.9 (C-2′), 134.8 (C-5′), 142.6 (C-6a), 152.2 (C-9′), 152.3 (C-1′), 190.8 (CHO). IR (KBr): 1059, 1251, 1455, 1686. HRMS-ESI (*m*/*z*): [M + Na]^+^ calc’d for C_16_H_15_NO_2_Na, 276.100; found: 276.095.

*[2-(3,4-Dihydro-1,5-benzoxazepin-5(2H)-yl)benzylidene]propanedinitrile* (**20**): To the stirred solution of **4** (100 mg, 0.39 mmol) in dry toluene (3 mL), dimethylamine-hydrochloride (128 mg, 1.58 mmol) and malononitrile (156 mg, 2.37 mmol) were added and the mixture was stirred for 48 h. Water (10 mL) and dichloromethane (20 mL) were added and two layers were separated. The aqueous phase was extracted with dichloromethane (2 × 10 mL). The combined organic layers were washed with concentrated NaHCO_3_ solution (3 × 10 mL), dried over MgSO_4_, filtered and concentrated under reduced pressure. The product was isolated as red solid (115 mg, 3.38 mmol, 96%, mp 118–121 °C).^1^H NMR (400 MHz, CDCl_3_): δ = 2.07 (m, 2 H, 3-H), 3.72 (m, 2 H, 4-H), 4.21 (m, 2 H, 2-H), 6.47 (d, *J* = 7.6 Hz, 1 H, 9-H), 6.87 (m, 1 H, 7-H), 7.01 (m, 1H, 8-H), 7.09 (m, 1H, 6-H), 7.16 (m, 1 H, 4′-H), 7.31 (d, *J* = 7.6 Hz, 1 H, 6′-H), 7.57 (t, *J* = 7.6 Hz, 1 H, 5′-H), 7.82 (m, 2 H, 3′-H, 10-H). ^13^C NMR (90 MHz, CDCl_3_): δ = 30.3 (C-3), 51.7 (C-4), 70.6 (C-2), 82.0 (C-11), 112.3 (-CN), 113.4 (-CN), 122.4 (C-9), 122.7 (C-6′), 123.1 (C-6), 124.1 (C-4′), 125.1 (C-8), 125.3 (C-7), 125.4 (C-2′), 129.5 (C-5′), 134.5 (C-3′), 142.6 (C-6a), 151.0 (C-9a), 153.7 (C-1′), 158.6 (C-10). IR (KBr): 1052, 1253, 1455, 2230.

*5-(2,3-Dihydro-1H,8H-[1,4]oxazepino[2,3,4-de]acridin-8-yl)-1,3-dimethylpyrimidine-2,4,6(1H,3H,5H)-trione* (**6**): To the solution of **4** (100 mg, 0.40 mmol) in chloroform (6 mL), anhydrous MgSO_4_ (150 mg, 1.25 mmol) and 1,3-dimethylbarbituric acid (370 mg, 2.42 mmol) were added and the mixture was stirred for 10 days. The MgSO_4_ was filtered off and chloroform was removed under reduced pressure. The residue was taken up in chloroform (30 mL) and extracted with concentrated NaHCO_3_ solution (3 × 10 mL), dried over MgSO_4_, filtered and concentrated under reduced pressure. The resultant product was isolated as purple solid (100 mg, 64%). ^1^H NMR (400 MHz, CDCl_3_): δ = 2.26 (m, 1 H, 3-H_a_), 2.36 (m, 1 H, 3-H_b_), 3.04 (s, 3 H, NMe), 3.10 (s, 3 H, NMe), 3.61 (d, *J* = 4.4 Hz, 1 H, 10-H), 3.93 (m, 1 H, 2-H_a_), 4.10 (m, 2 H, 4-H), 4.38 (m, 1 H, 2-H_b_), 4.90 (d, *J* = 4.4 Hz, 1 H, 10-H), 6.90–6.96 (m, 6-H, 7-H, 8-H, 11-H, 13-H, 5H), 7.03 (d, *J* = 6.8 Hz, 1 H, 9-H), 7.29 (m, 1 H, 12-H). ^13^C NMR (100 MHz, CDCl_3_): δ = 27.7 (C-3), 28.0 (N-CH_3_), 28.2 (N-CH_3_), 47.4 (C-4), 49.5 (C-10), 59.0 (C-1′), 70.4 (C-2), 112.3 (C-13), 120.4 (C-9a), 120.6 (C-6), 120.9 (C-8), 122.4 (C-11), 122.6 (C-12), 125.1 (C-10b), 128.1 (C-7), 129.2 (C-9), 134.5 (C-10a), 143.8 (C-13a), 149.2 (C-5a), 151.2 (C-4′), 166.4 (C-6′), 167.1 (C-2′).

## 4. Conclusions

*N*-aryl-1,4- and 1,5-benzoaxazepine derivatives were prepared with different substitution patterns in short sequences and they were reacted with active methylene reagents to induce domino cyclization reactions. The *N*-aryl-1,4-benzoaxazepines reacted with a Knoevenagel-[1,5]-hydride shift-cyclization cascade providing condensed chiral *O,N*-heterocycles in regio- and diastereoselective manner. Enantiomers of two products were separated by chiral HPLC, HPLC-ECD specra were recorded and characteristic ECD transitions were correlated with the absolute configuration by TDDFT-ECD calculations. The regioisomeric 1,5-benzoaxazepine derivative reacted with 1,3-dimethylbarbituric acid in a domino Knoevenagel-C(sp^2^)-H functionalization reaction resulting in a condensed acridane derivative. Neuroprotective and AChE inhibitory activity of the products were tested. The condensed 1,4-benzoxazepine products did not have significant neuroprotective activity, which on the basis of our previous results, suggested that lack of the C-2 phenyl group or replacement by naphthyl groups resulted in the loss of activity. The condensed acridane derivative, containing a 1,5-benzoxazepine moiety, was found to have AChE inhibitory activity with 6.98 × 10^−6^ mol/L IC_50_ value.

## Supplementary Materials

The following are available online. Figure S1–S74: ^1^H and ^13^C NMR and IR spectra of synthetic derivatives, Table S1: Organocatalytic transformations of **1a** to *trans*-**10a** (columns A) and **1a** to **11a** (columns B), Figure S75: Concentration-dependent curve of **6** for the inhibition of AChE activity.
