# Supplementary material for: [1,5]-Hydride Shift-Cyclization versus C(sp2)-H Functionalization in the Knoevenagel-Cyclization Domino Reactions of 1,4- and 1,5-Benzoxazepines
Source: Molecules. 2020 Mar 11;25(6):1265. doi: 10.3390/molecules25061265 (PMC7144003; doi:10.3390/molecules25061265)
Supplement: Supplementary file 1 [file molecules-25-01265-s001.pdf]

Supporting information

[1,5]-Hydride shift-cyclization *versus* C(sp^2)-H Functionalization in the Knoevenagel-Cyclization Domino Reactions of 1,4- and 1,5-Benzoxazepines

Dóra Szalóki Vargáné [1,2,†], László Tóth [1,3,†], Balázs Buglyó [1], Attila Kiss-Szikszai [1], Attila Mándi [1], Péter Mátyus [4], Sándor Antus [1,*], Yinghan Chen [5], Dehai Li [5], Lingxue Tao [6], Haiyan Zhang [6] and Tibor Kurtán [1,*]

1 Department of Organic Chemistry, University of Debrecen, Debrecen, P. O. Box 400, Debrecen 4002, Hungary; szalokido@gmail.com (D.S.V.); tothlaszlochemist@gmail.com (L.T.); buglyo.balazs@science.unideb.hu (B.B.); kiss.attila@science.unideb.hu (A.K.-S.); mandi.attila@science.unideb.hu (A.M.)

2 Doctoral School of Chemistry, University of Debrecen, Egyetem tér 1, Debrecen 4032, Hungary

3 Department of Organic Chemistry, Semmelweis University, Budapest 1094, Hungary

4 Institute of Digital Health Sciences, Faculty of Health and Public Services, Semmelweis University, Ferenc tér 15, Budapest 1094, Hungary; peter.maty@gmail.com

5 Key Laboratory of Marine Drugs, Chinese Ministry of Education, School of Medicine and Pharmacy, Ocean University of China, Qingdao 266003, China; qd_yinghan@163.com (Y.C.); dehaili@ouc.edu.cn (D.L.);

6 CAS Key Laboratory of Receptor Research, Shanghai Institute of Materia Medica, Chinese Academy of Sciences, 555 Zu Chong Zhi Road, Zhang Jiang Hi-Tech Park, Shanghai 201203, China; lingxuetao@simm.ac.cn (L.T.); hzhang@simm.ac.cn (H.Z.)

* Correspondences: kurtan.tibor@science.unideb.hu (T.K.), antus.sandor@science.unideb.hu (S.A.)

† These authors contributed equally to this work.

Table of content

Figure S1. ^{1}H-NMR spectrum of *rac*-**8a** measured in $CDCl_3$ (400 MHz)……………………..5

Figure S2. J-modulated ^{13}C-NMR spectrum of *rac*-**8a** measured in $CDCl_3$ (100 MHz)……...6

Figure S3. IR spectrum of *rac*-**8a** recorded as KBr disc…..7

Figure S4. ^{1}H-NMR spectrum of *rac*-**9a** measured in $CDCl_3$ (400 MHz)……………...……..8

Figure S5. J-modulated ^{13}C-NMR spectrum of *rac*-**9a** measured in $CDCl_3$ (100 MHz)….......9

Figure S6. IR spectrum of *rac*-**9a** recorded as KBr disc…..10

Figure S7. ^{1}H-NMR spectrum of *rac*-**1a** measured in $CDCl_3$ (400 MHz)…………………...11

Figure S8. J-modulated ^{13}C-NMR spectrum of *rac*-**1a** measured in $CDCl_3$ (100 MHz)…......12

Figure S9. IR spectrum of *rac*-**1a** recorded as KBr disc…..13

Figure S10. ^{1}H-NMR spectrum of *rac*-**12a** measured in $CDCl_3$ (400 MHz)……….…....…14

Figure S11. J-modulated ^{13}C-NMR spectrum of *rac*-**12a** measured in $CDCl_3$ (100 MHz).....15

Figure S12. IR spectrum of *rac*-**12a** recorded as KBr disc…..16

Figure S13. ^{1}H-NMR spectrum of *rac*-**10a** measured in $CDCl_3$ (400 MHz)……………...…17

Figure S14. J-modulated ^{13}C-NMR spectrum of *rac*-**10a** measured in $CDCl_3$ (100 MHz).....18

Figure S15. IR spectrum of *rac*-**10a** recorded as KBr disc…...19

Figure S16. ^{1}H-NMR spectrum of *rac*-**11a** measured in DMSO-d_6 (400 MHz)………...…20

Figure S17. J-modulated ^{13}C-NMR spectrum of *rac*-**11a** measured in DMSO-d_6 (100 MHz)……………………………………………………………………………21

Figure S18. IR spectrum of *rac*-**11a** recorded as KBr disc…...22

Figure S19. ^{1}H-NMR spectrum of *rac*-**7b** measured in $CDCl_3$ (360 MHz)..………………...23

Figure S20. J-modulated ^{13}C-NMR spectrum of *rac*-**7b** measured in $CDCl_3$ (90 MHz).........24

Figure S21. J-modulated ^{13}C-NMR spectrum of *rac*-**7b** measured in $CDCl_3$ (90 MHz)…......25

Figure S22. ^{1}H-NMR spectrum of *rac*-**7c** measured in $CDCl_3$ (360 MHz)...........................26

Figure S23. J-modulated ^{13}C-NMR spectrum of *rac*-**7c** measured in $CDCl_3$ 90 MHz)…......27

Figure S24. J-modulated ^{13}C-NMR spectrum of *rac*-**7c** measured in $CDCl_3$ (90 MHz)..…...28

Figure S25. ^{1}H-NMR spectrum of *rac*-**8b** measured in $CDCl_3$ (400 MHz)………..……...29

Figure S26. J-modulated ^{13}C-NMR spectrum of *rac*-**8b** measured in $CDCl_3$ (100 MHz)…...30

Figure S27. J-modulated ^{13}C-NMR spectrum of *rac*-**8b** measured in $CDCl_3$ (100 MHz)…...31

Figure S28. IR spectrum of *rac*-**8b** recorded as KBr disc…...32

Figure S29. ^{1}H-NMR spectrum of *rac*-**8c** measured in $CDCl_3$ (400 MHz)………………...33

Figure S30. J-modulated ^{13}C-NMR spectrum of *rac*-**8c** measured in $CDCl_3$ (100 MHz)…...34

Figure S31. J-modulated ^{13}C-NMR spectrum of *rac*-**8c** measured in $CDCl_3$ (100 MHz)…...35

Figure S32. IR spectrum of *rac*-**8c** recorded as KBr disc…...36

Figure S33. ^{1}H-NMR spectrum of *rac*-**9b** measured in $CDCl_3$ (400 MHz)…….…………...37

Figure S34. J-modulated ^{13}C-NMR spectrum of *rac*-**9b** measured in $CDCl_3$ (100 MHz).......38

Figure S35. J-modulated ^{13}C-NMR spectrum of *rac*-**9b** measured in $CDCl_3$ (100 MHz)…...39

Figure S36. IR spectrum of *rac*-**9b** recorded as KBr disc…...40

Figure S37. ^{1}H-NMR spectrum of *rac*-**9c** measured in $CDCl_3$ (400 MHz)………..…..…...41

Figure S38. J-modulated ^{13}C-NMR spectrum of *rac*-**9c** measured in $CDCl_3$ (100 MHz)…...42

Figure S39. J-modulated ^{13}C-NMR spectrum of *rac*-**9c** measured in $CDCl_3$ (100 MHz).......43

Figure S40. IR spectrum of *rac*-**9c** recorded as KBr disc…..44

Figure S41. ^{1}H-NMR spectrum of *rac*-**1b** measured in $CDCl_3$ (400 MHz)……………........45

Figure S42. J-modulated ^{13}C-NMR spectrum of *rac*-**1b** measured in $CDCl_3$ (100 MHz)…...46

Figure S43. J-modulated ^{13}C-NMR spectrum of *rac*-**1b** measured in $CDCl_3$ (100 MHz)…...47

Figure S44. IR spectrum of *rac*-**1b** recorded as KBr disc…..48

Figure S45. ^{1}H-NMR spectrum of *rac*-**1c** measured in $CDCl_3$ (400 MHz)……………........49

Figure S46. J-modulated ^{13}C-NMR spectrum of *rac*-**1c** measured in $CDCl_3$ (100 MHz).......50

Figure S47. J-modulated ^{13}C-NMR spectrum of *rac*-**1c** measured in $CDCl_3$ (100 MHz)…...51

Figure S48. J-modulated ^{13}C-NMR spectrum of *rac*-**1c** measured in $CDCl_3$ (100 MHz)…...52

Figure S49. IR spectrum of *rac*-**1c** recorded as KBr disc…..53

Figure S50. ^{1}H-NMR spectrum of *rac*-**10b** measured in DMF-d_7 (400 MHz)………….......54

Figure S51. J-modulated ^{13}C-NMR spectrum of *rac*-**10b** measured in DMF-d_7 (100 MHz)..55

Figure S52. J-modulated ^{13}C-NMR spectrum of *rac*-**10b** measured in DMF-d_7 (100 MHz)..56

Figure S53. ^{1}H-NMR spectrum of *rac*-**11b** measured in $CDCl_3$ (400 MHz)……………........57

Figure S54. J-modulated ^{13}C-NMR spectrum of *rac*-**11b** measured in $CDCl_3$ (100 MHz).....58

Figure S55. J-modulated ^{13}C-NMR spectrum of *rac*-**11b** measured in $CDCl_3$ (100 MHz)....59

Figure S56. IR spectrum of *rac*-**11b** recorded as KBr disc…...60

Figure S57. ^{1}H-NMR spectrum of *rac*-**11c** measured in $CDCl_3$ (400 MHz)……………........61

Figure S58. J-modulated ^{13}C-NMR spectrum of *rac*-**11c** measured in $CDCl_3$ (100 MHz).....62

Figure S59. J-modulated ^{13}C-NMR spectrum of *rac*-**11c** measured in $CDCl_3$ (100 MHz).....63

Figure S60. IR spectrum of *rac*-**11c** recorded as KBr disc……………………………………64

Figure S61. ^{1}H-NMR spectrum of **13** measured in $CDCl_3$ (360 MHz)..................................65

Figure S62. J-modulated ^{13}C-NMR spectrum of **13** measured in $CDCl_3$ (90 MHz)………..66

Figure S63. IR spectrum of **13** recorded as KBr disc……………………………………… 67

Figure S64. ^{1}H-NMR spectrum of **15** measured in $CDCl_3$ (400 MHz)……………………........68

Figure S65. J-modulated ^{13}C-NMR spectrum of **15** measured in $CDCl_3$ (100 MHz)….........69

Figure S66. IR spectrum of **15** recorded as KBr disc………………………………………. 70

Figure S67. ^{1}H-NMR spectrum of **4** measured in $CDCl_3$ (400 MHz)……..…..………….........71

Figure S68. J-modulated ^{13}C-NMR spectrum of **4** measured in $CDCl_3$ (100 MHz)…...........72

Figure S69. IR spectrum of **4** recorded as KBr disc…..73

Figure S70. ^{1}H-NMR spectrum of **29** measured in $CDCl_3$ (360 MHz)……..…………........74

Figure S71. J-modulated ^{13}C-NMR spectrum of **20** measured in $CDCl_3$ (90 MHz)…….........75

Figure S72. IR spectrum of **20** recorded as KBr disc…..76

Figure S73. ^{1}H-NMR spectrum of **6** measured in $CDCl_3$ (400 MHz)……...………….........77

Figure S74. J-modulated ^{13}C-NMR spectrum of **6** measured in $CDCl_3$ (100 MHz)…...........78

Table S1. Organocatalytic transformations of **1a** to *trans*-**10a** (columns A) and **1a** to **11a** (columns B); reaction condition: $MgSO_4$/$CHCl_3$, 0.3 equivalent of organocatalyst, rt. ……..79

Figure S78. Concentration-dependent curve of **6** for the inhibition of AChE activity……..80

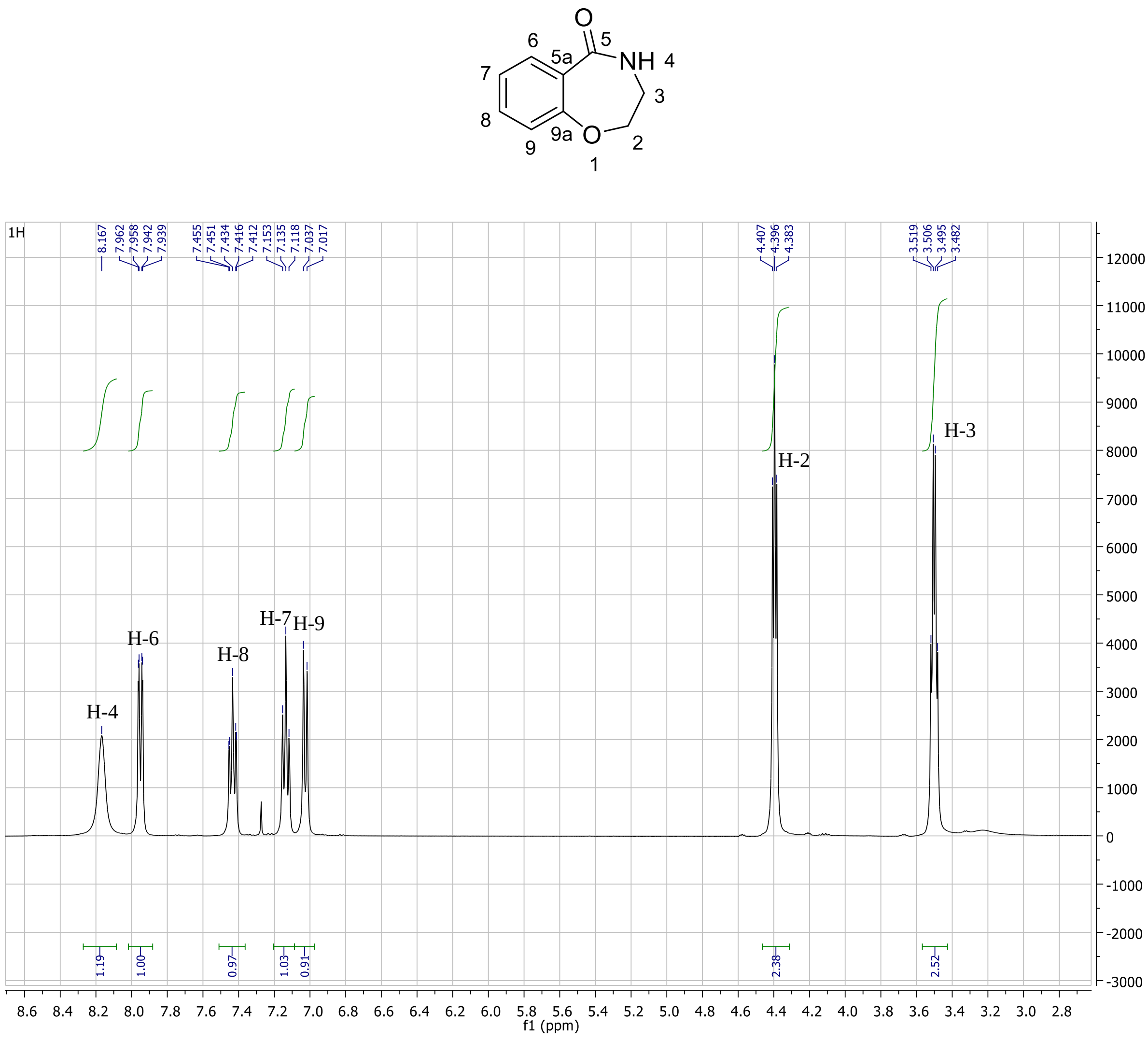

Figure S1. ^{1}H-NMR spectrum of *rac*-**8a** measured in $CDCl_3$ (400 MHz).

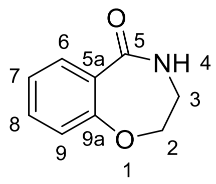

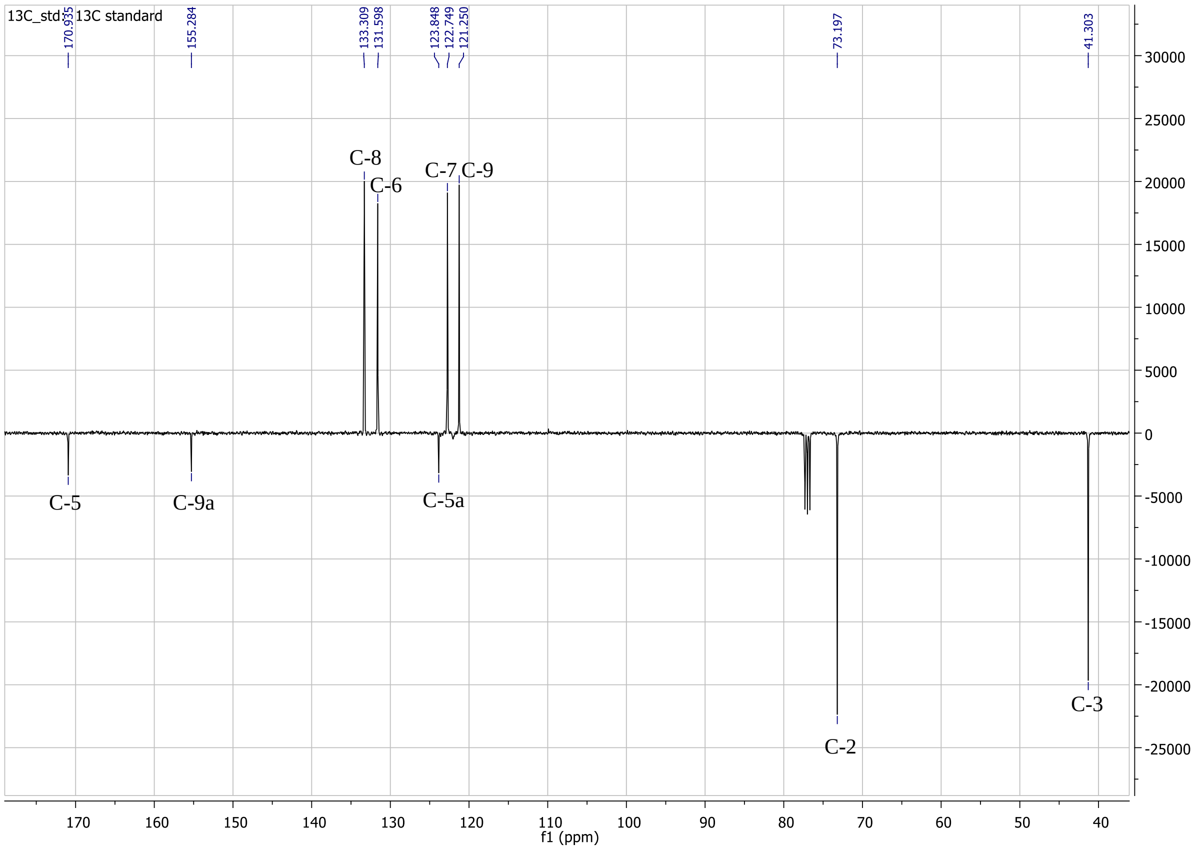

Figure S2. J-modulated ^{13}C-NMR spectrum of *rac*-**8a** measured in $CDCl_3$ (100 MHz).

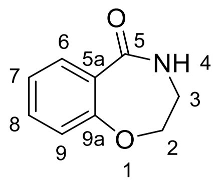

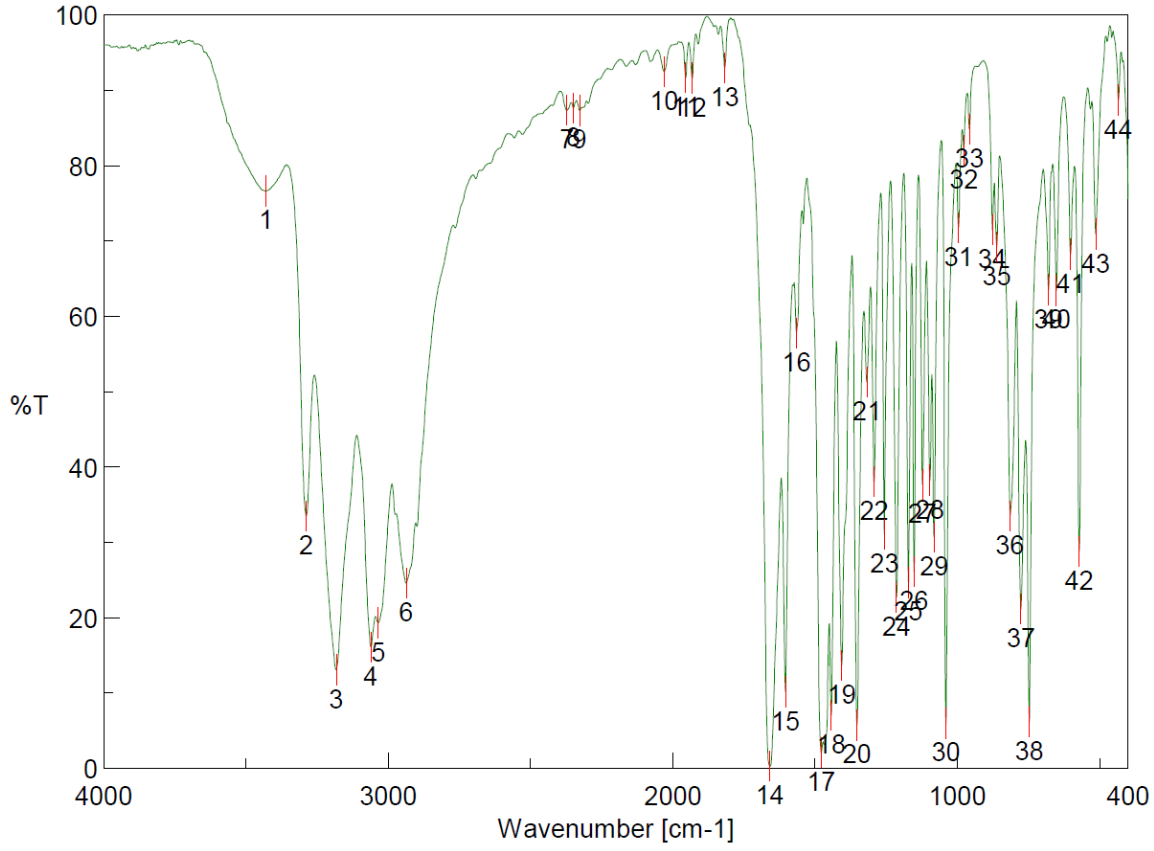

Figure S3. IR spectrum of *rac*-**8a** recorded as KBr disc.

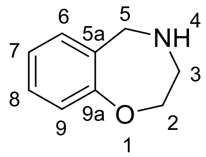

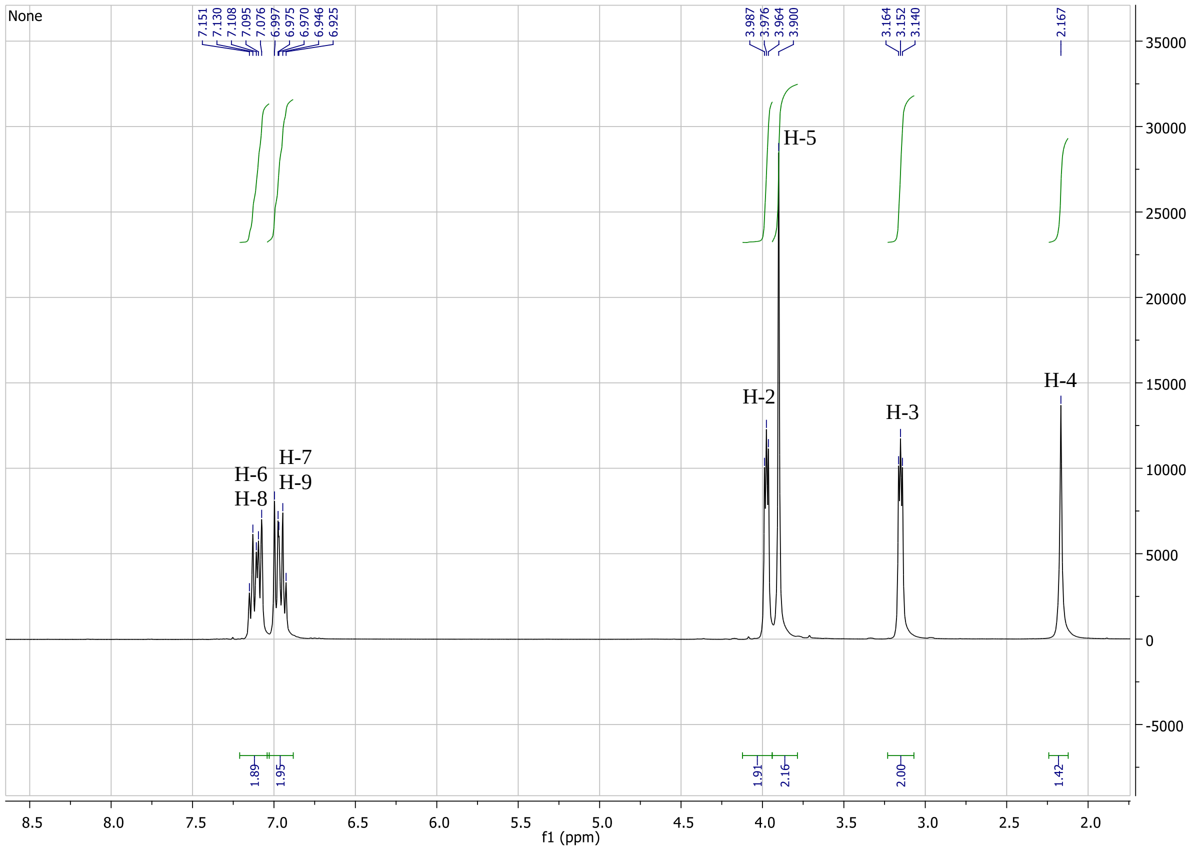

Figure S4.^{1}H-NMR spectrum of *rac*-**9a** measured in $CDCl_3$ (400 MHz).

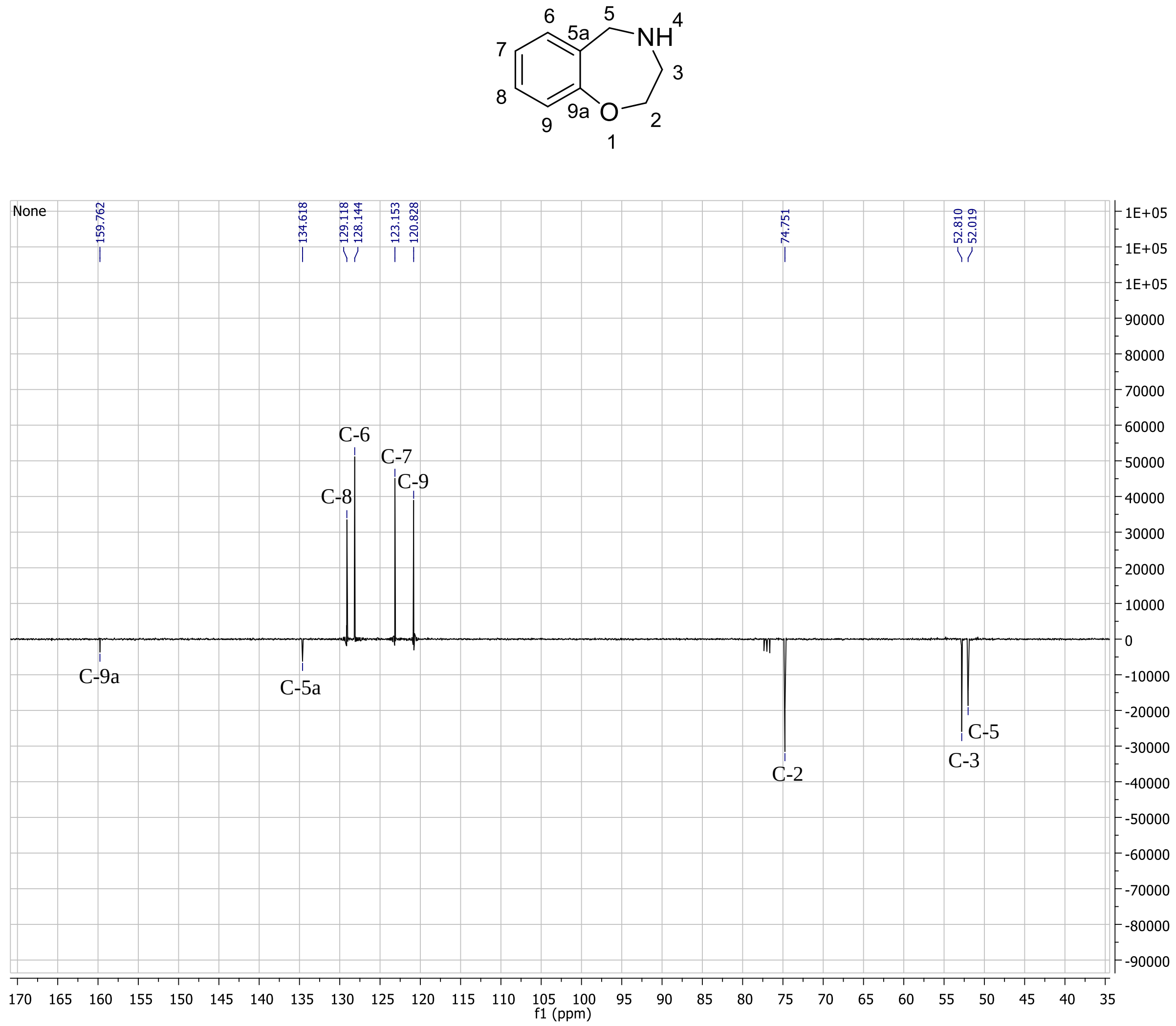

Figure S5. J-modulated ^{13}C-NMR spectrum of *rac*-**9a** measured in $CDCl_3$ (100 MHz).

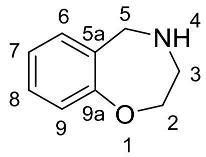

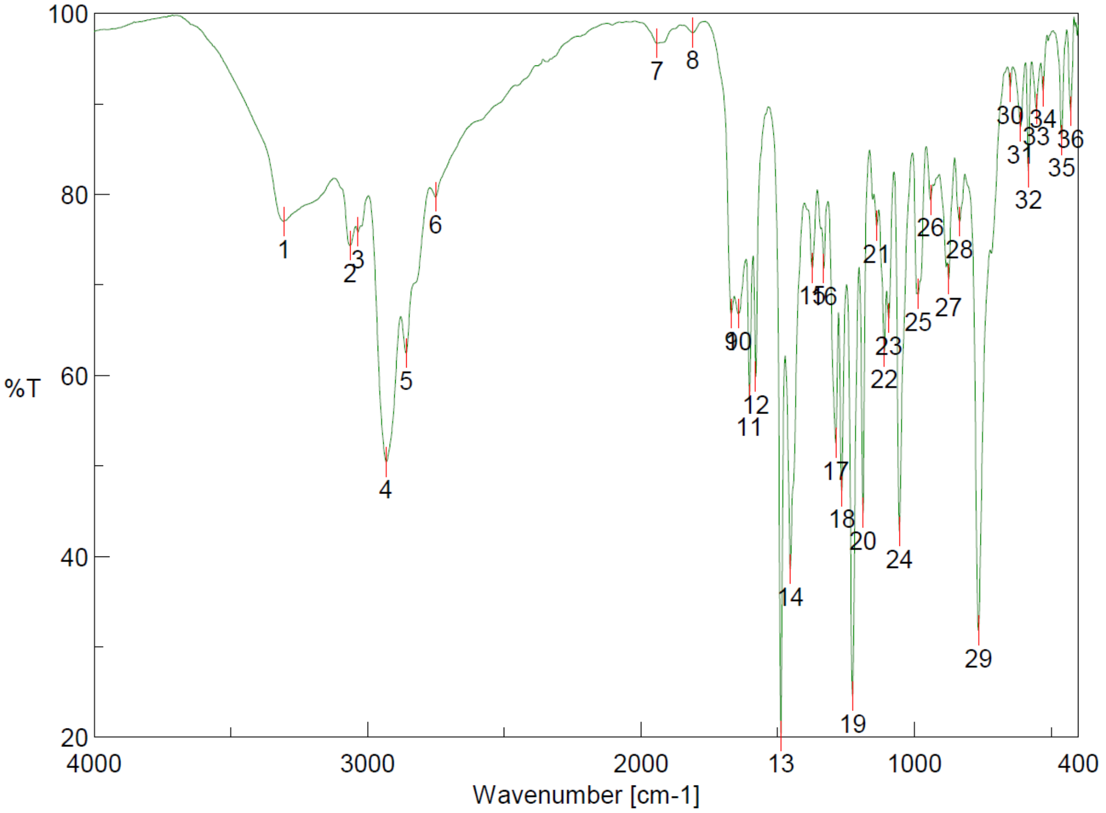

Figure S6. IR spectrum of *rac*-**9a** recorded as KBr disc.

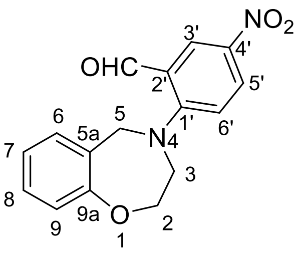

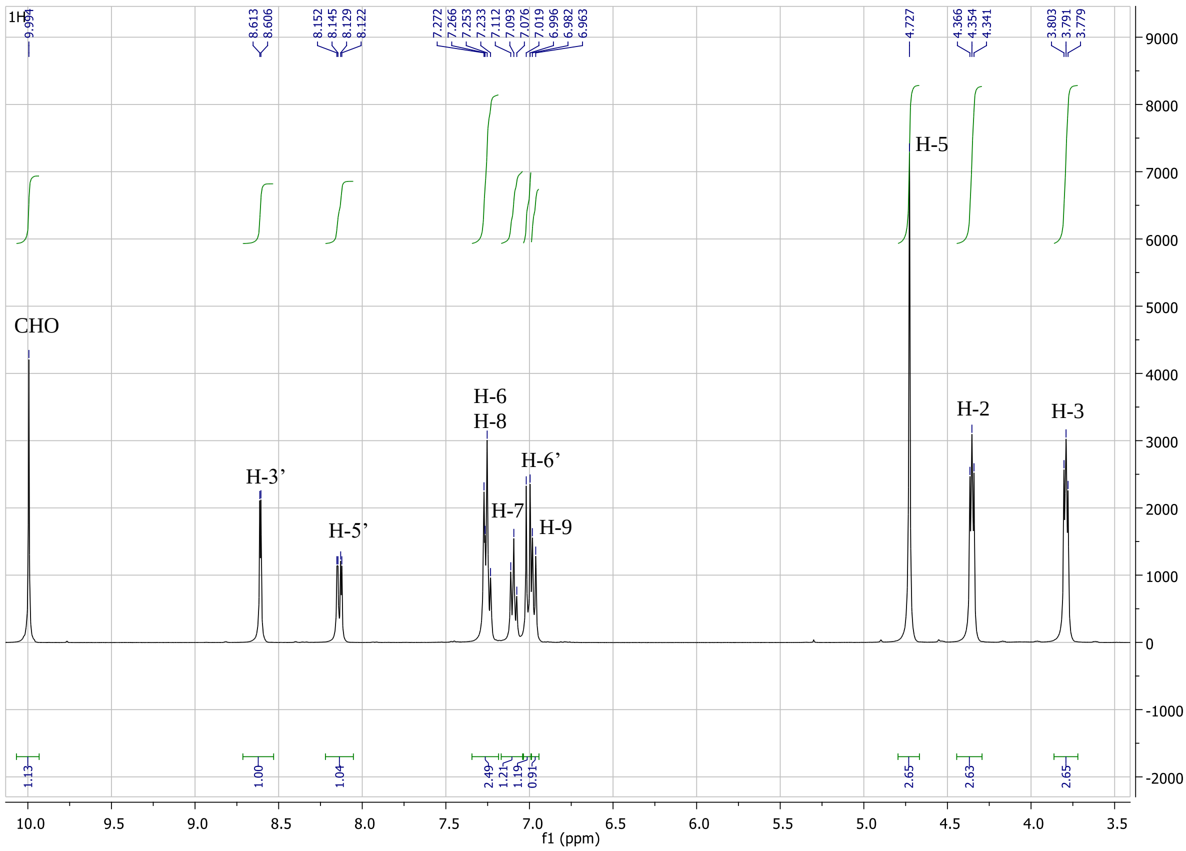

Figure S7. ^{1}H-NMR spectrum of *rac*-**1a** measured in $CDCl_3$ (400 MHz).

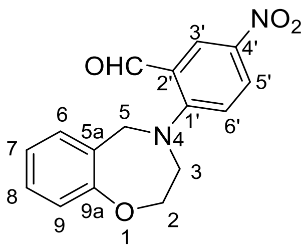

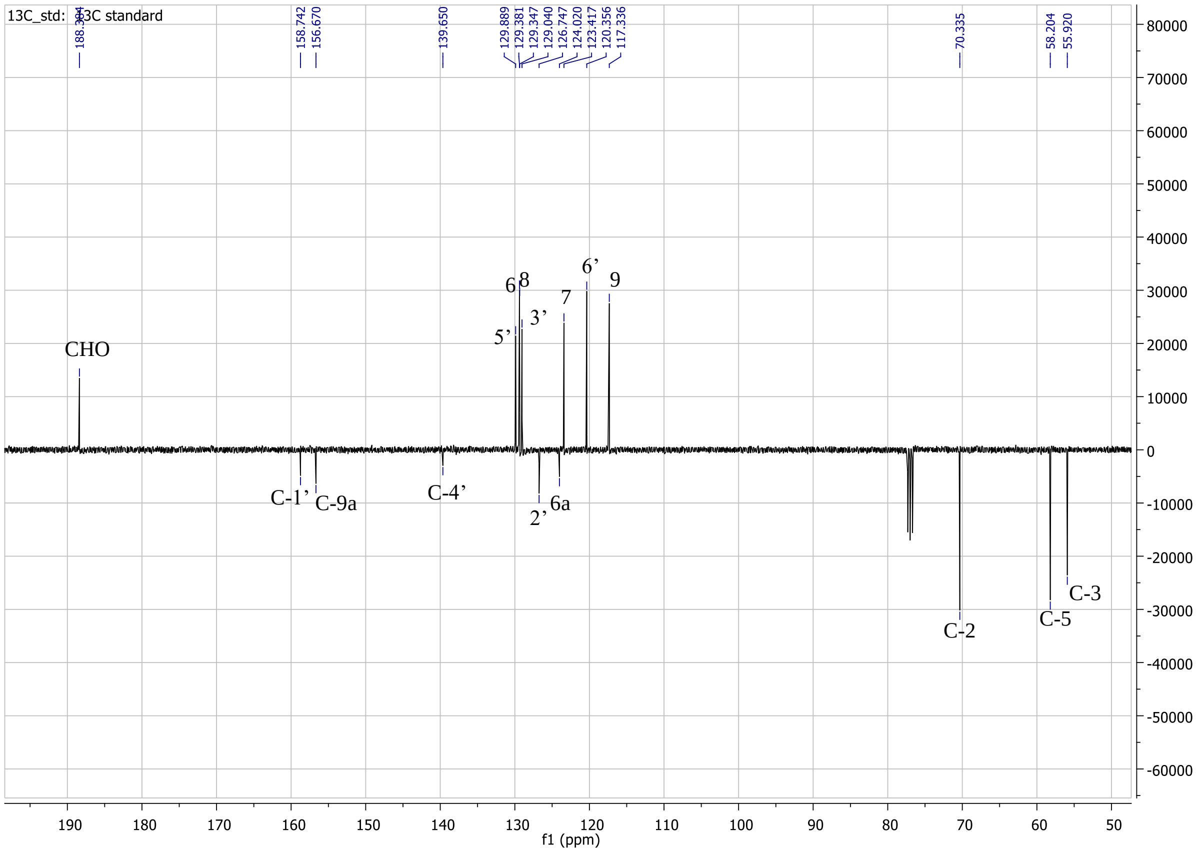

Figure S8. J-modulated ^{13}C-NMR spectrum of *rac*-**1a** measured in $CDCl_3$ (100 MHz).

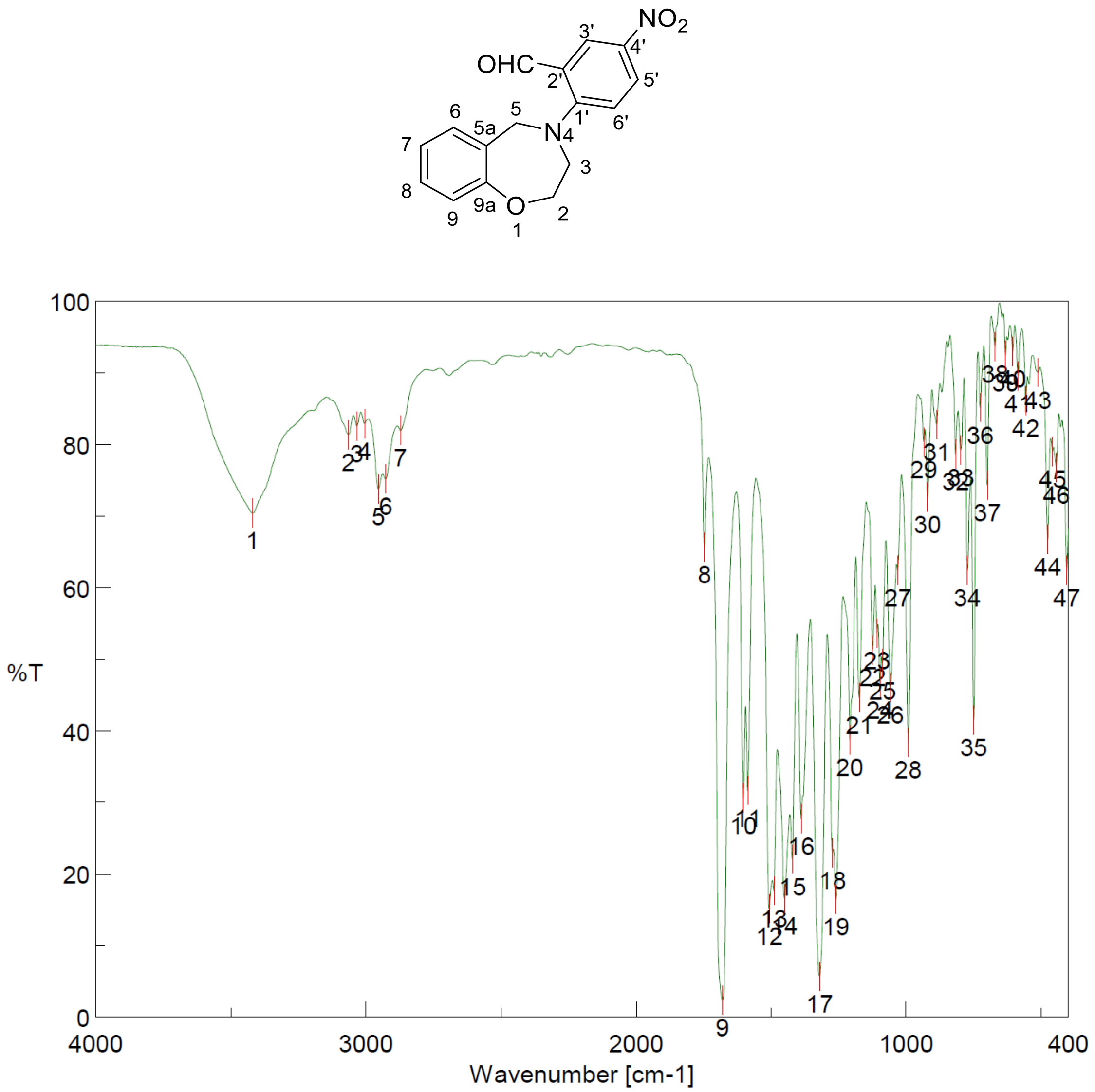

Figure S9. IR spectrum of *rac*-**1a** recorded as KBr disc.

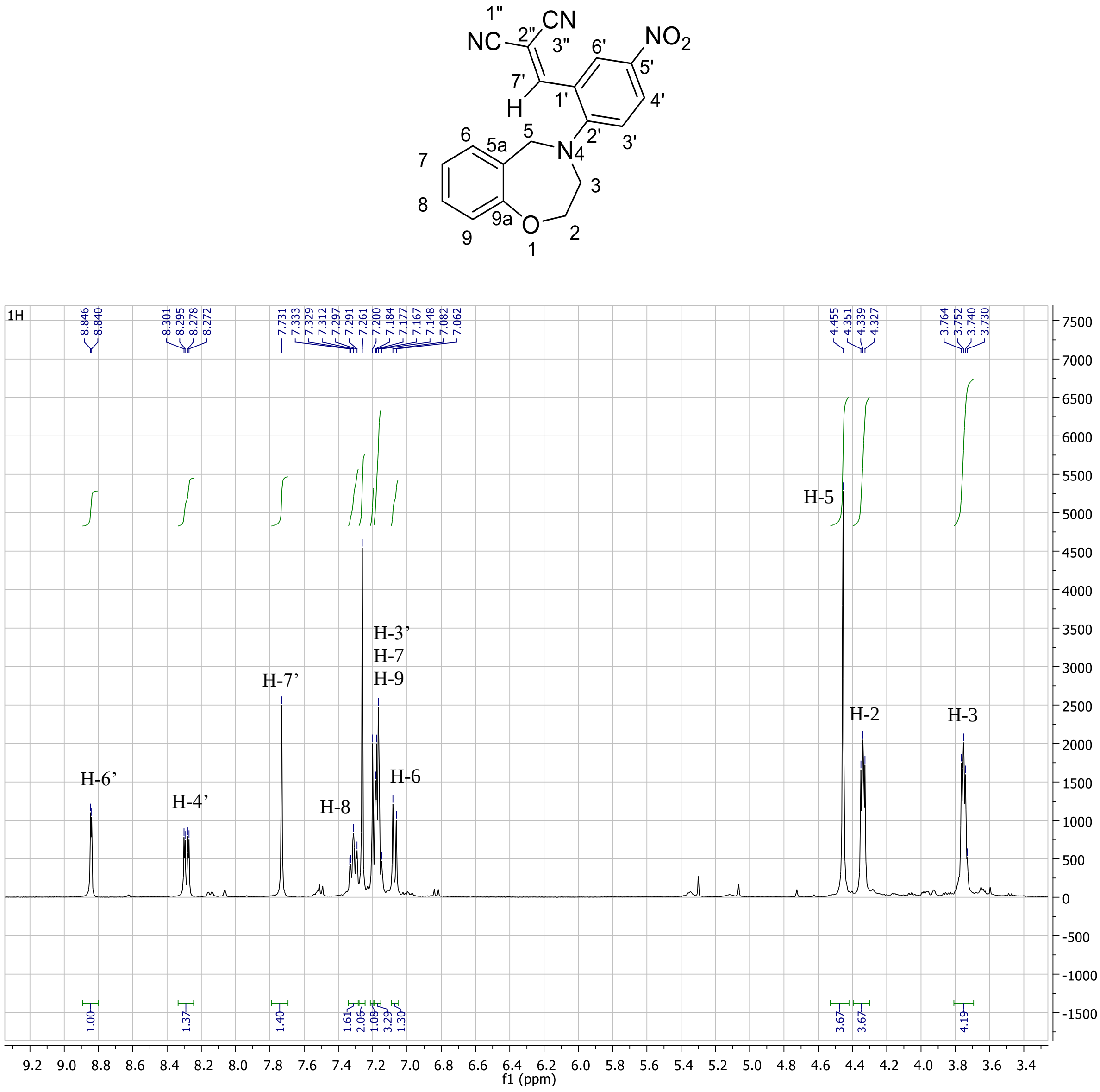

Figure S10. ^{1}H-NMR spectrum of *rac*-**12a** measured in $CDCl_3$ (400 MHz).

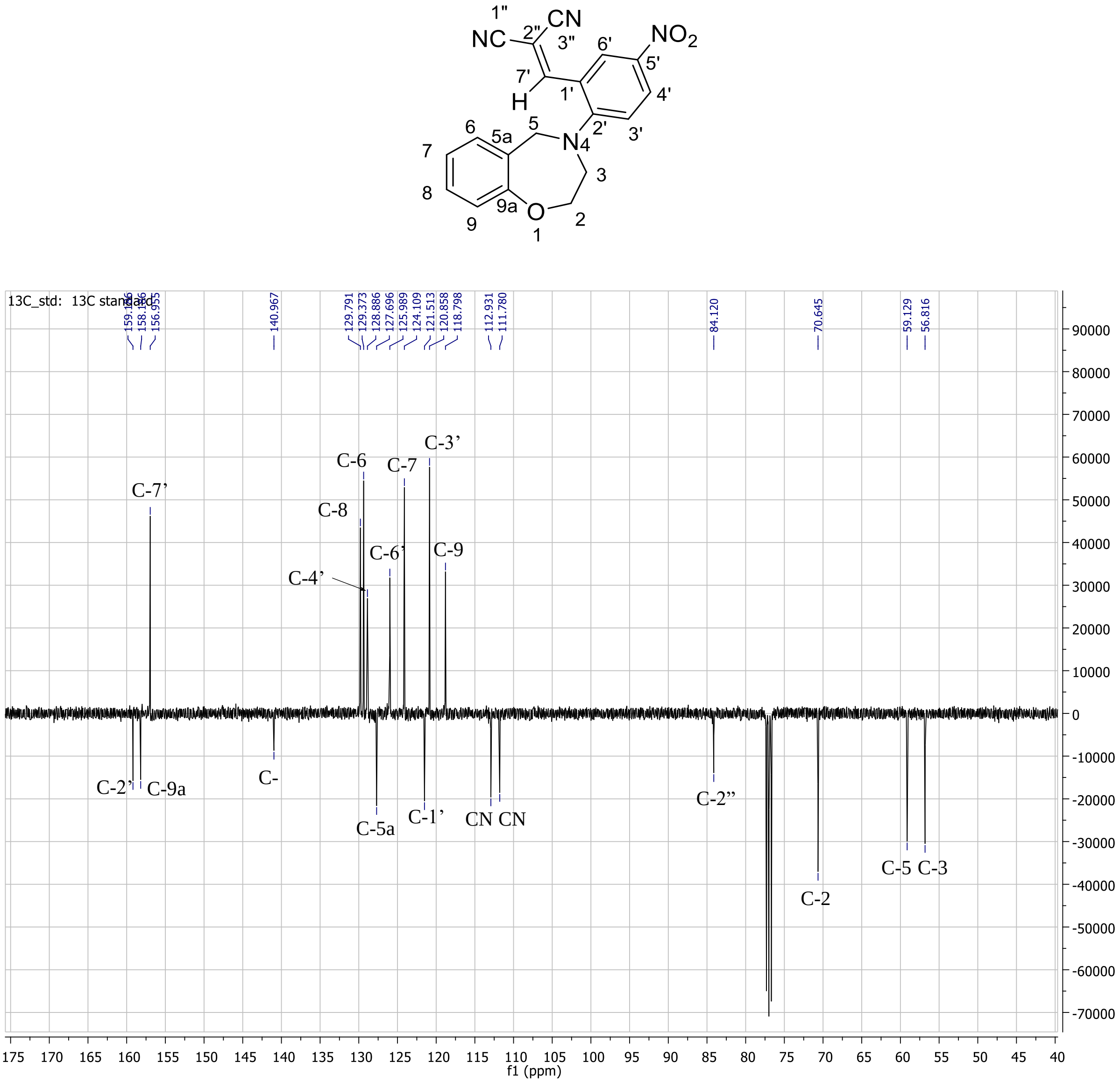

Figure S11. J-modulated ^{13}C-NMR spectrum of *rac*-**12a** mesured in $CDCl_3$ (100 MHz).

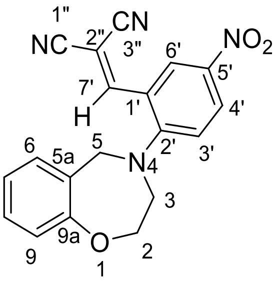

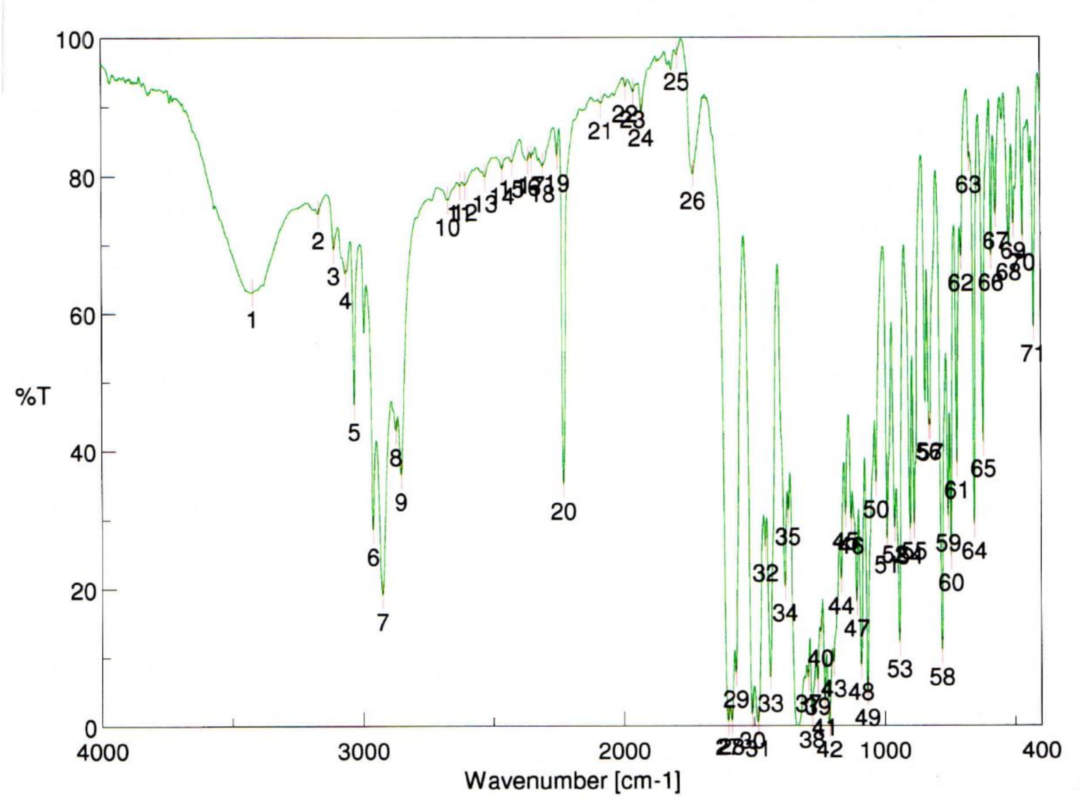

Figure S12. IR spectrum of *rac*-**12a** recorded as KBr disc.

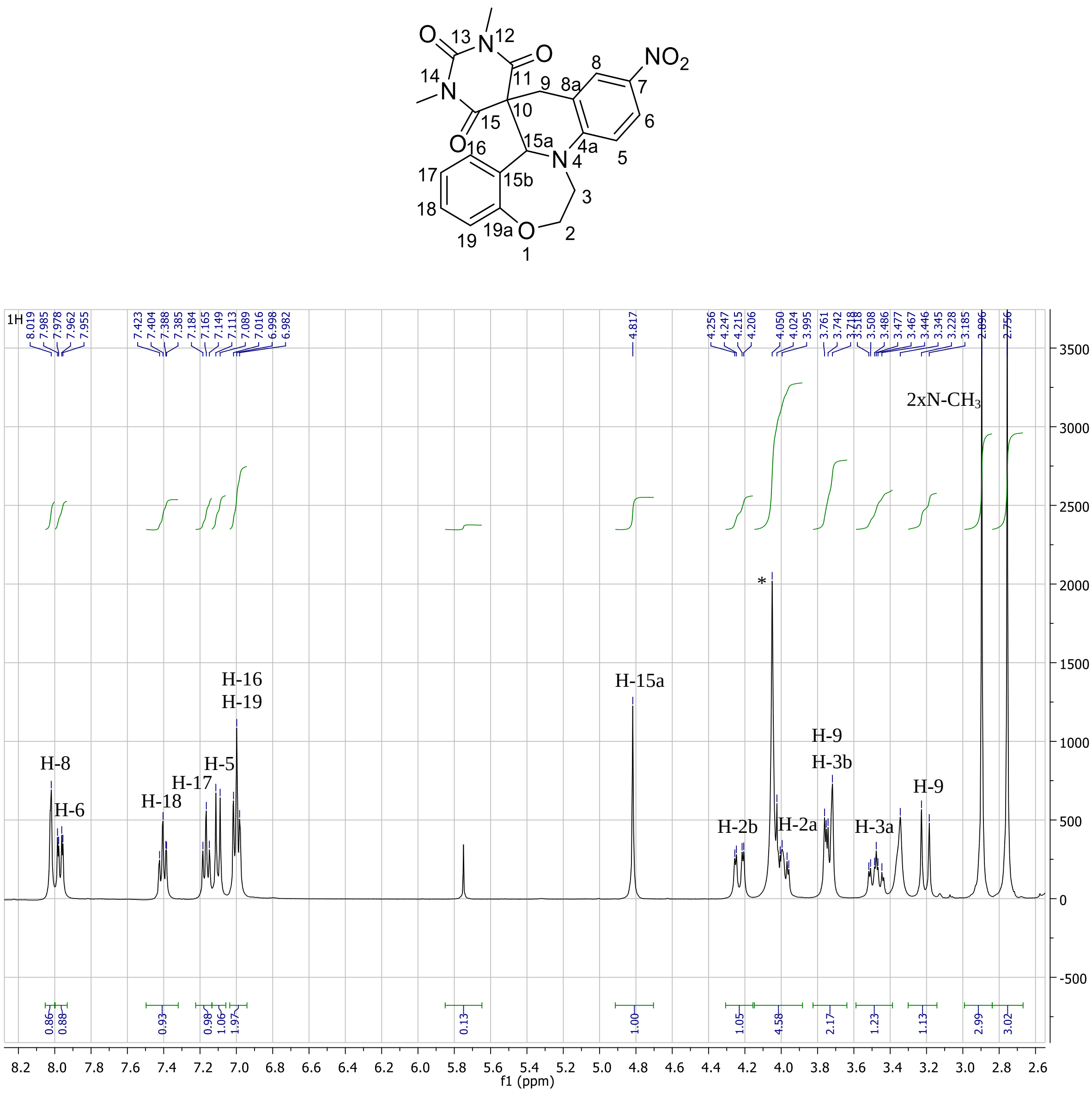

Figure S13. ^{1}H-NMR spectrum of *rac*-**10a** measured in $CDCl_3$ (400 MHz).

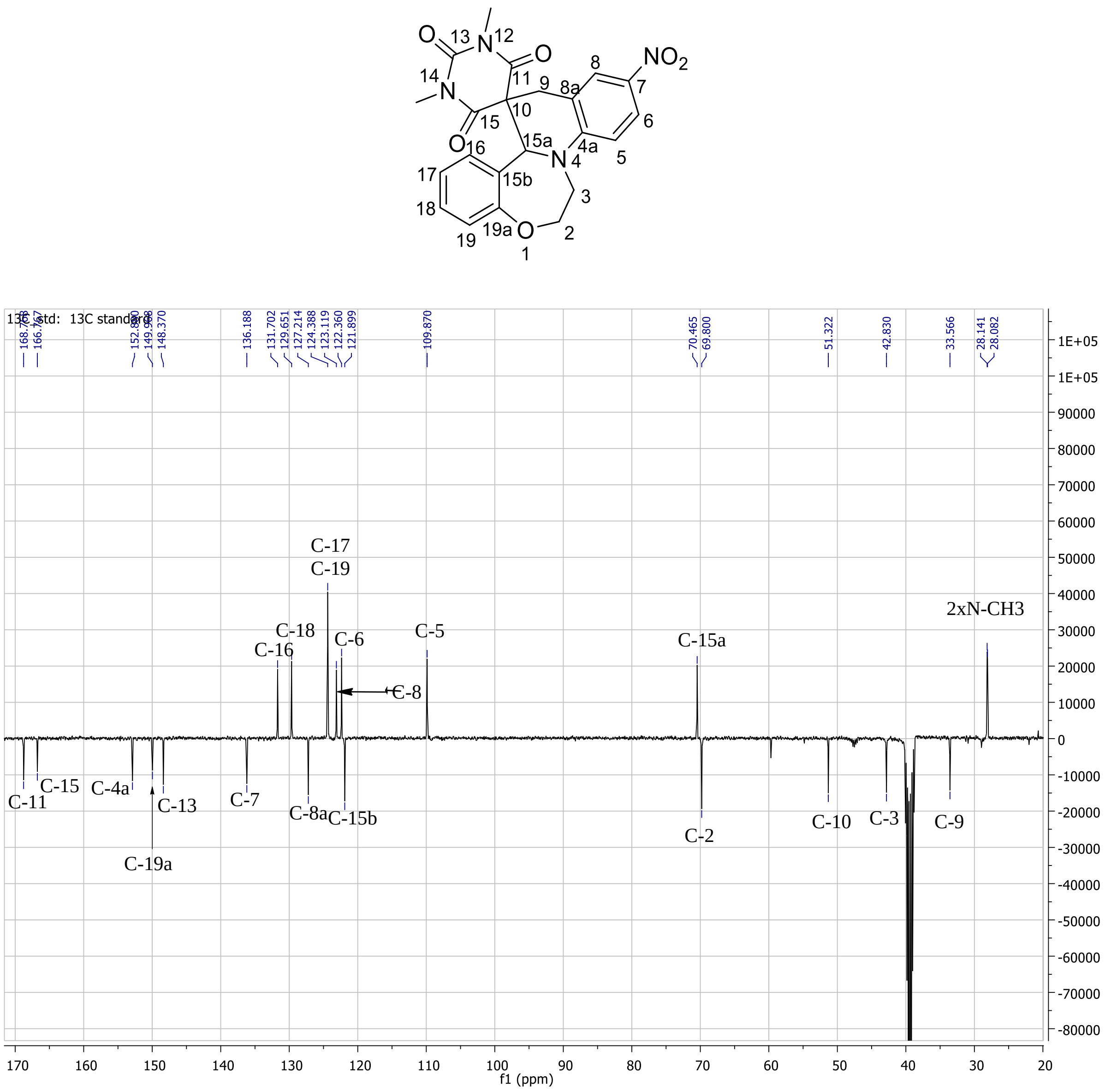

Figure S14. J-modulated ^{13}C-NMR spectrum of *rac*-**10a** measured in $CDCl_3$ (100 MHz).

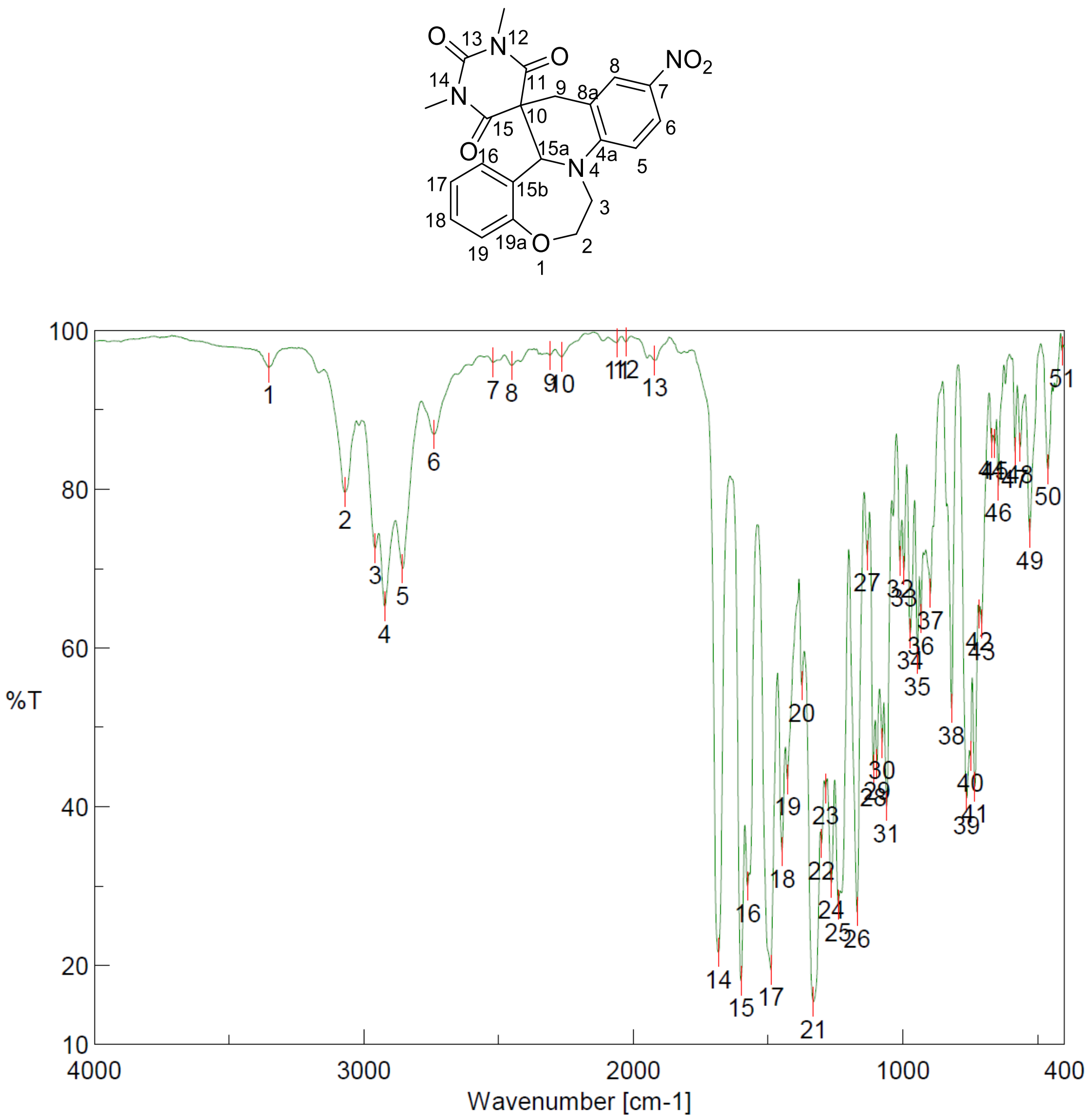

Figure S15. IR spectrum of *rac*-**10a** recorded as KBr disc

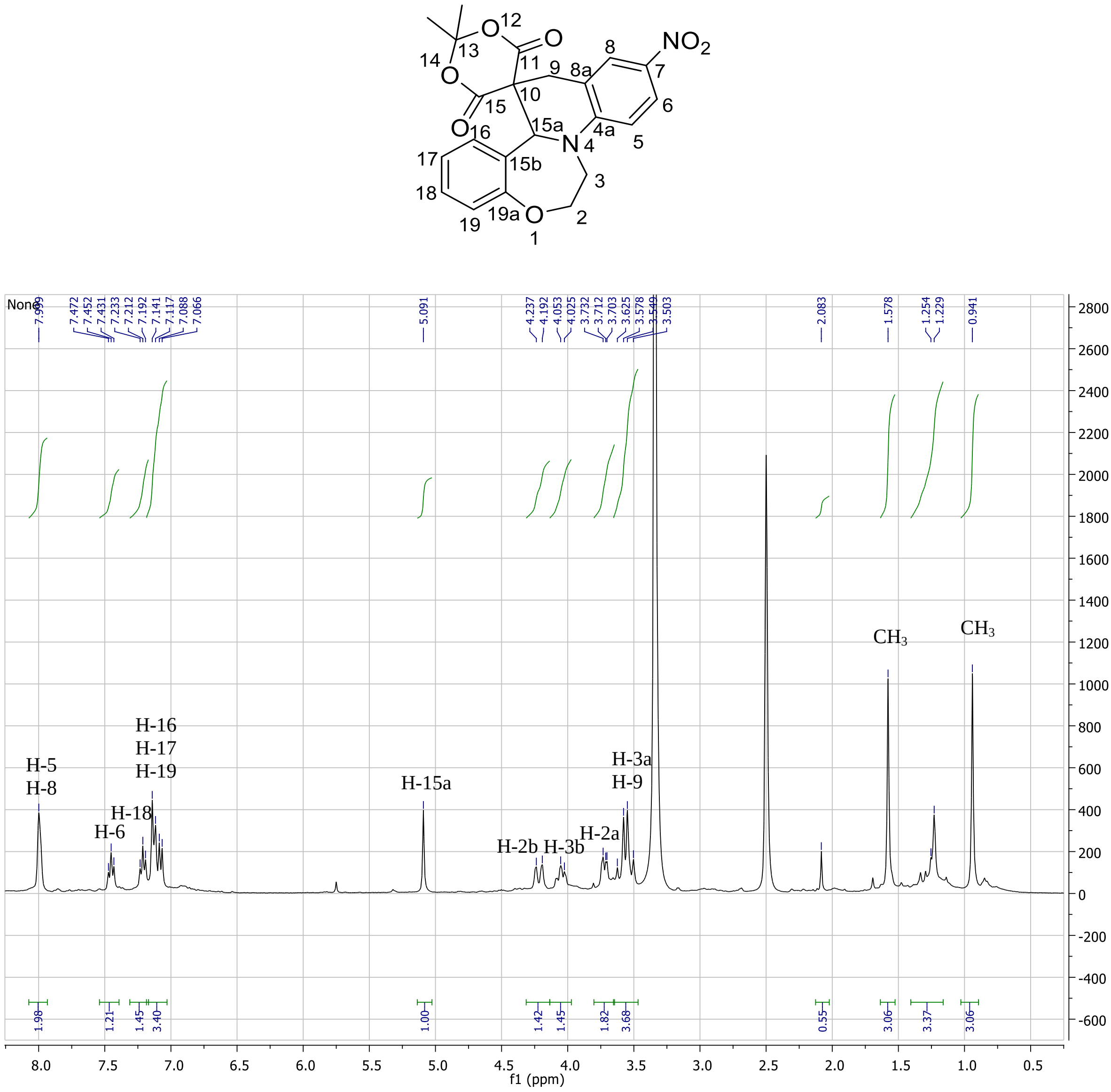

Figure S16. ^{1}H-NMR spectrum of *rac*-**11a** measured in DMSO-d$_6$ (400 MHz)

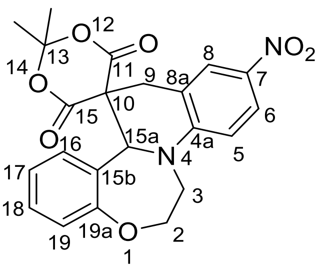

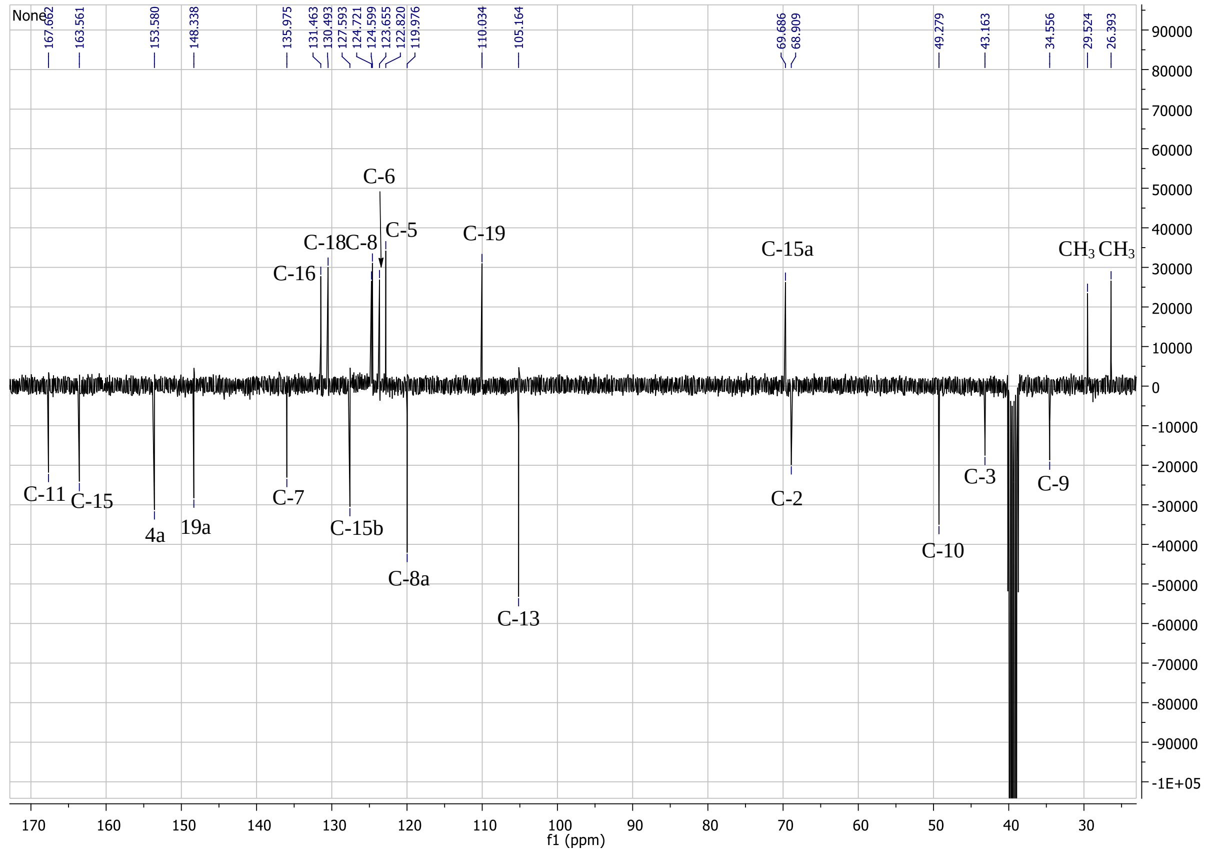

Figure S17. J-modulated ^{13}C-NMR spectrum of *rac*-**11a** measured in DMSO-d$_6$ (100 MHz)

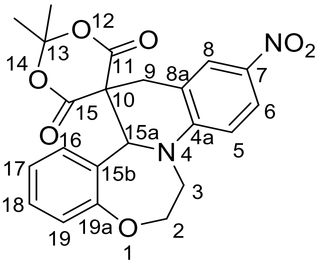

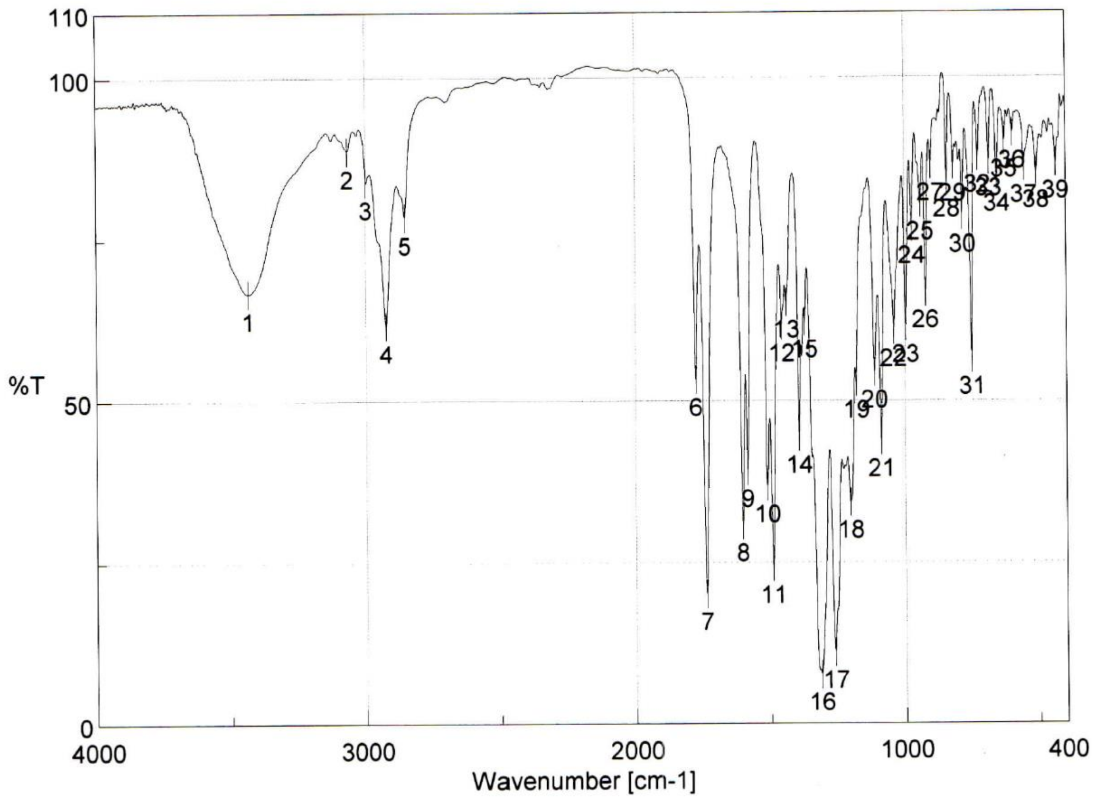

Figure S18. IR spectrum of *rac*-**11a** recorded as KBr disc

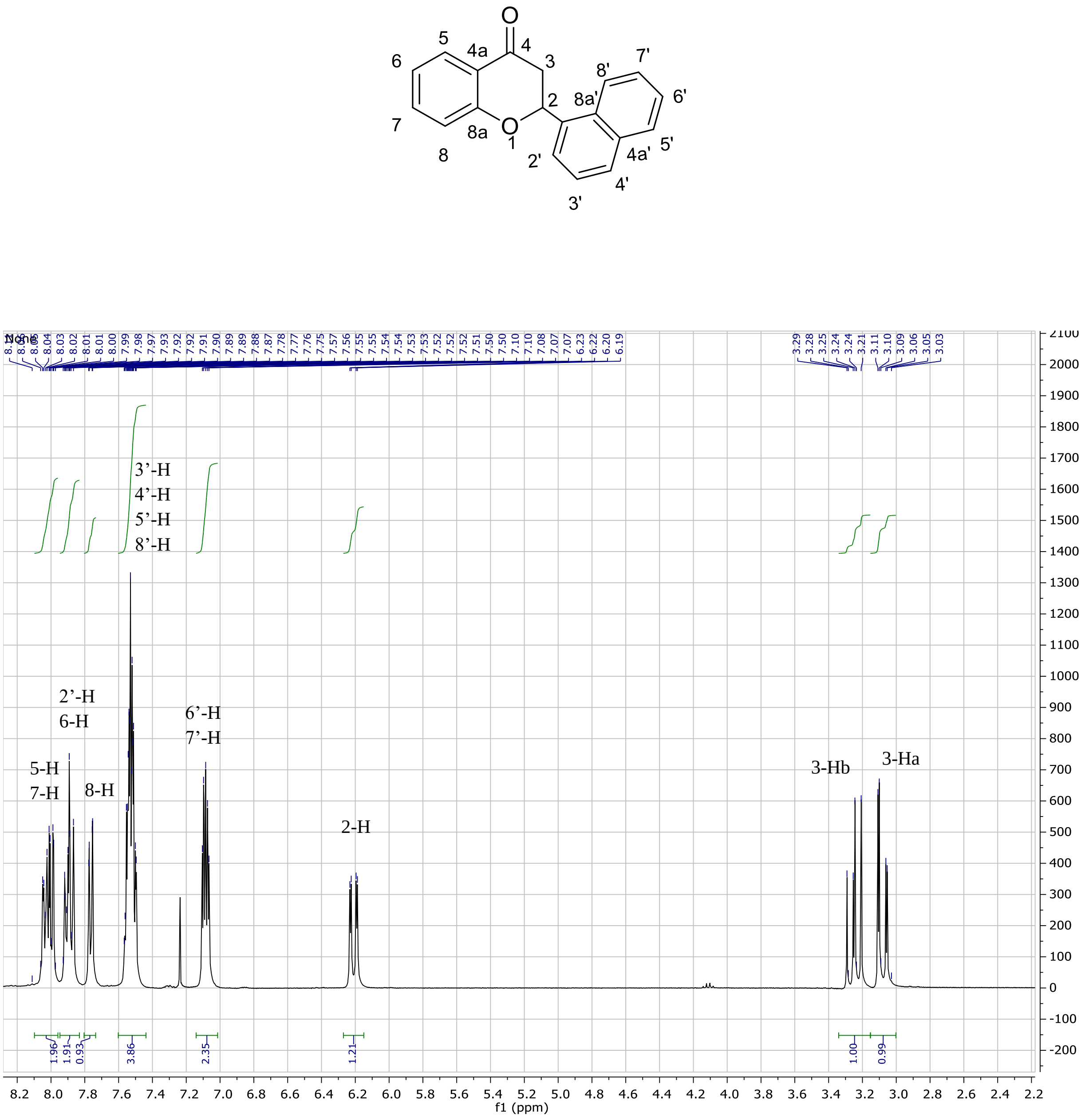

Figure S19. ^{1}H-NMR spectrum of *rac*-**7b** measured in $CDCl_3$ (360 MHz)

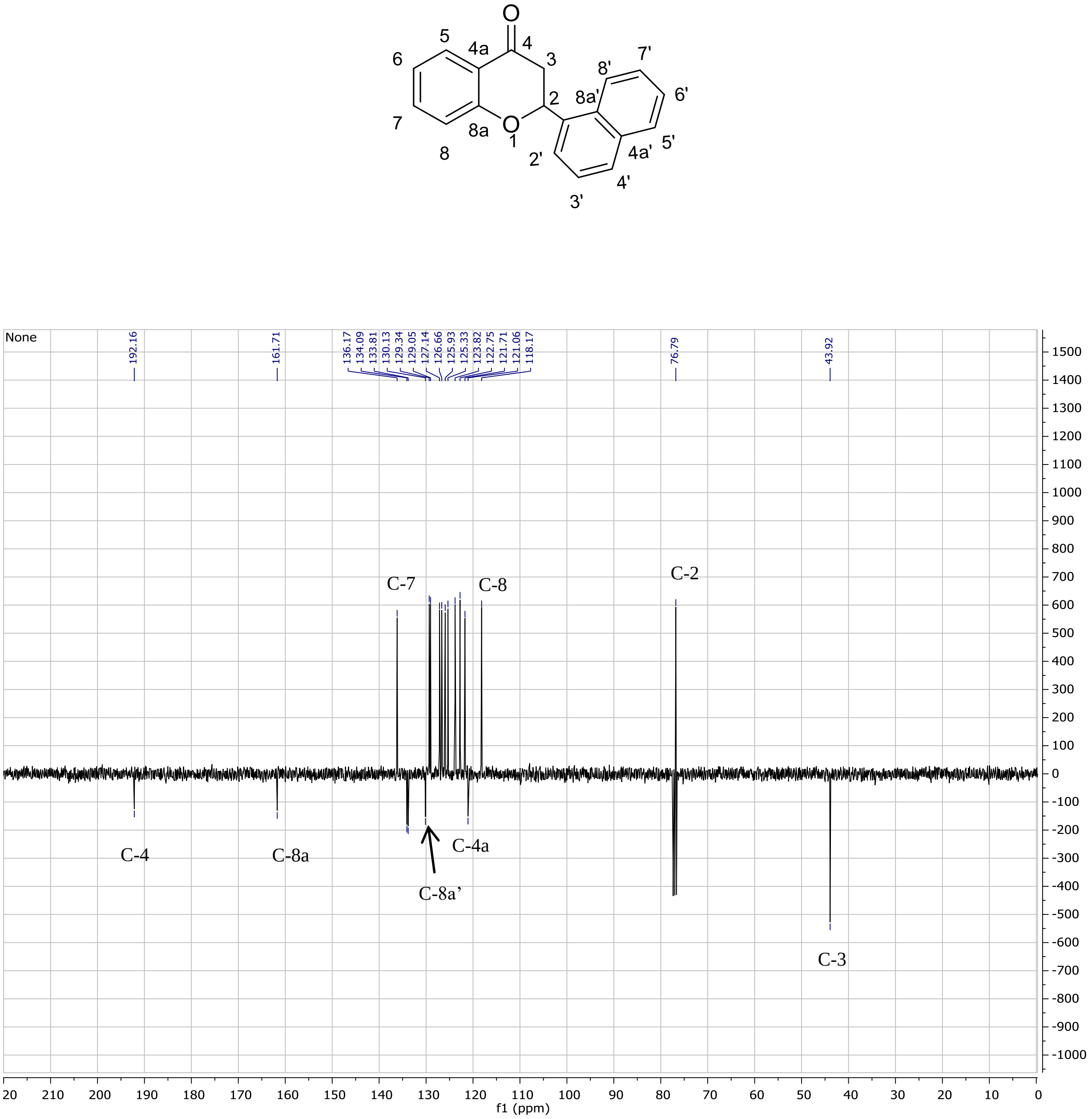

Figure S20. J-modulated ^{13}C-NMR spectrum of *rac*-**7b** measured in $CDCl_3$ (90 MHz)

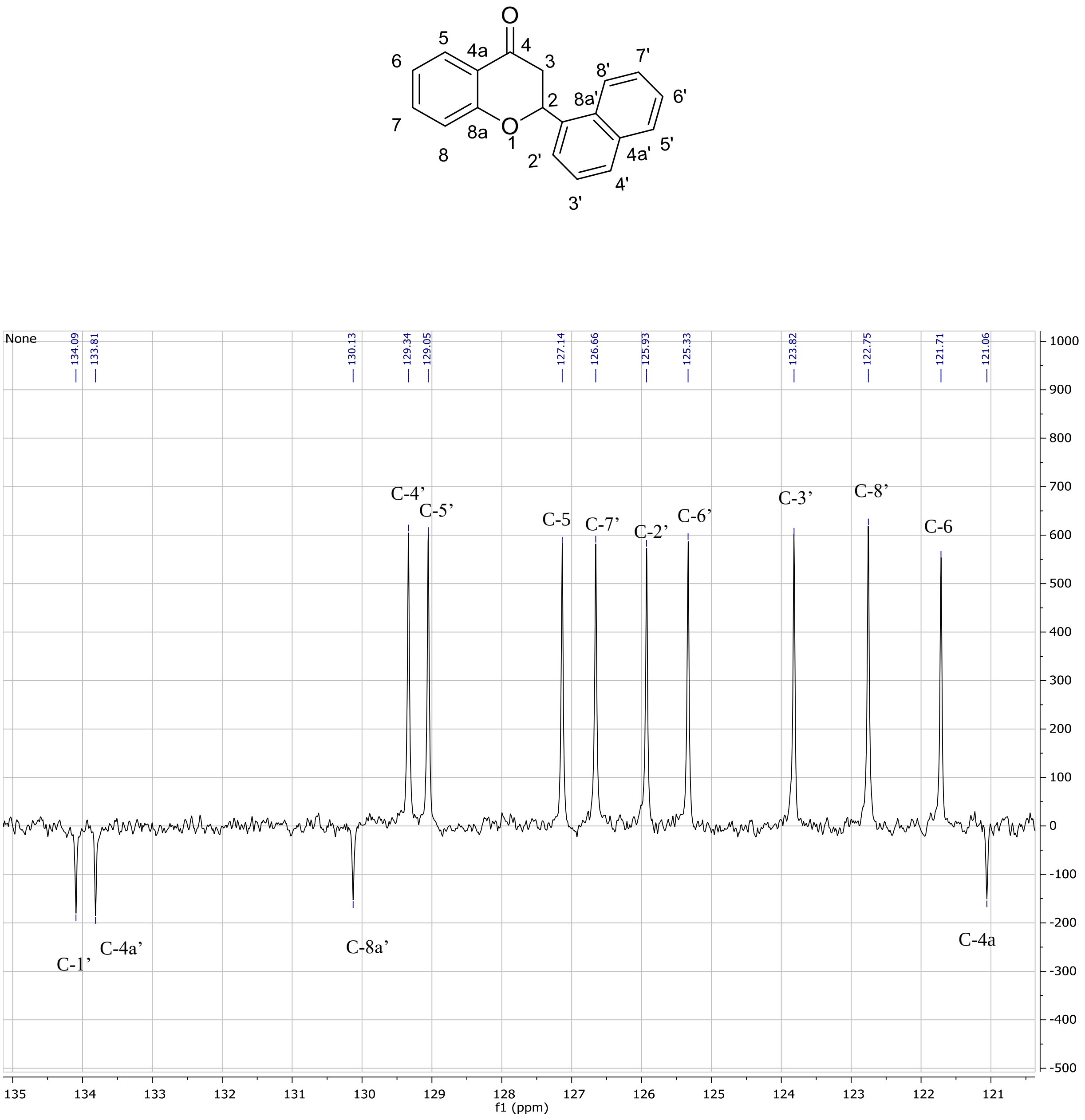

Figure S21. J-modulated ^{13}C-NMR spectrum of *rac*-**7b** measured in $CDCl_3$ (90 MHz)

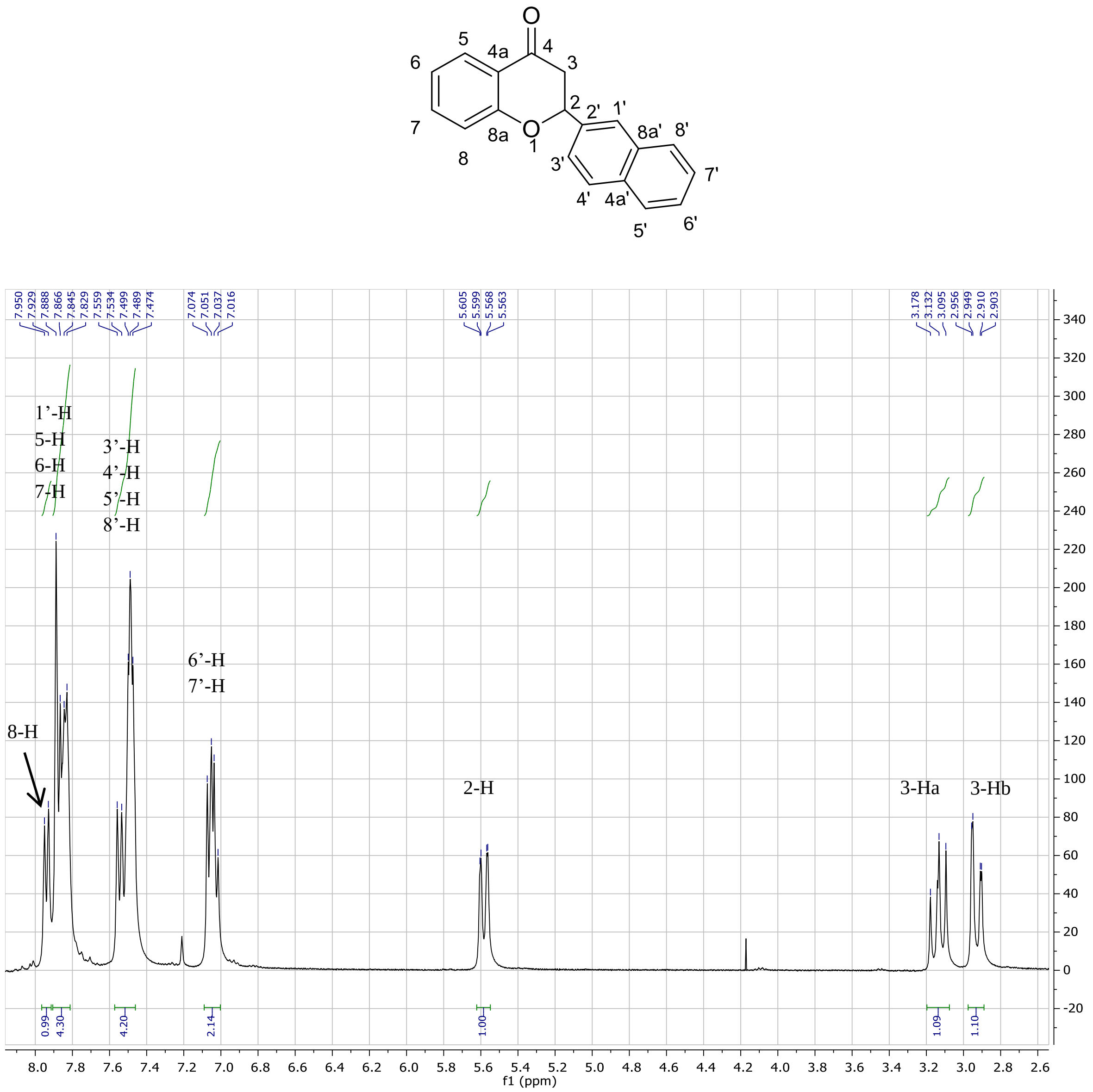

Figure S22. ^{1}H-NMR spectrum of *rac*-**7c** measured in $CDCl_3$ (360 MHz)

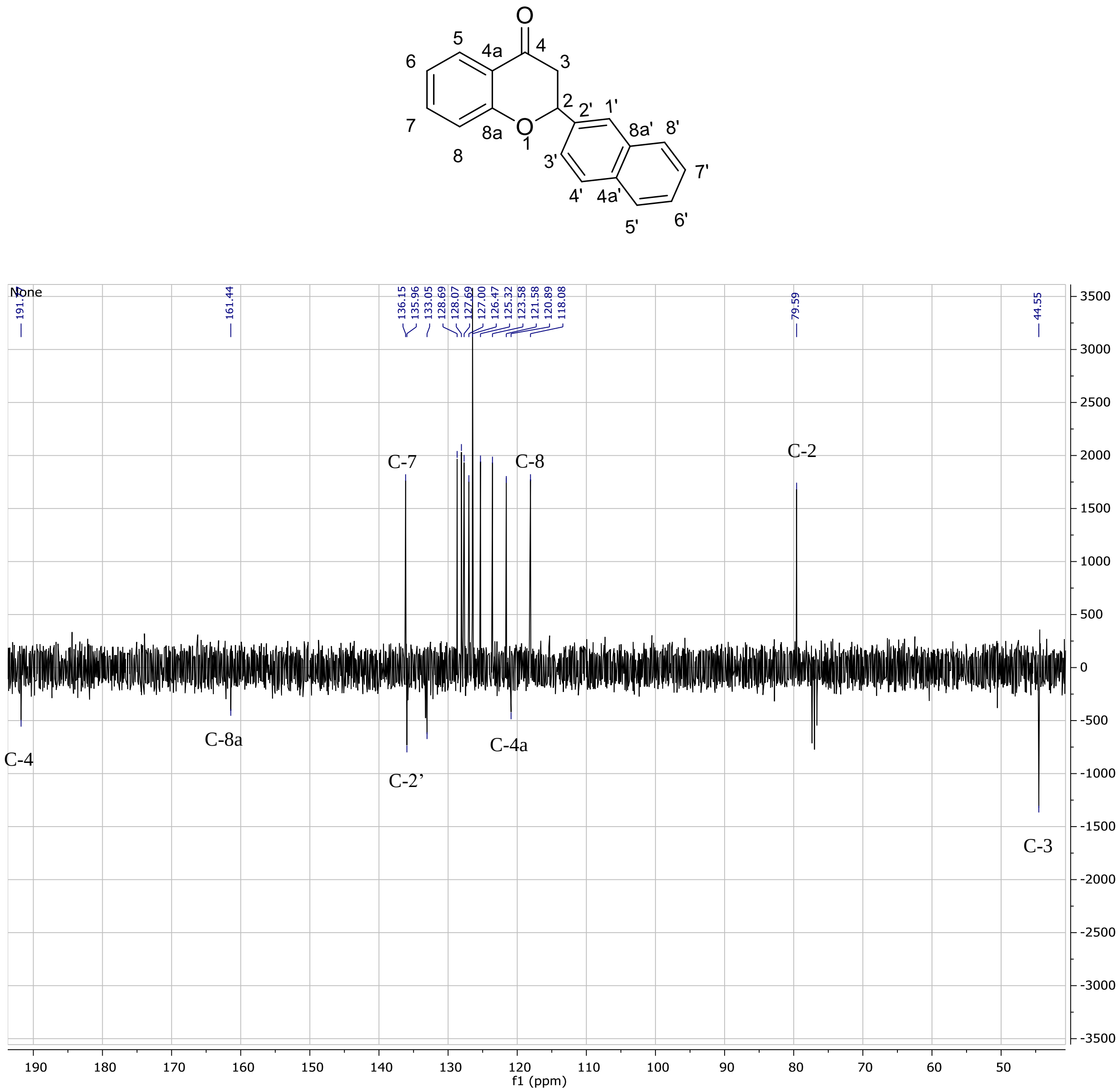

Figure S23. J-modulated ^{13}C-NMR spectrum of *rac*-**7c** measured in $CDCl_3$ (90 MHz)

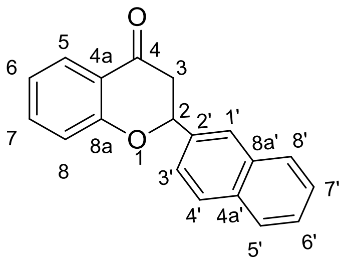

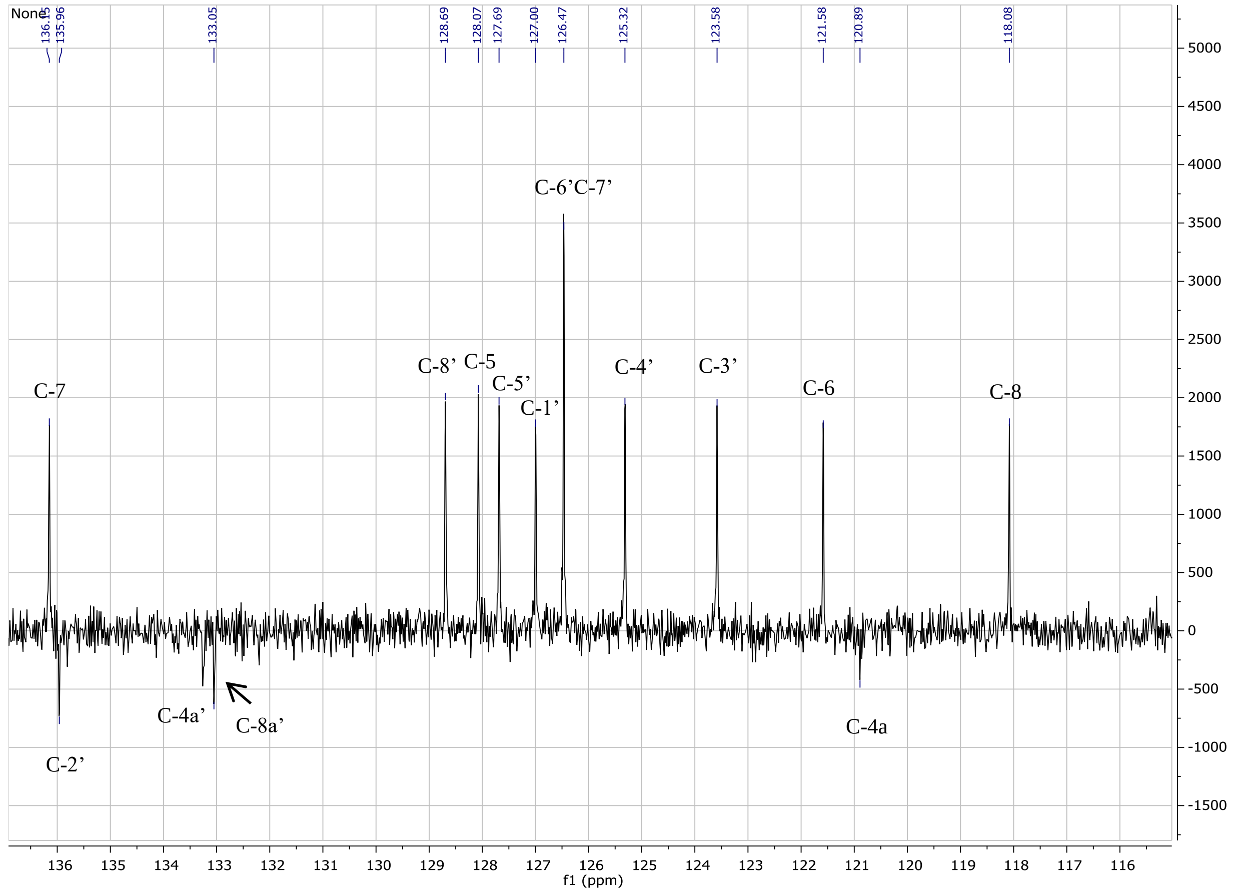

Figure S24. J-modulated ^{13}C-NMR spectrum of *rac*-**7c** measured in $CDCl_3$ (90 MHz)

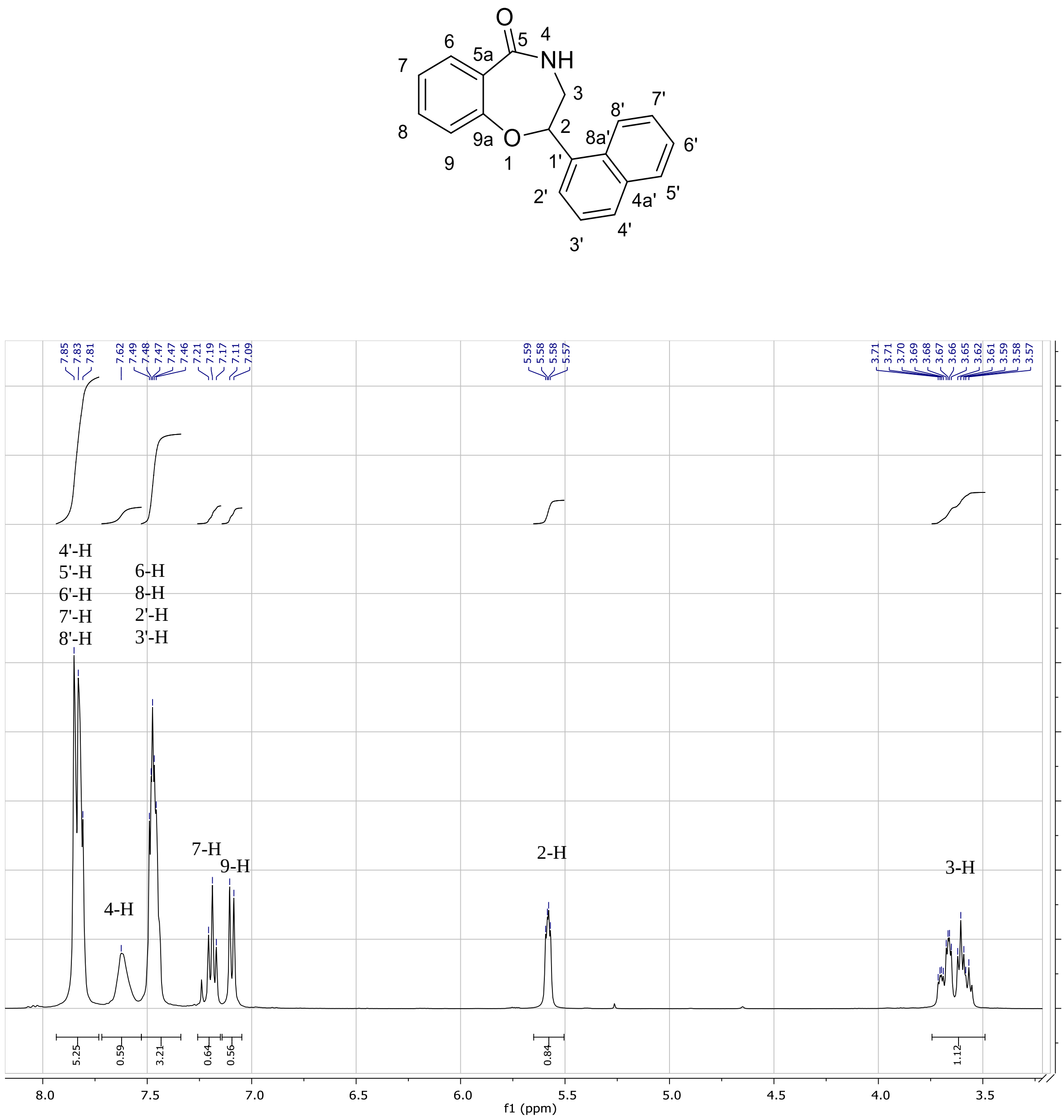

Figure S25. ^{1}H-NMR spectrum of *rac*-**8b** measured in $CDCl_3$ (400 MHz)

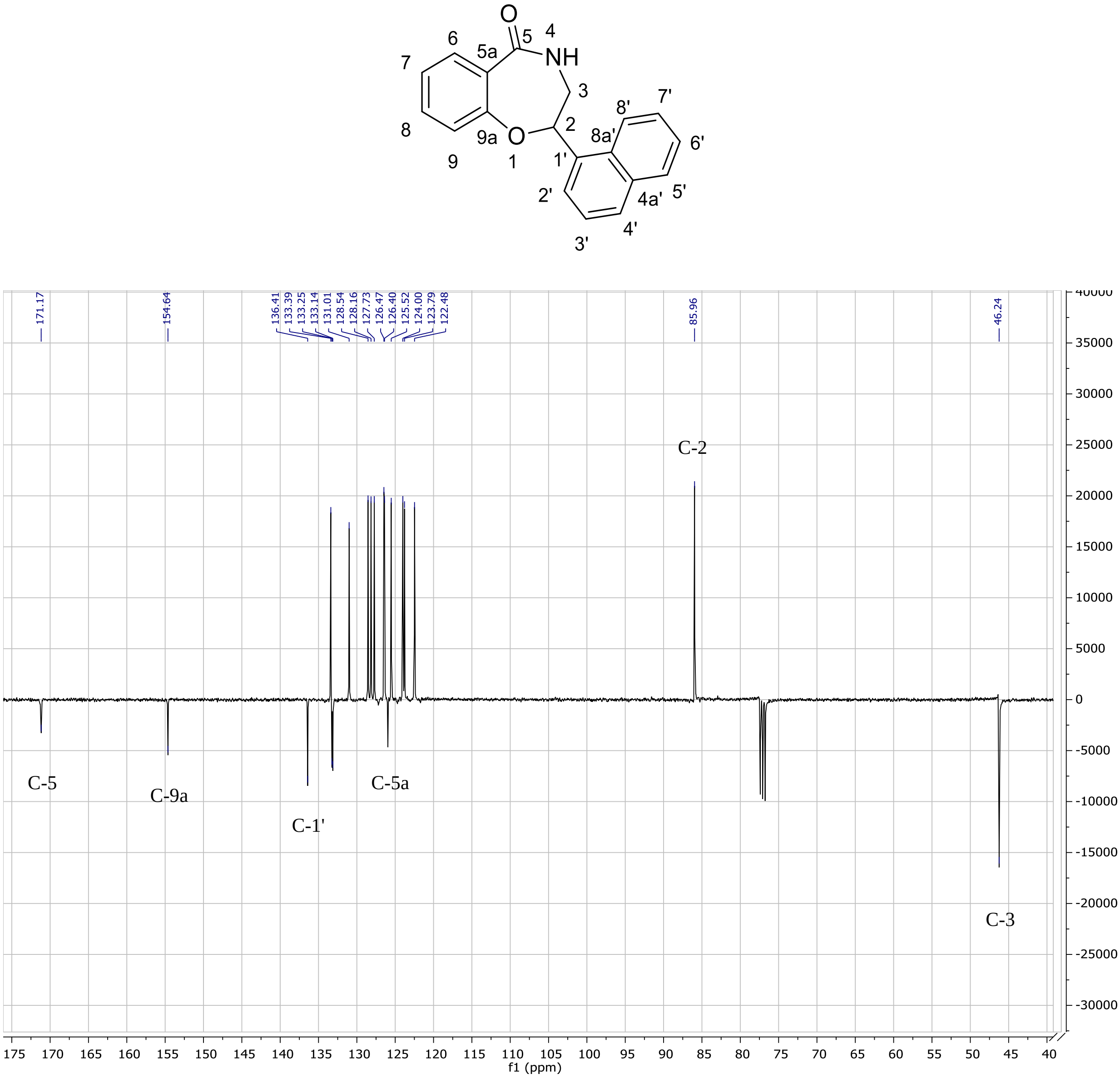

Figure S26. J-modulated ^{13}C-NMR spectrum of *rac*-**8b** measured in $CDCl_3$ (100 MHz)

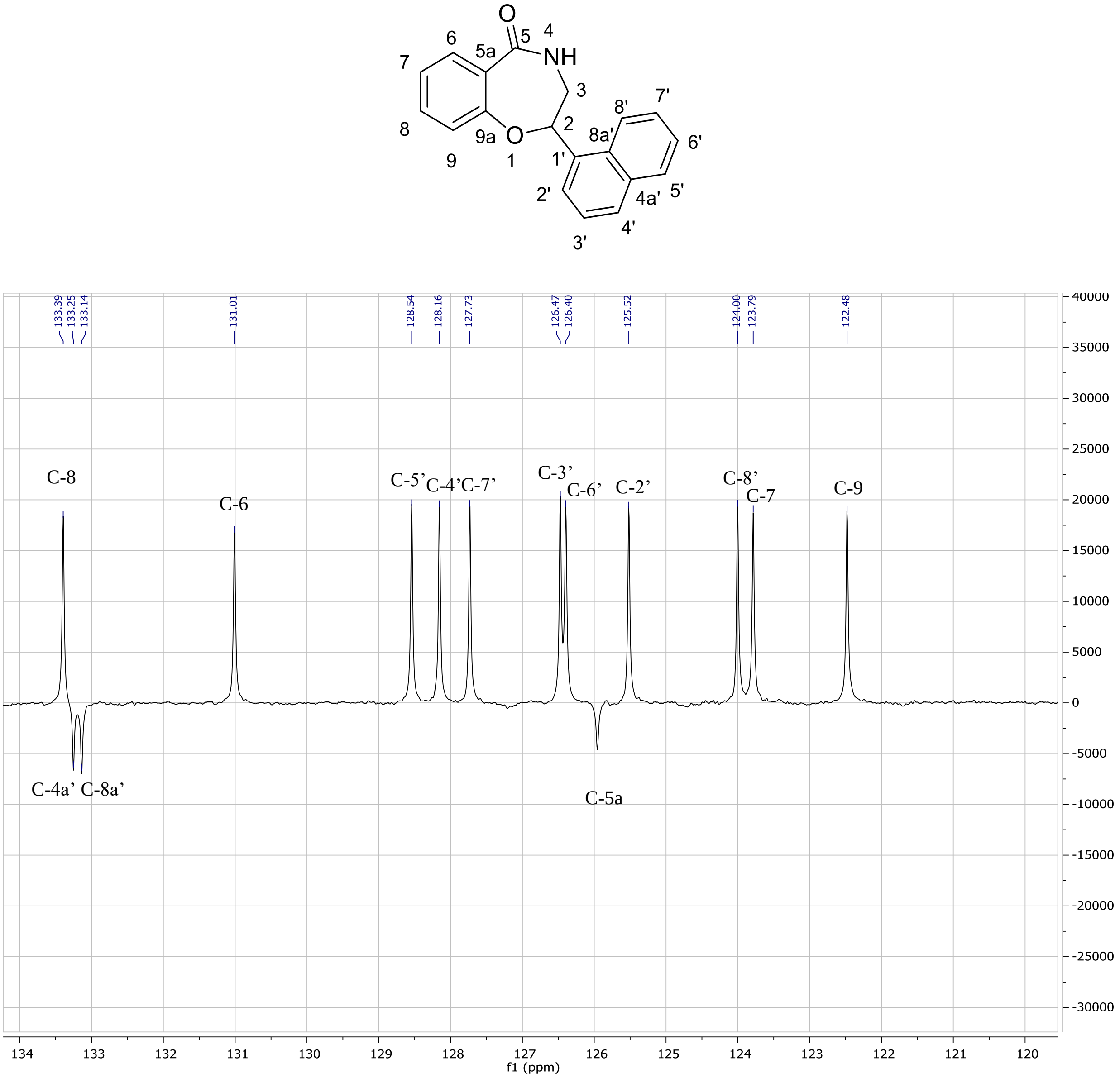

Figure S27. J-modulated ^{13}C-NMR spectra of *rac*-**8b** measured in $CDCl_3$ (100 MHz)

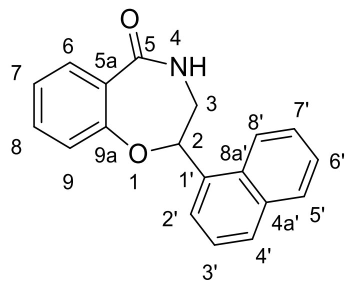

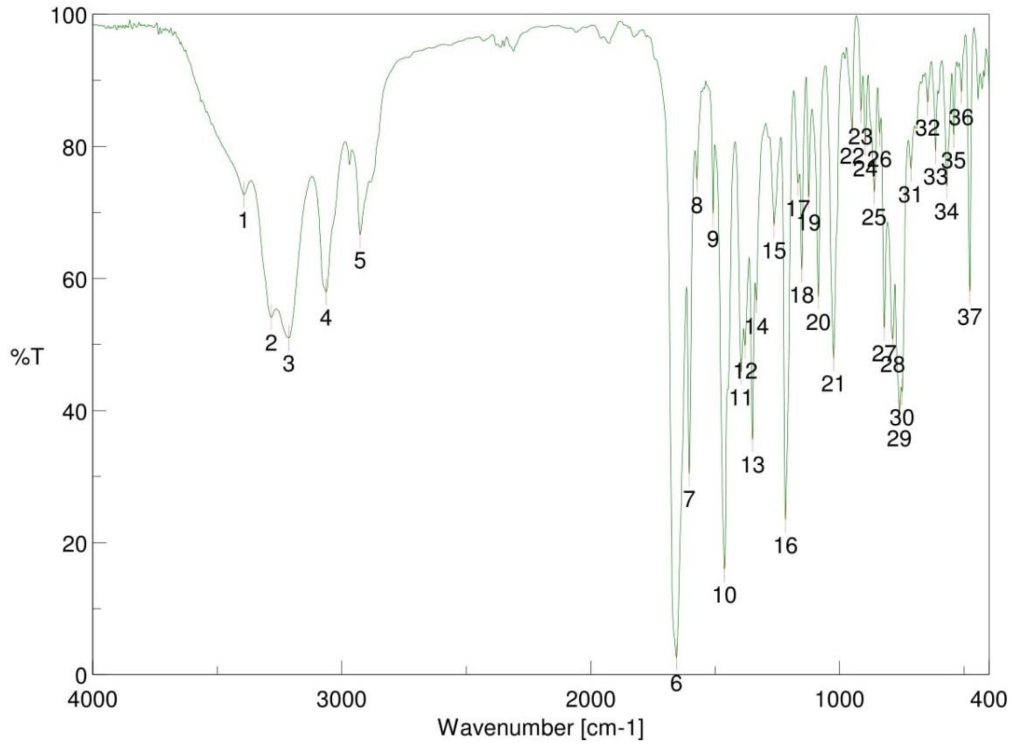

Figure S28 IR spectra of *rac*-**8b** recorded as KBr disc

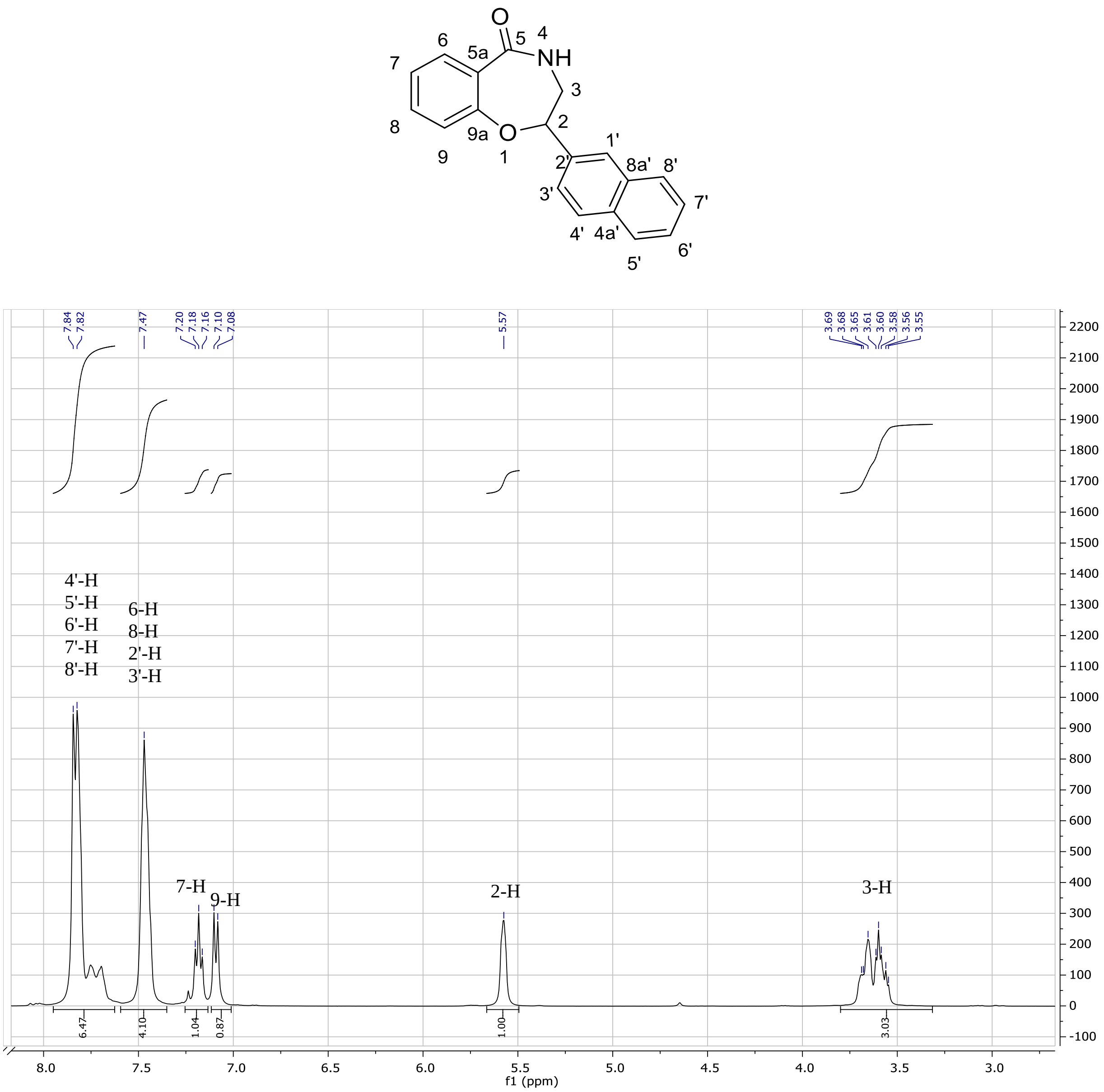

Figure S29. ^{1}H-NMR spectrum of *rac*-**8c** measured in $CDCl_3$ (400 MHz)

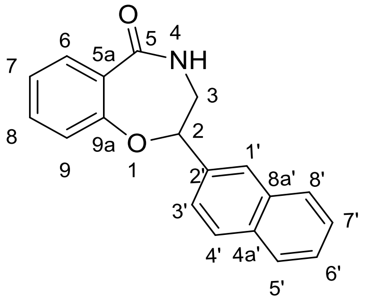

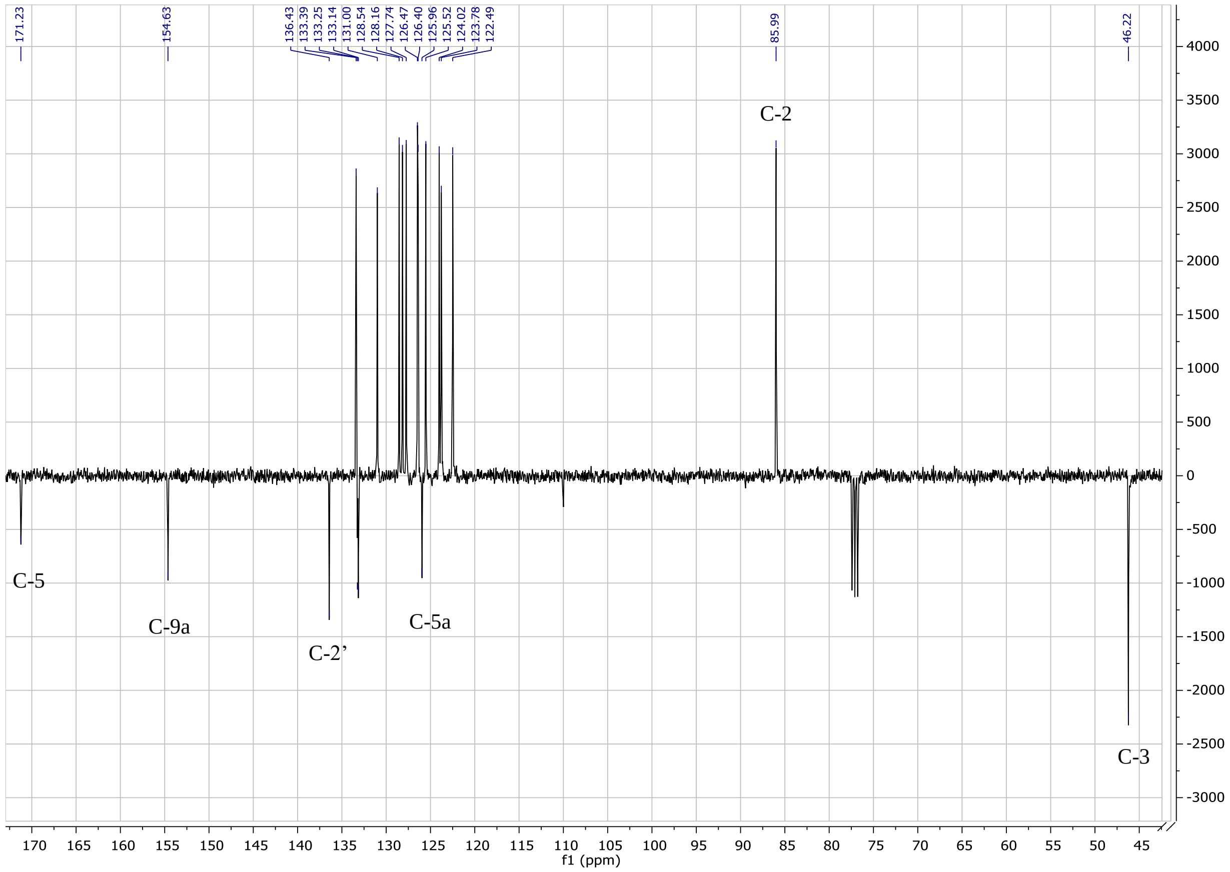

Figure S30. J-modulated ^{13}C-NMR spectrum of *rac*-**8c** measured in $CDCl_3$ (100 MHz)

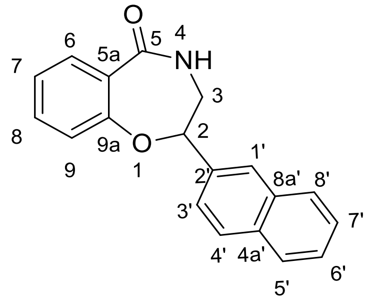

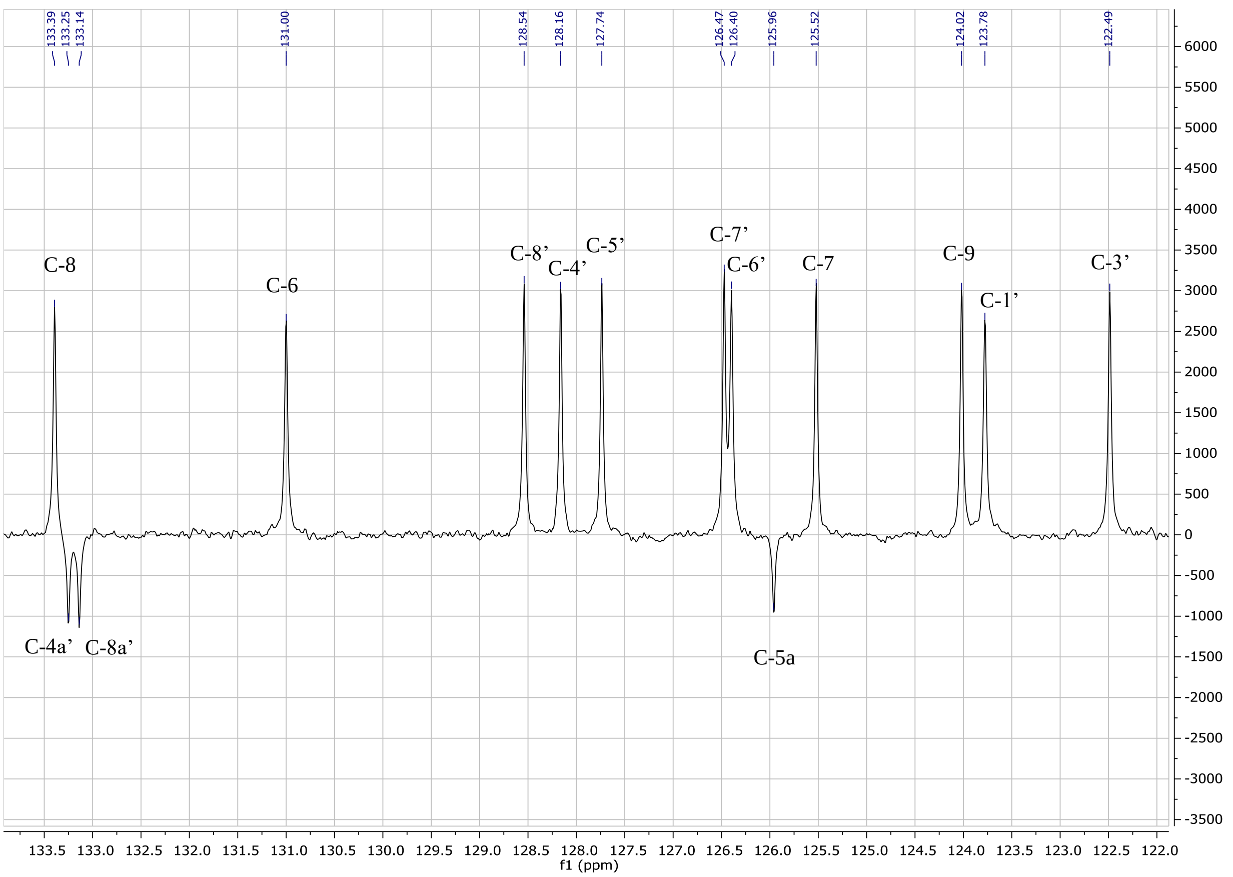

Figure S31 J-modulated ^{13}C-NMR spectrum of *rac*-**8c** measured in $CDCl_3$ (100 MHz)

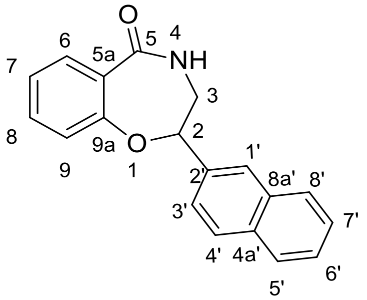

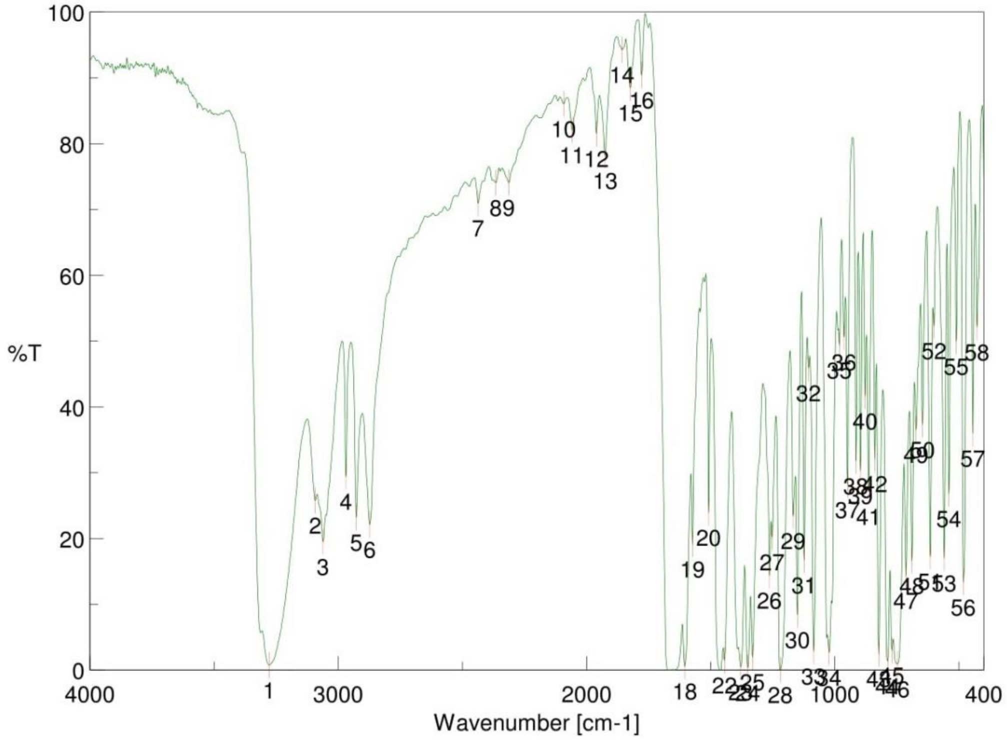

Figure S32 IR spectrum of *rac*-**8c** recorded as KBr disc

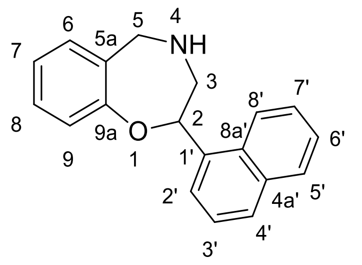

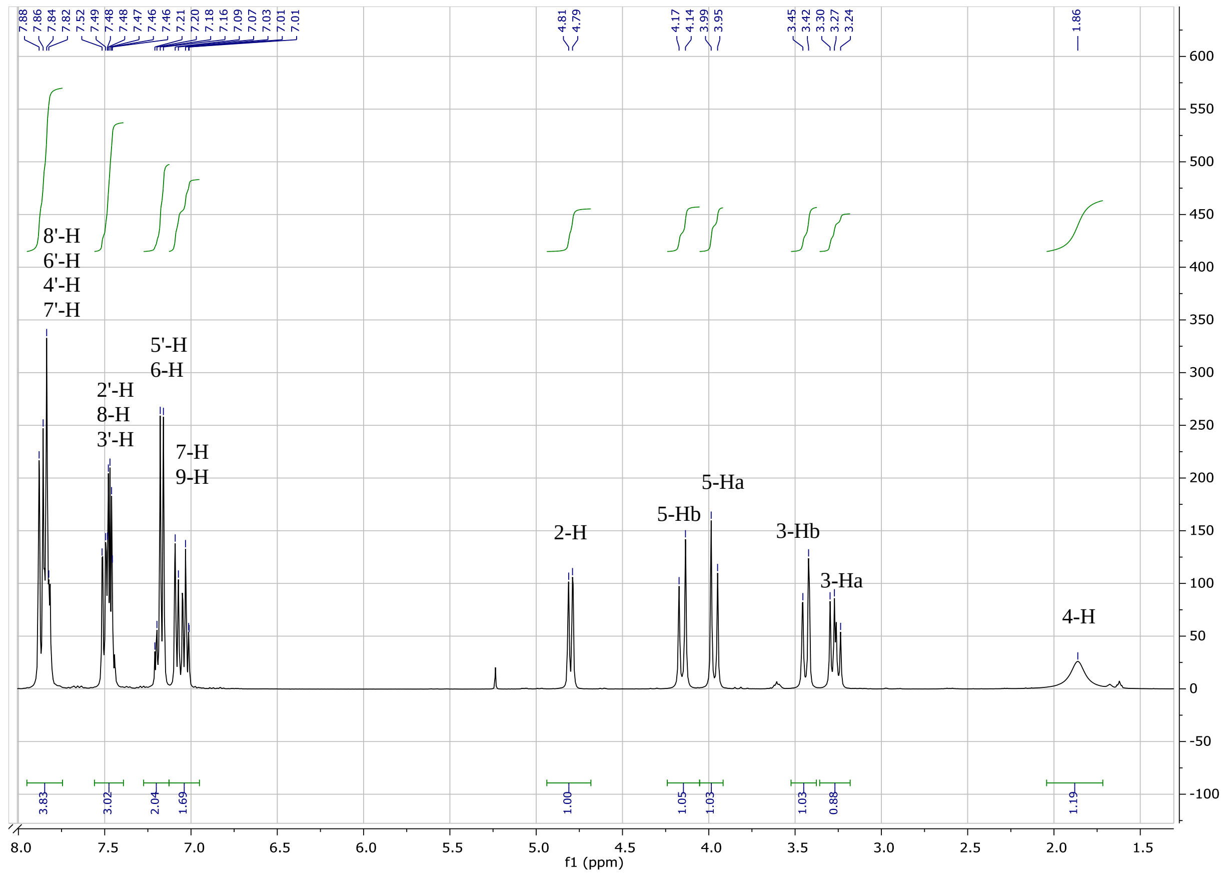

Figure S33. ^{1}H-NMR spectrum of *rac*-**9b** measured in $CDCl_3$ (400 MHz)

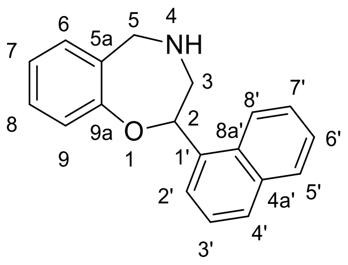

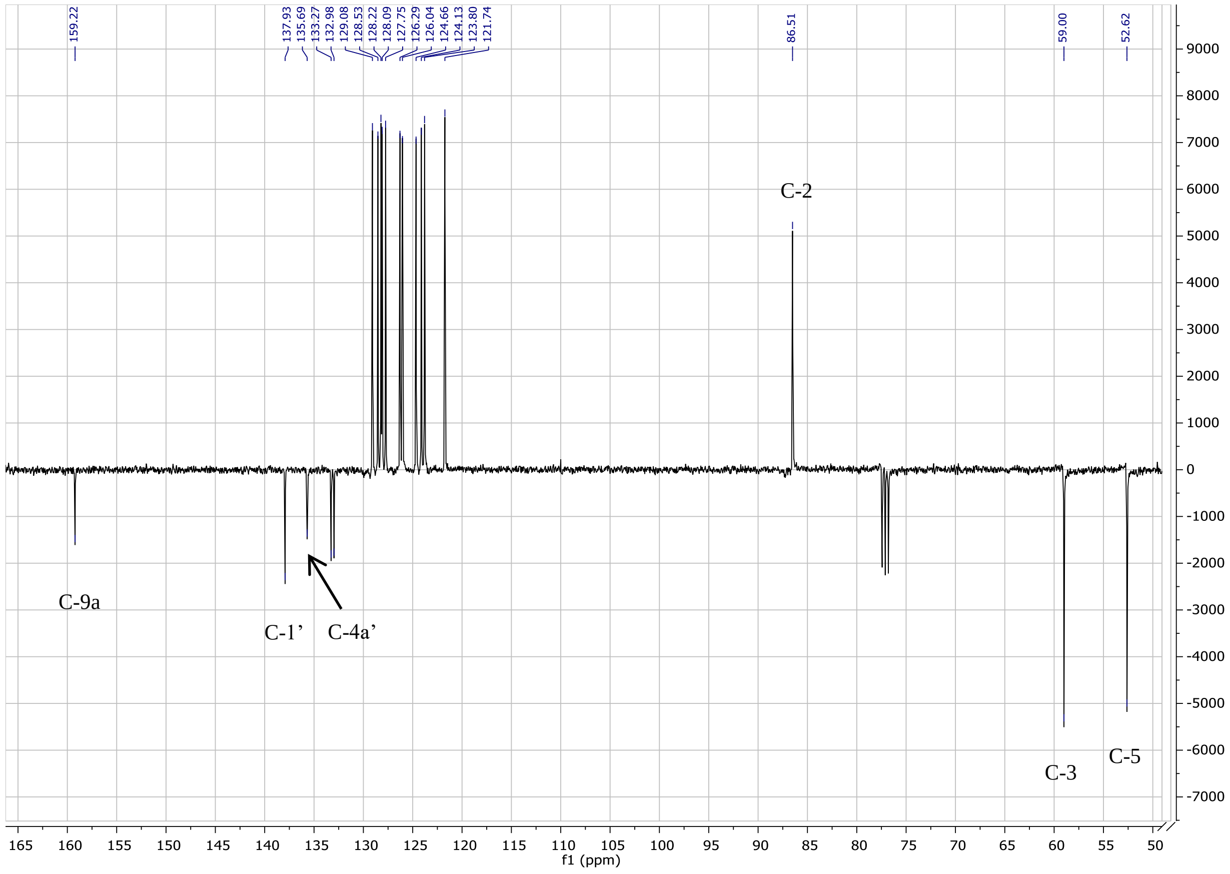

Figure S34. J-modulated ^{13}C-NMR spectrum of *rac*-**9b** measured in $CDCl_3$ (100 MHz)

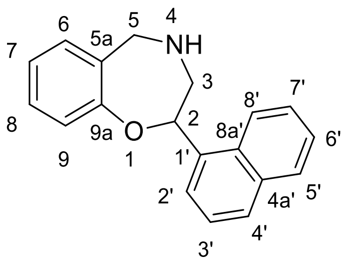

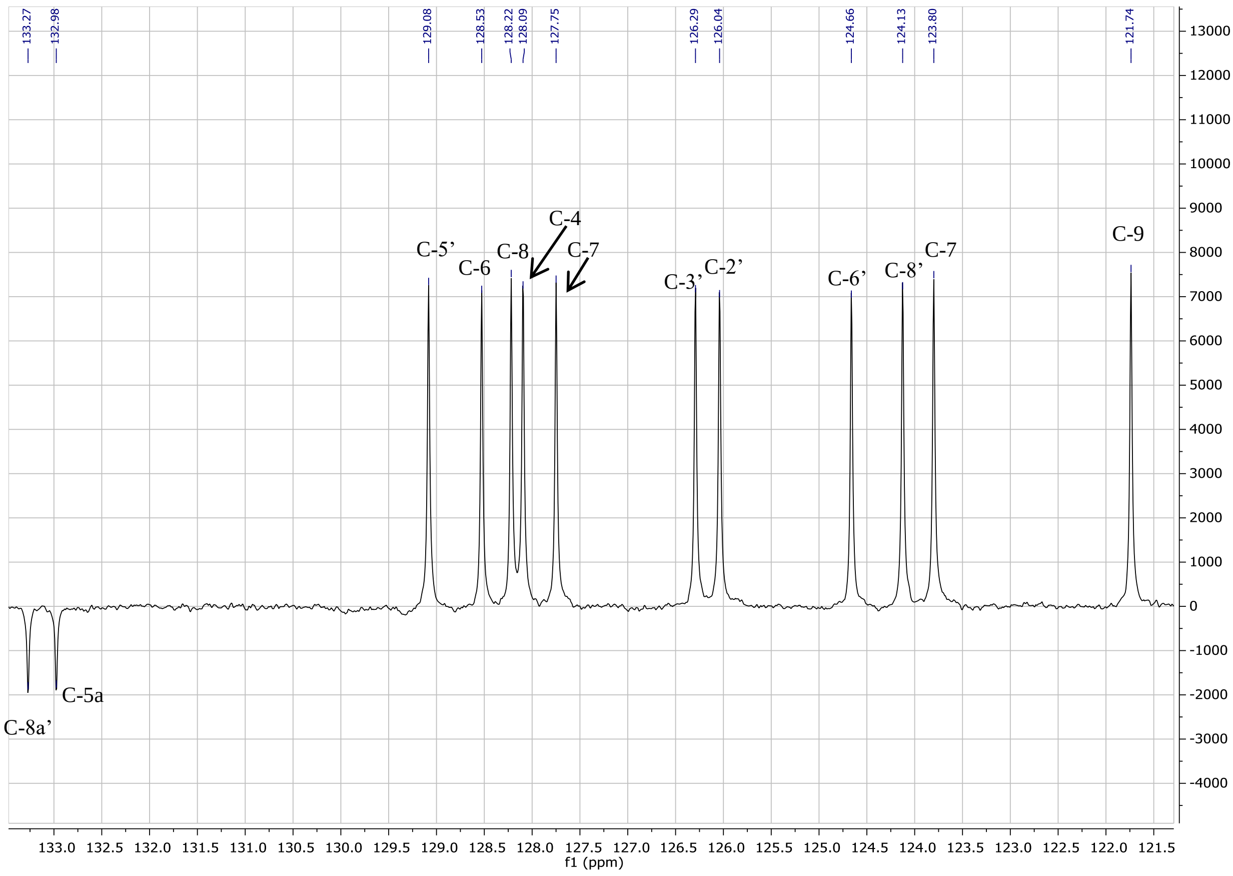

Figure S35. J-modulated ^{13}C-NMR spectrum of *rac*-**9b** measured in $CDCl_3$ (100 MHz)

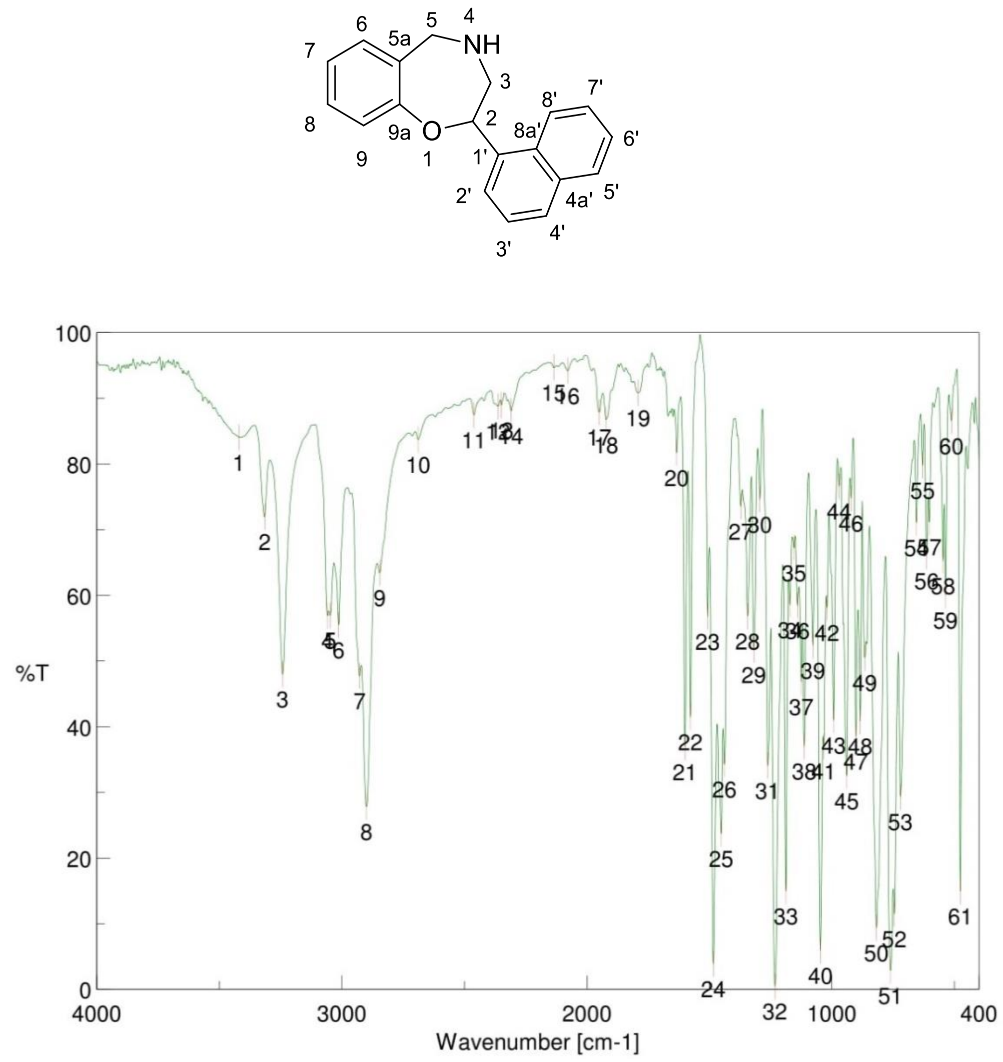

Figure S36. IR spectrum of *rac*-**9b** recorded as KBr disc

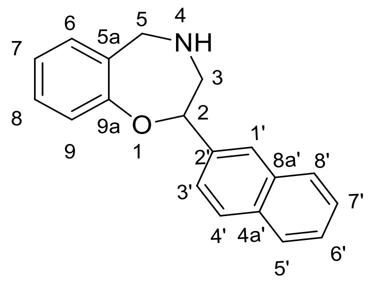

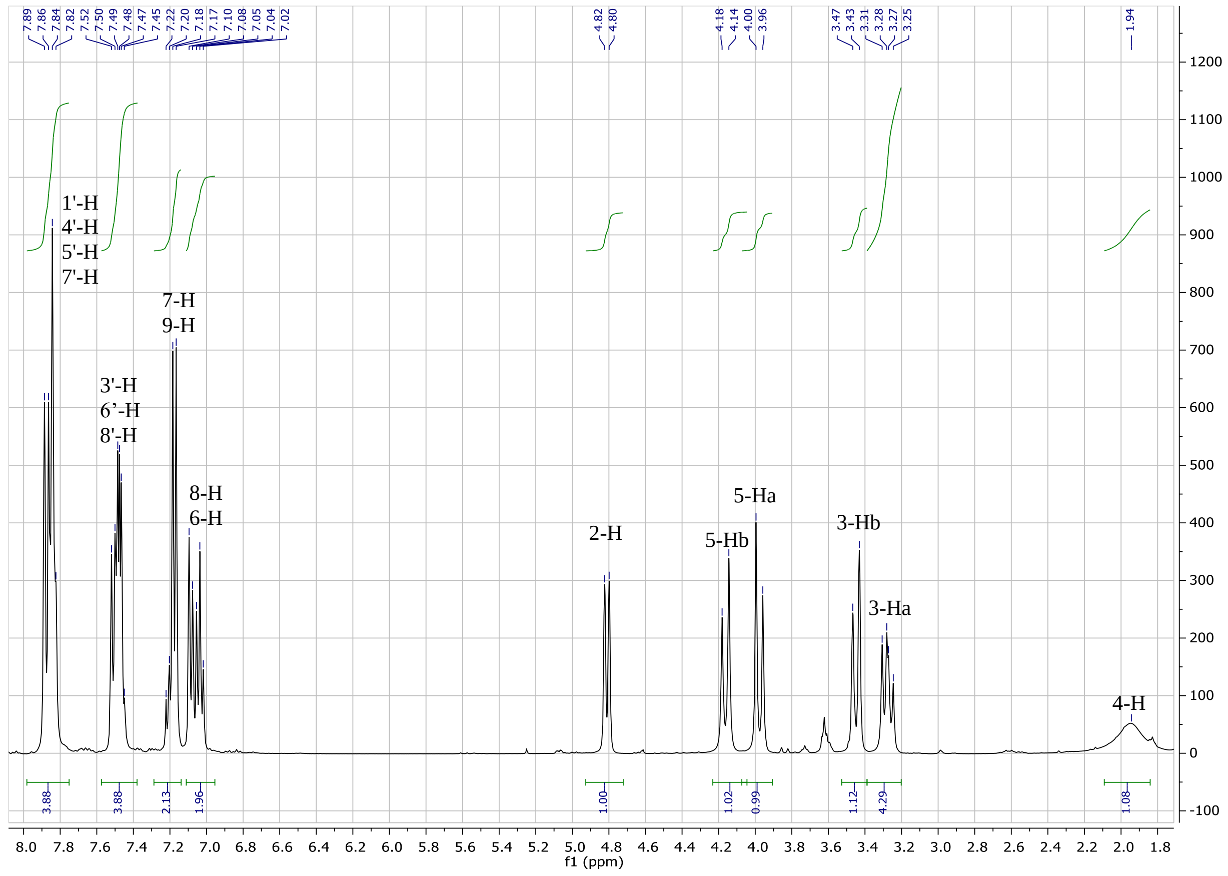

Figure S37. ^{1}H-NMR spectrum of *rac*-**9c** measured in $CDCl_3$ (400 MHz)

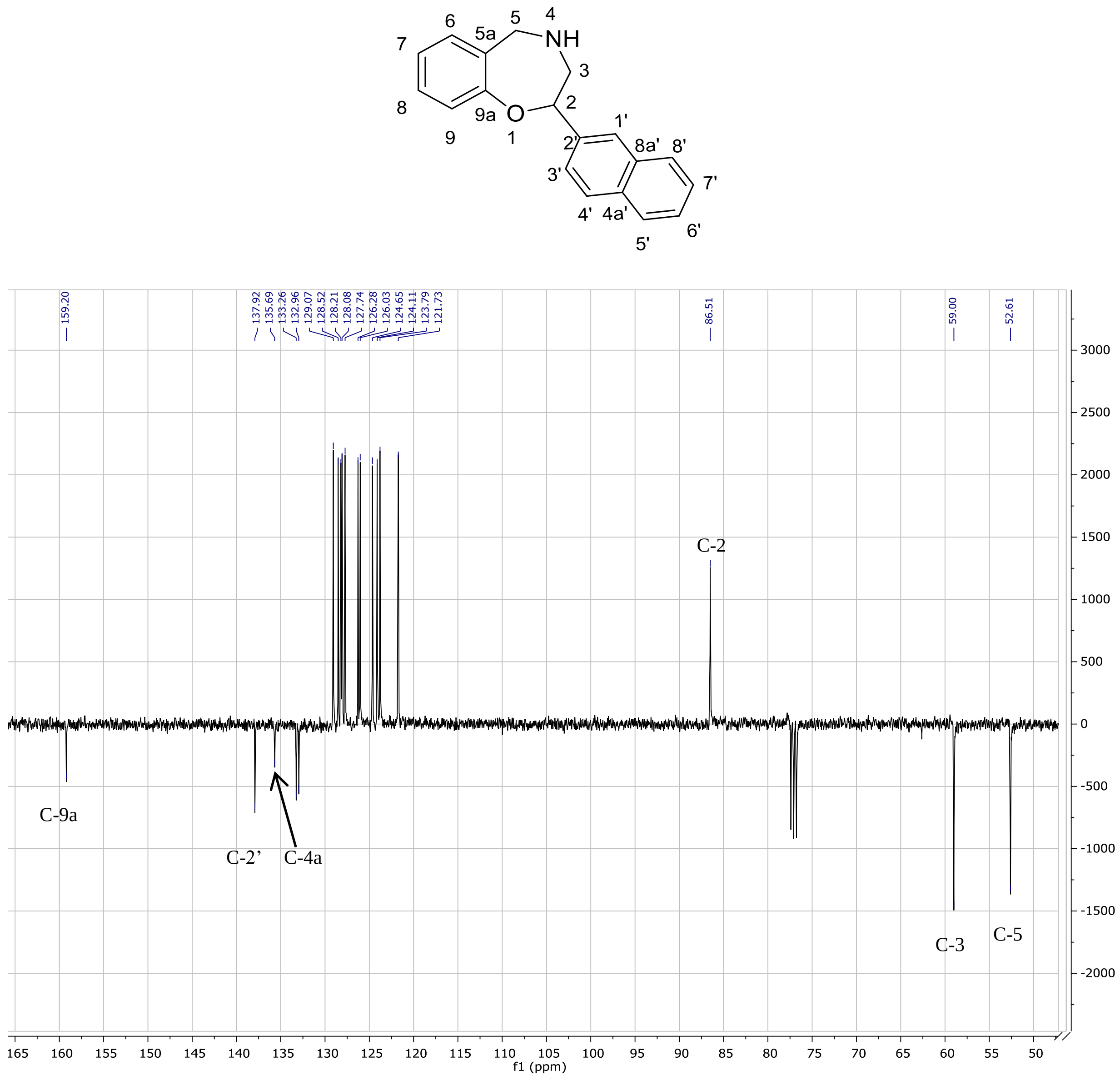

Figure S38. J-modulated ^{13}C-NMR spectrum of *rac*-**9c** measured in $CDCl_3$ (100 MHz)

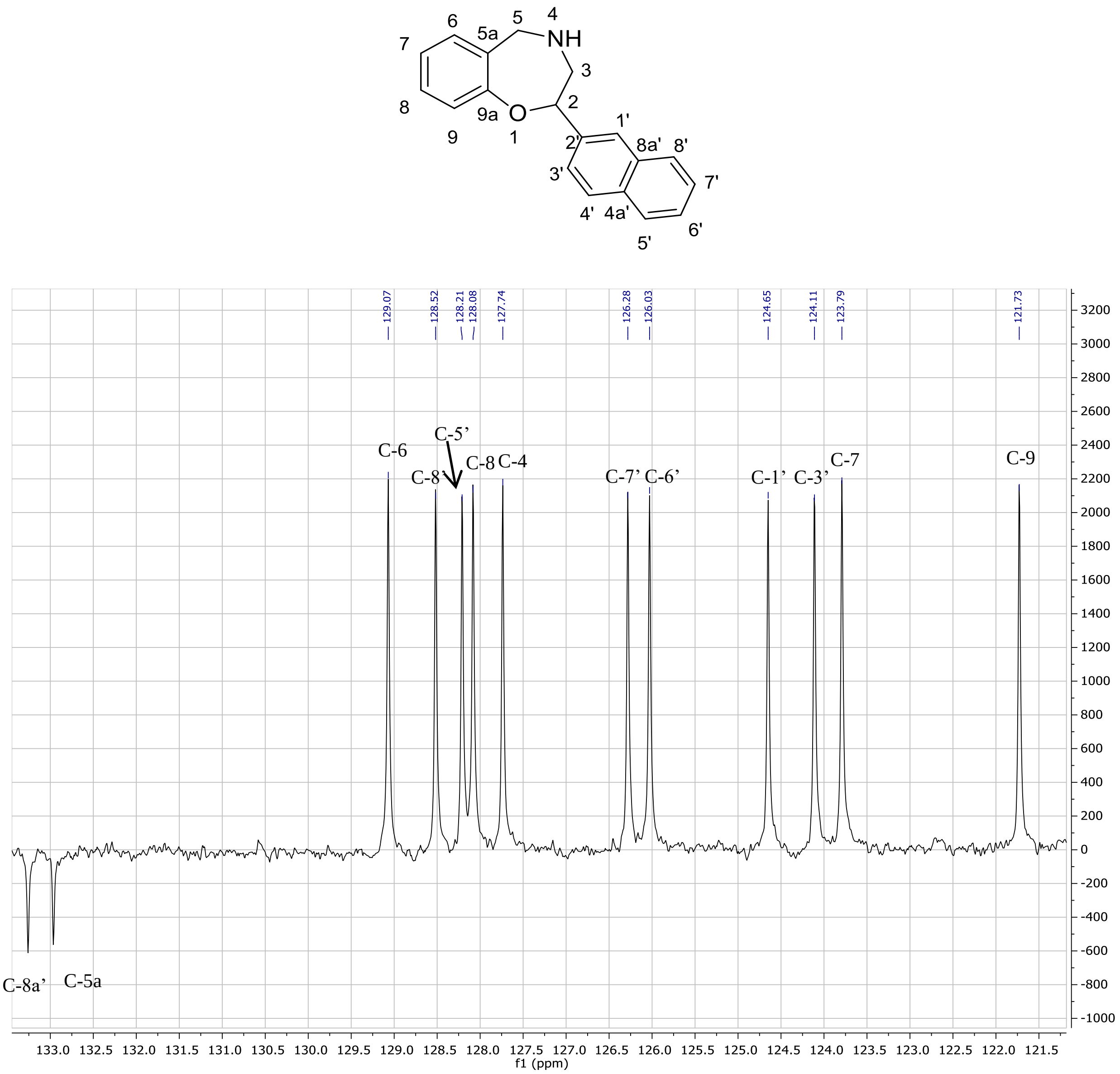

Figure S39. J-modulated ^{13}C-NMR spectrum of *rac*-**9c** measured in $CDCl_3$ (100 MHz)

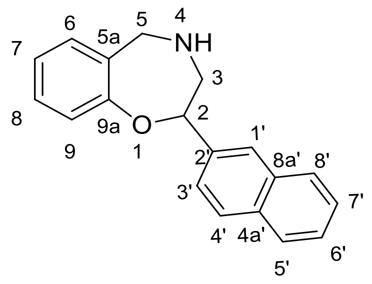

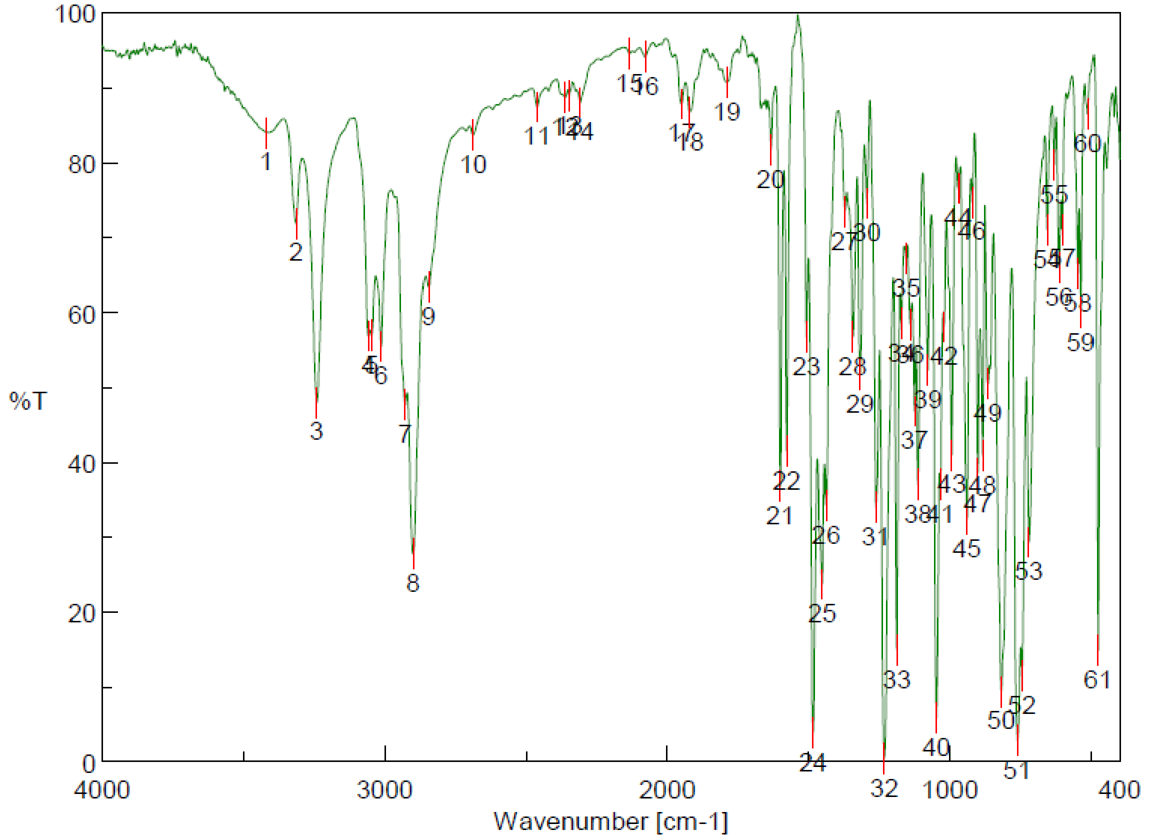

Figure S40. IR spectrum of *rac*-**9c** recorded as KBr disc

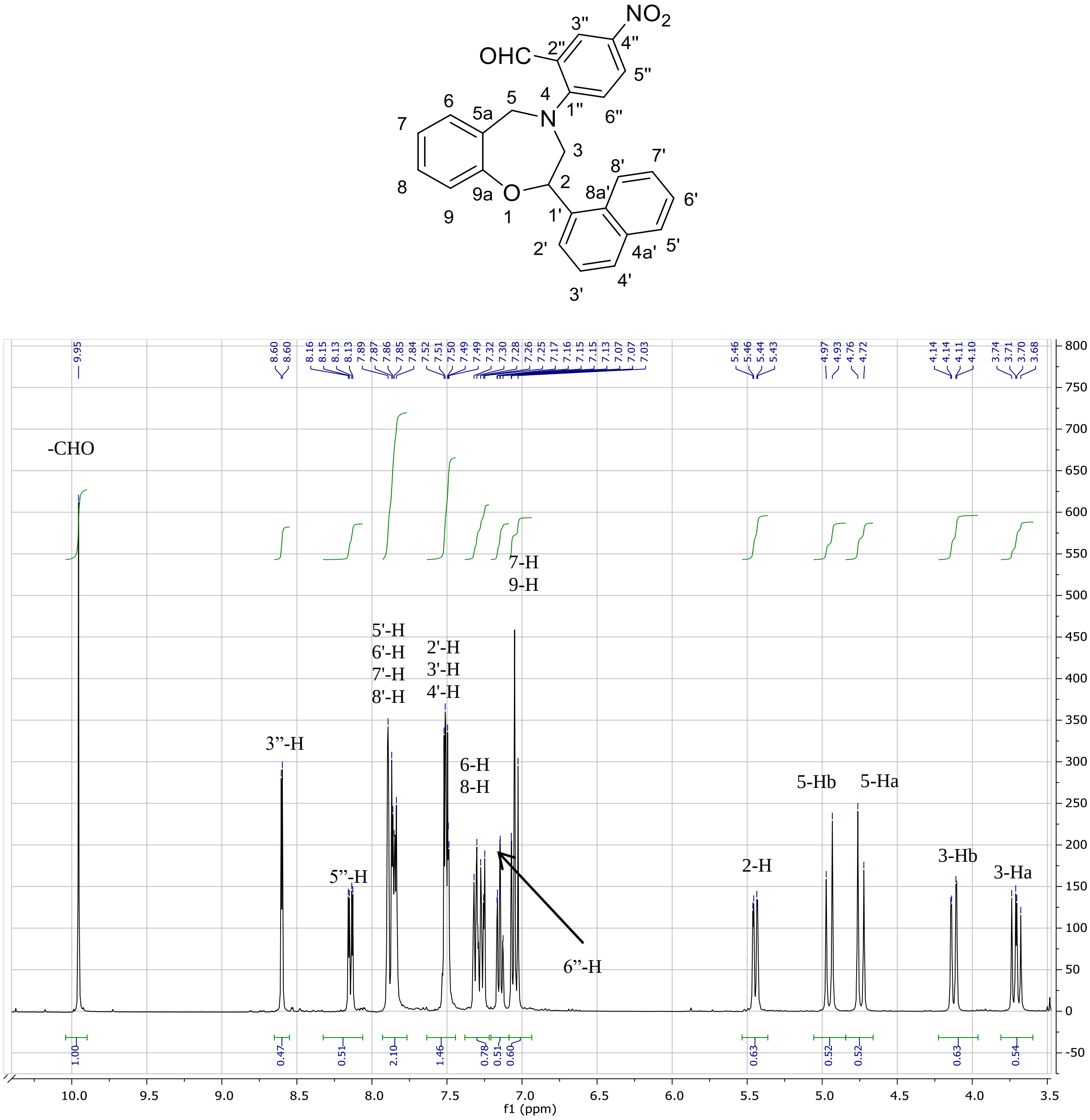

Figure S41. ^{1}H-NMR spectrum of *rac*-**1b** measured in $CDCl_3$ (400 MHz)

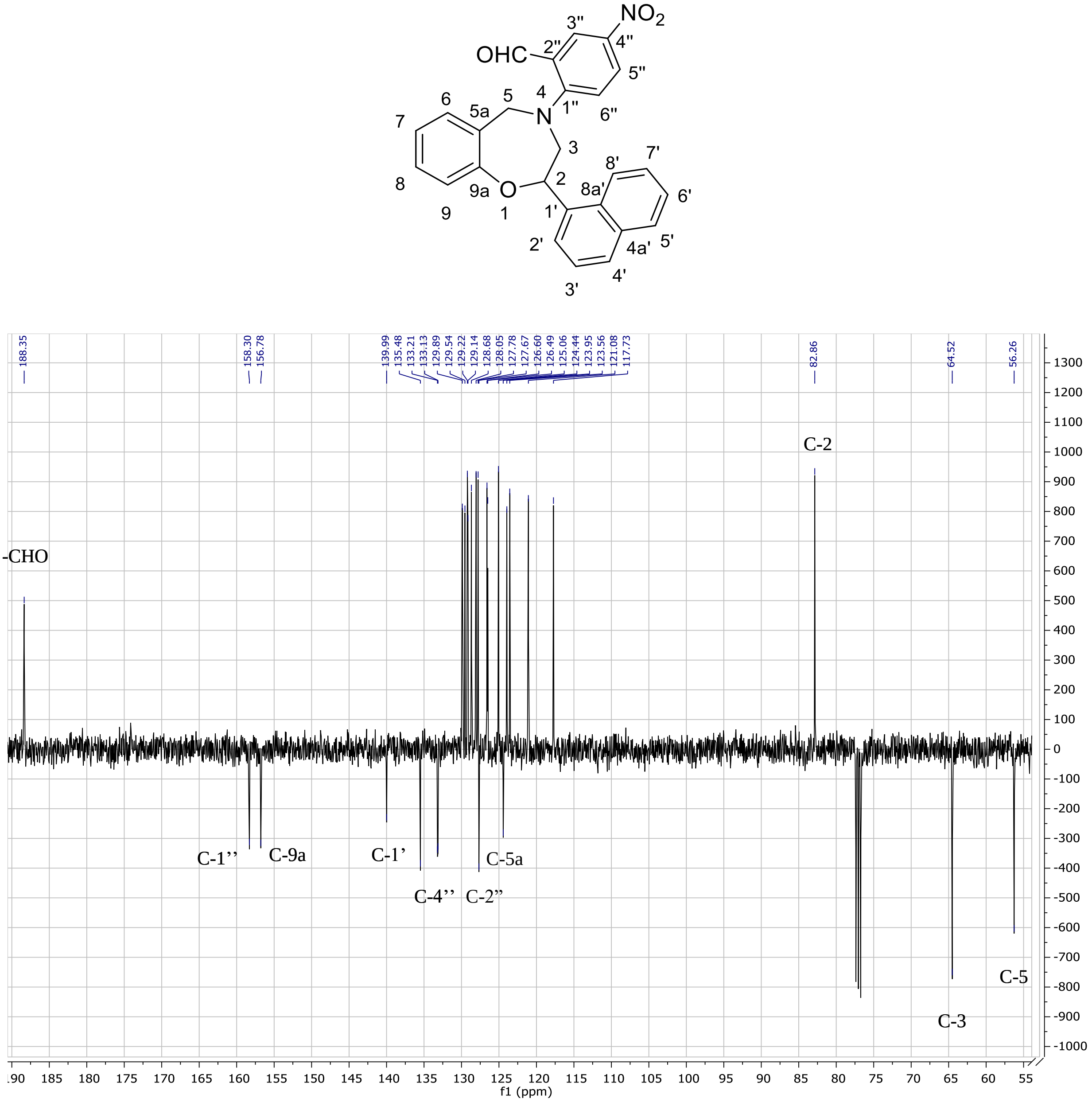

Figure S42. J-modulated ^{13}C-NMR spectrum of *rac*-**1b** measured in $CDCl_3$ (100 MHz)

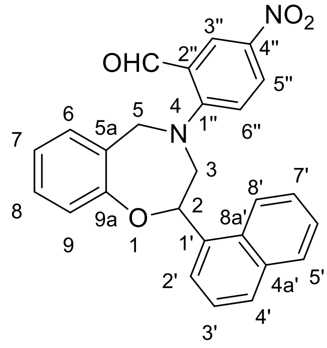

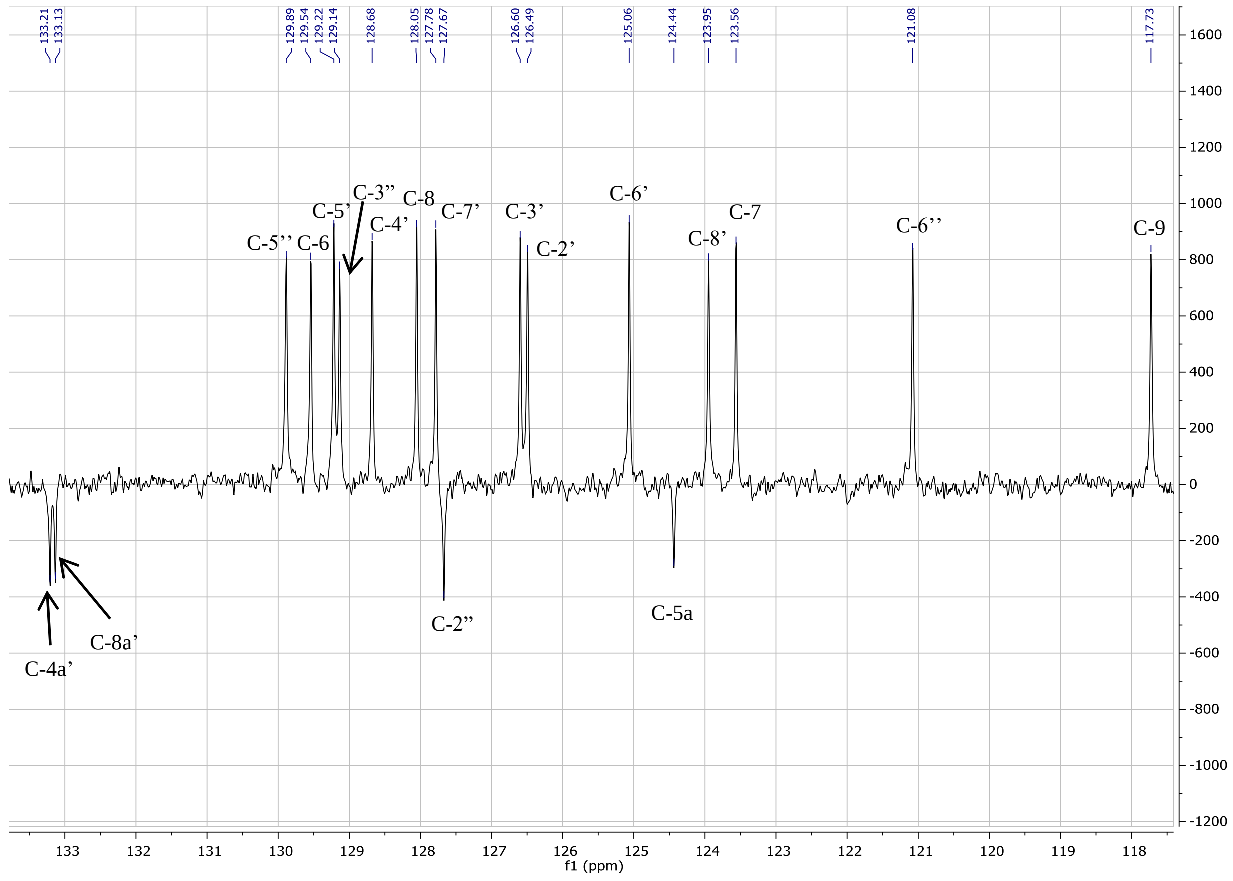

Figure S43. J-modulated ^{13}C-NMR spectrum of *rac*-**1b** measured in $CDCl_3$ (100 MHz)

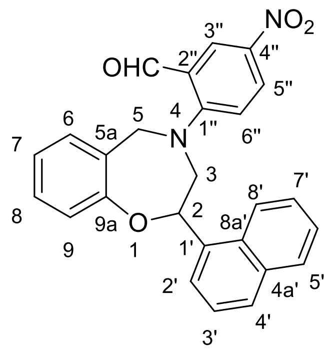

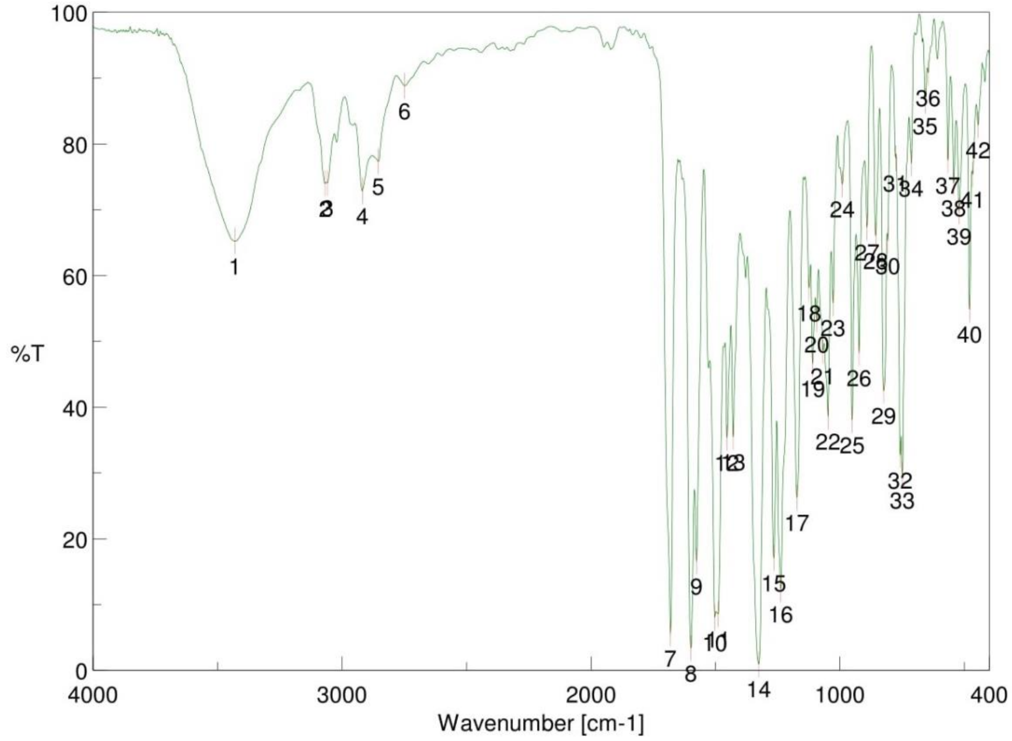

Figure S44. IR spectrum of *rac*-**1b** recorded as KBr disc

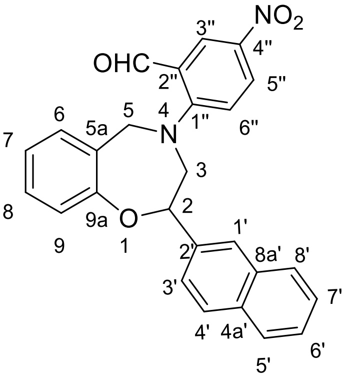

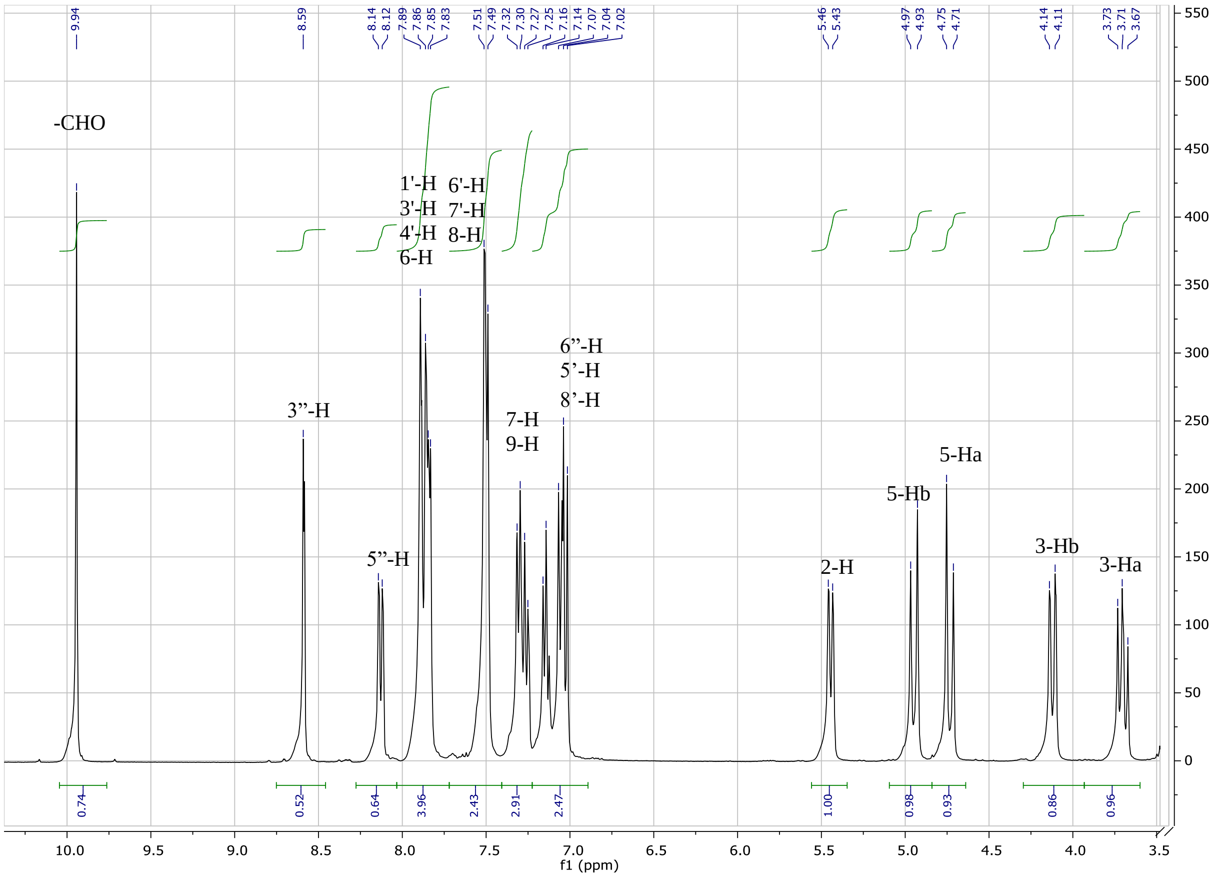

Figure S45. ^{1}H-NMR spectrum of *rac*-**1c** measured in $CDCl_3$ (400 MHz)

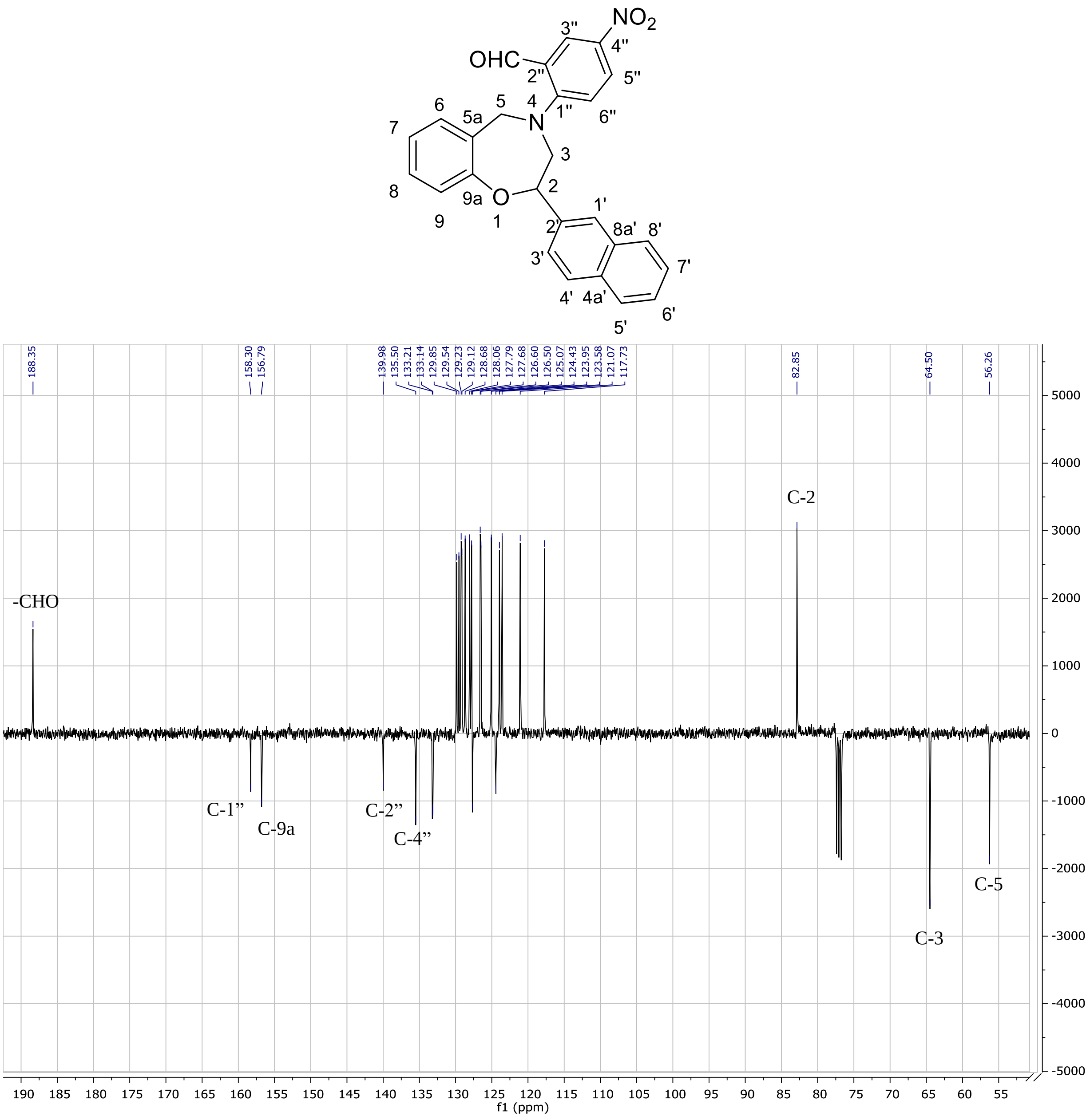

Figure S46. J-modulated ^{13}C-NMR spectrum of *rac*-**1c** measured in $CDCl_3$ (100 MHz)

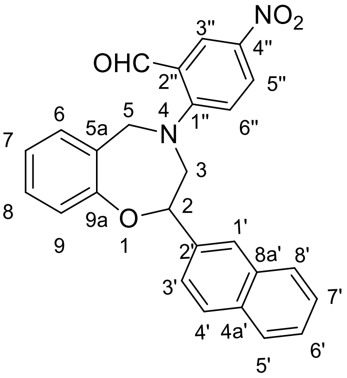

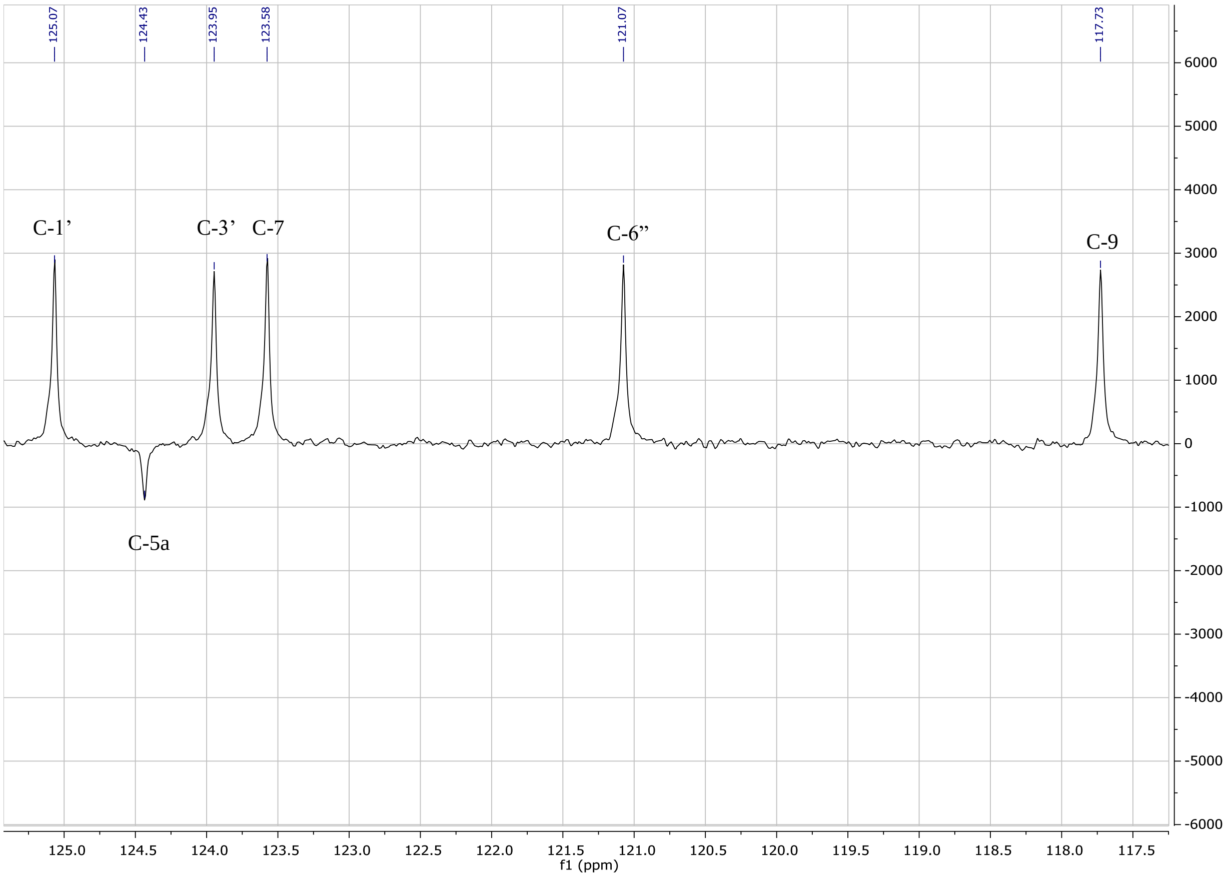

Figure S47. J-modulated ^{13}C-NMR spectrum of *rac*-**1c** measured in $CDCl_3$ (100 MHz)

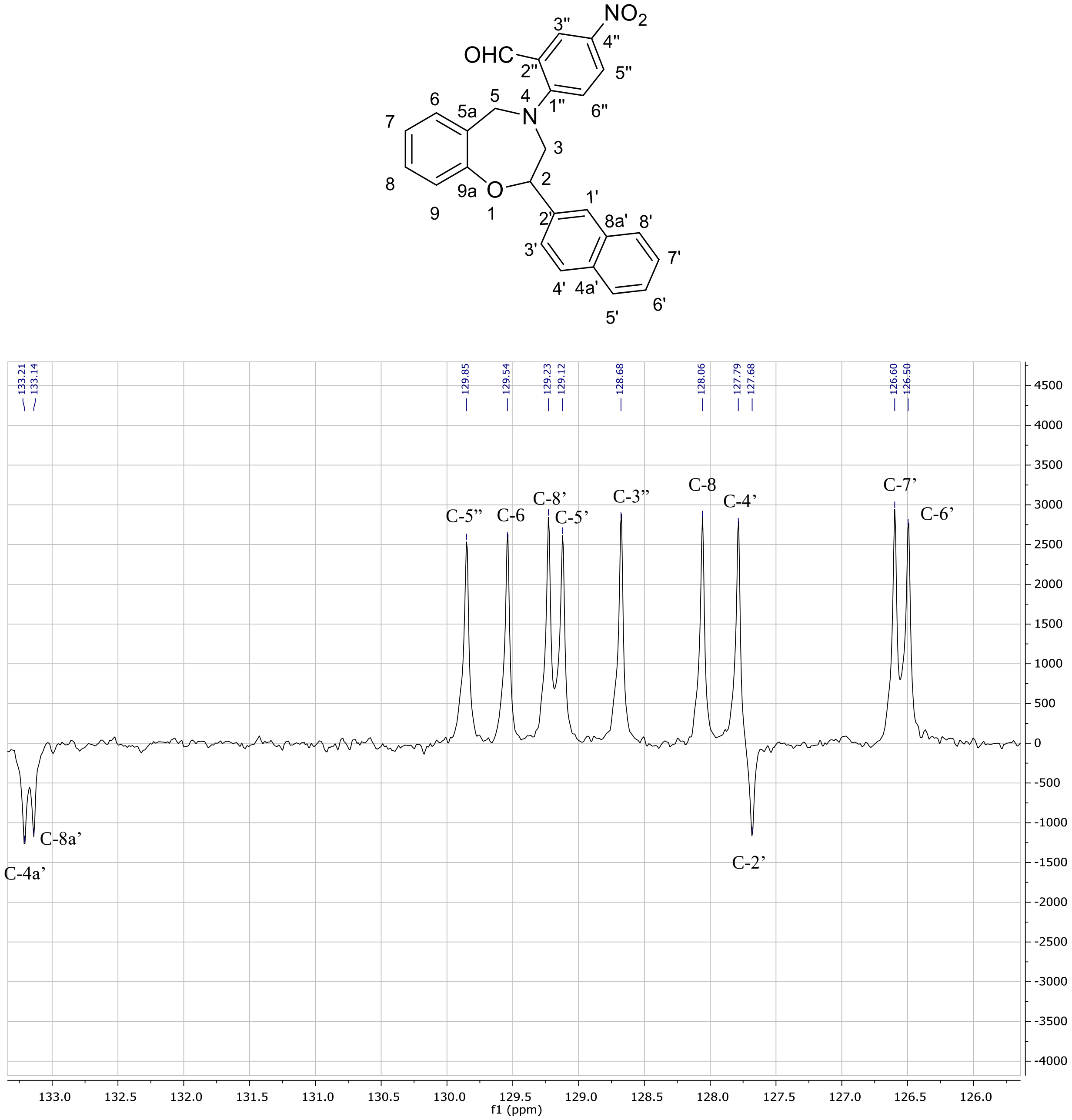

Figure S48. J-modulated ^{13}C-NMR spectrum of *rac*-**1c** measured in $CDCl_3$ (100 MHz)

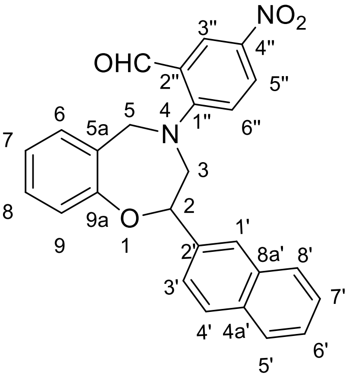

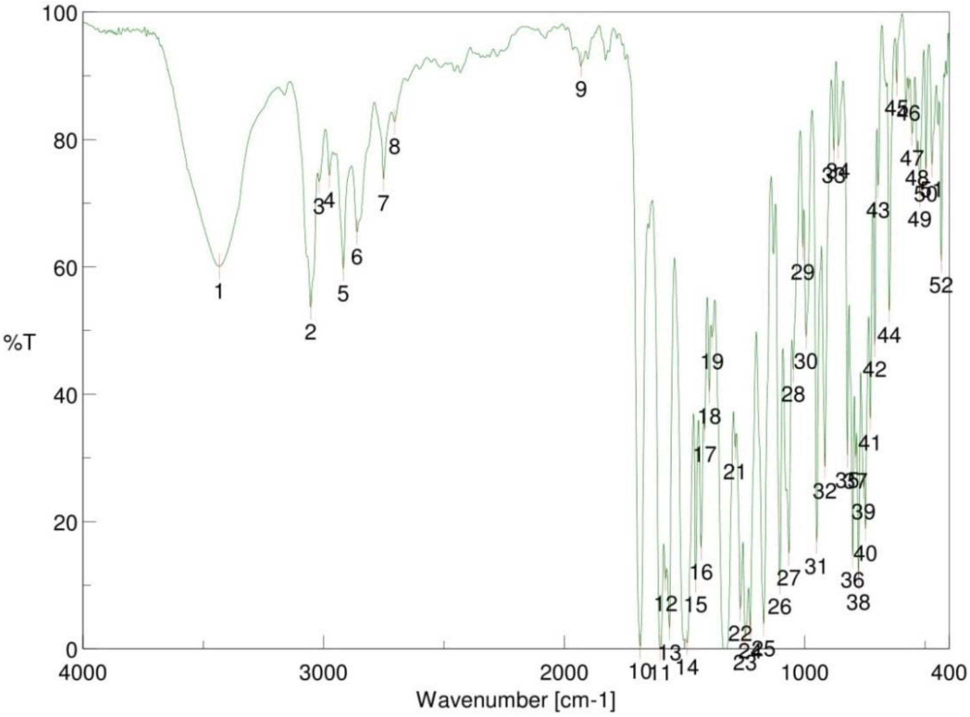

Figure S49. IR spectrum of *rac*-**1c** recorded as KBr disc

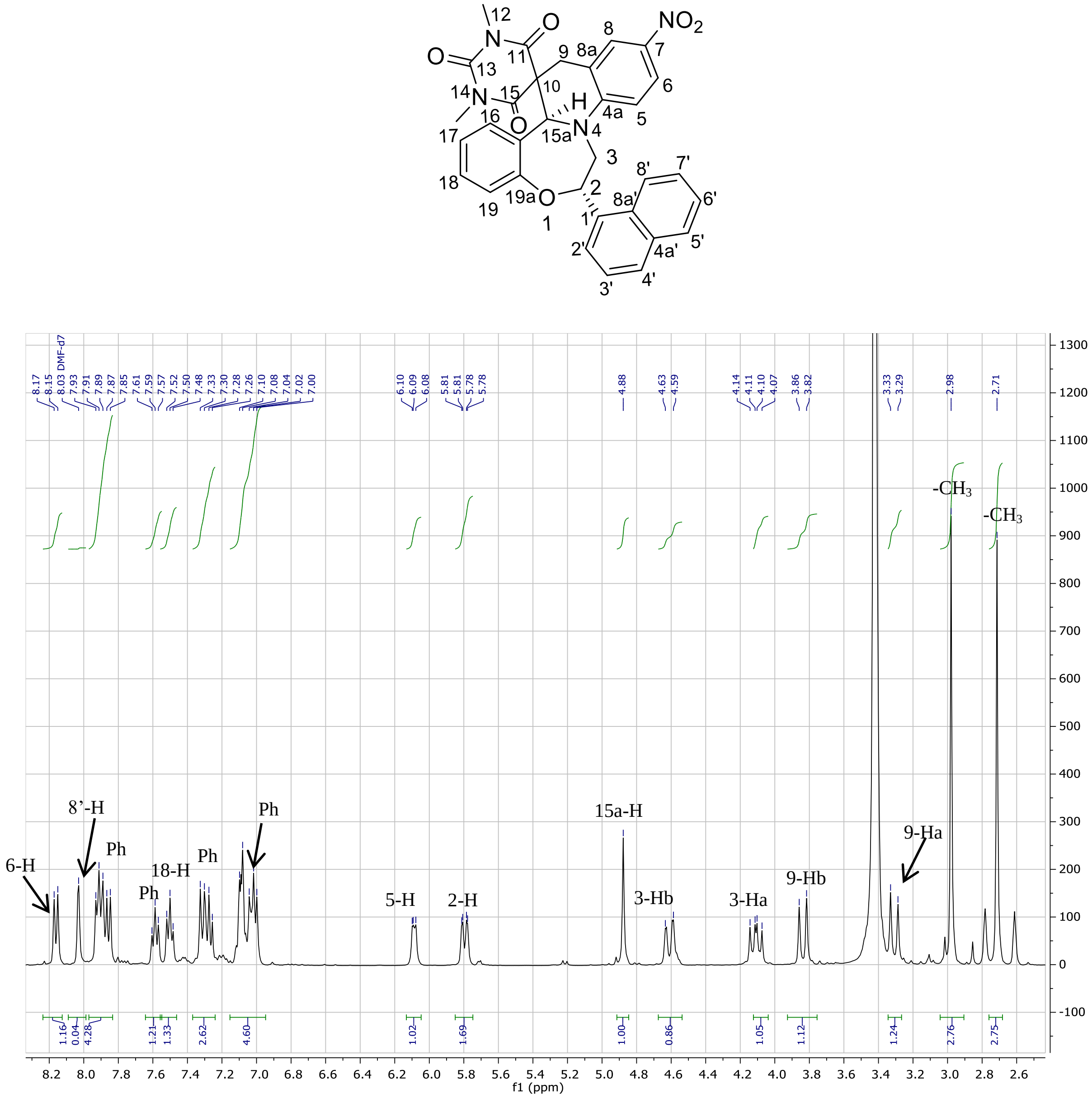

Figure S50. ^{1}H-NMR spectrum of *rac*-**10b** measured in DMF-d$_7$ (400 MHz)

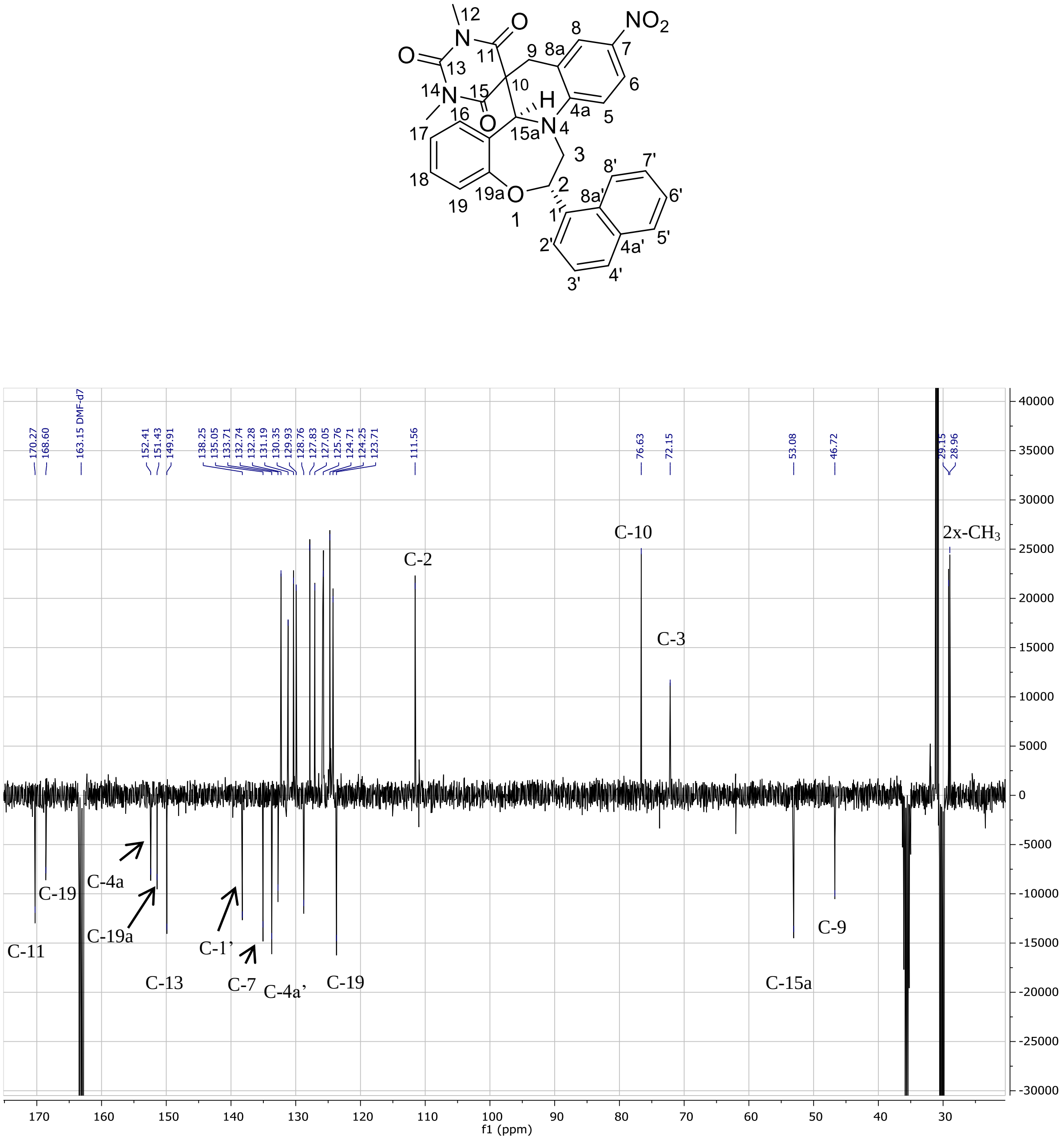

Figure S51. J-modulated ^{13}C-NMR spectrum of *rac*-**10b** measured in DMF-d_7 (100 MHz)

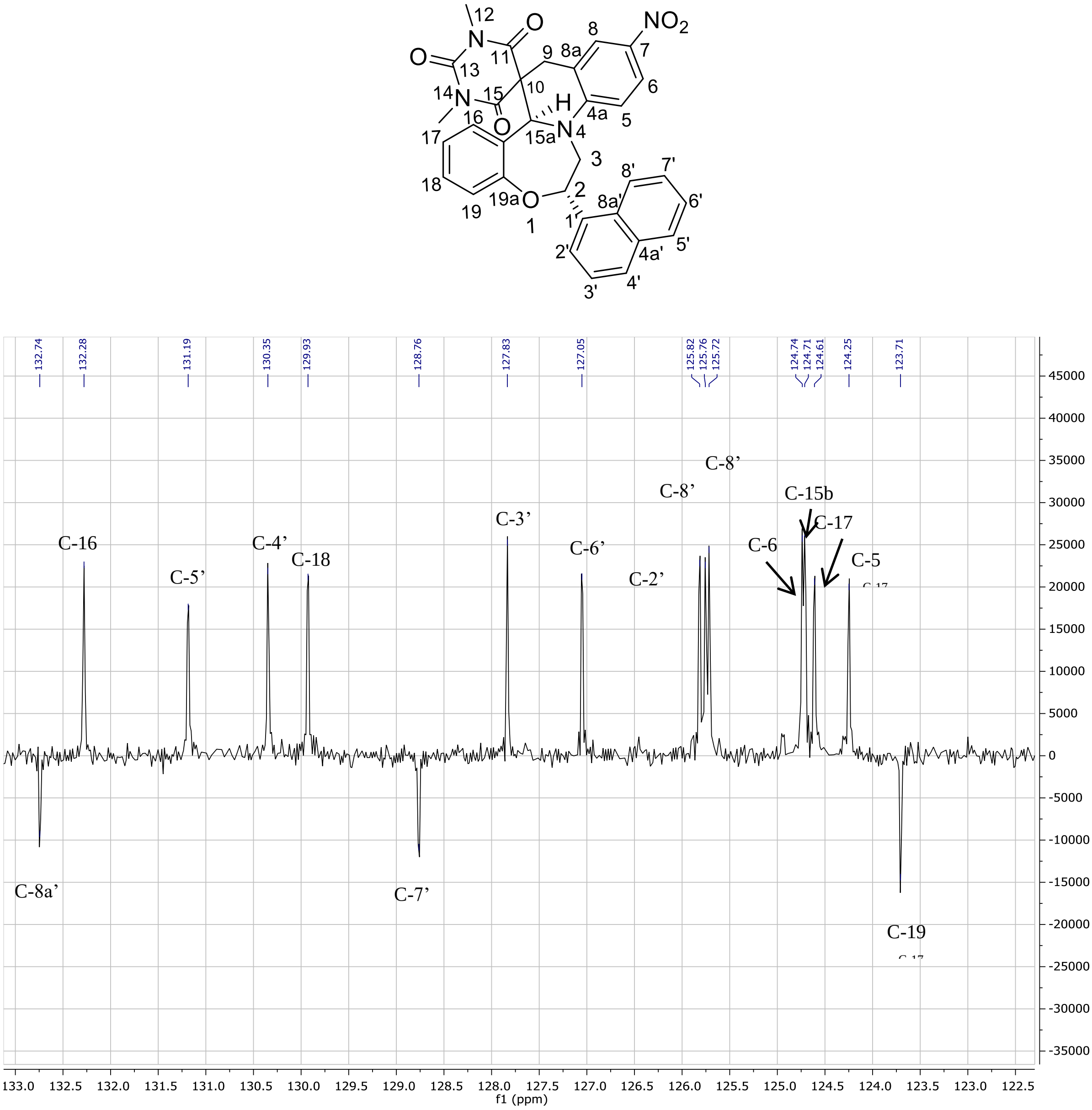

Figure S52. J-modulated ^{13}C-NMR spectrum of *rac*-**10b** measured in DMF-d$_7$ (100 MHz)

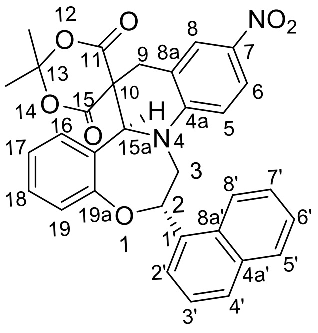

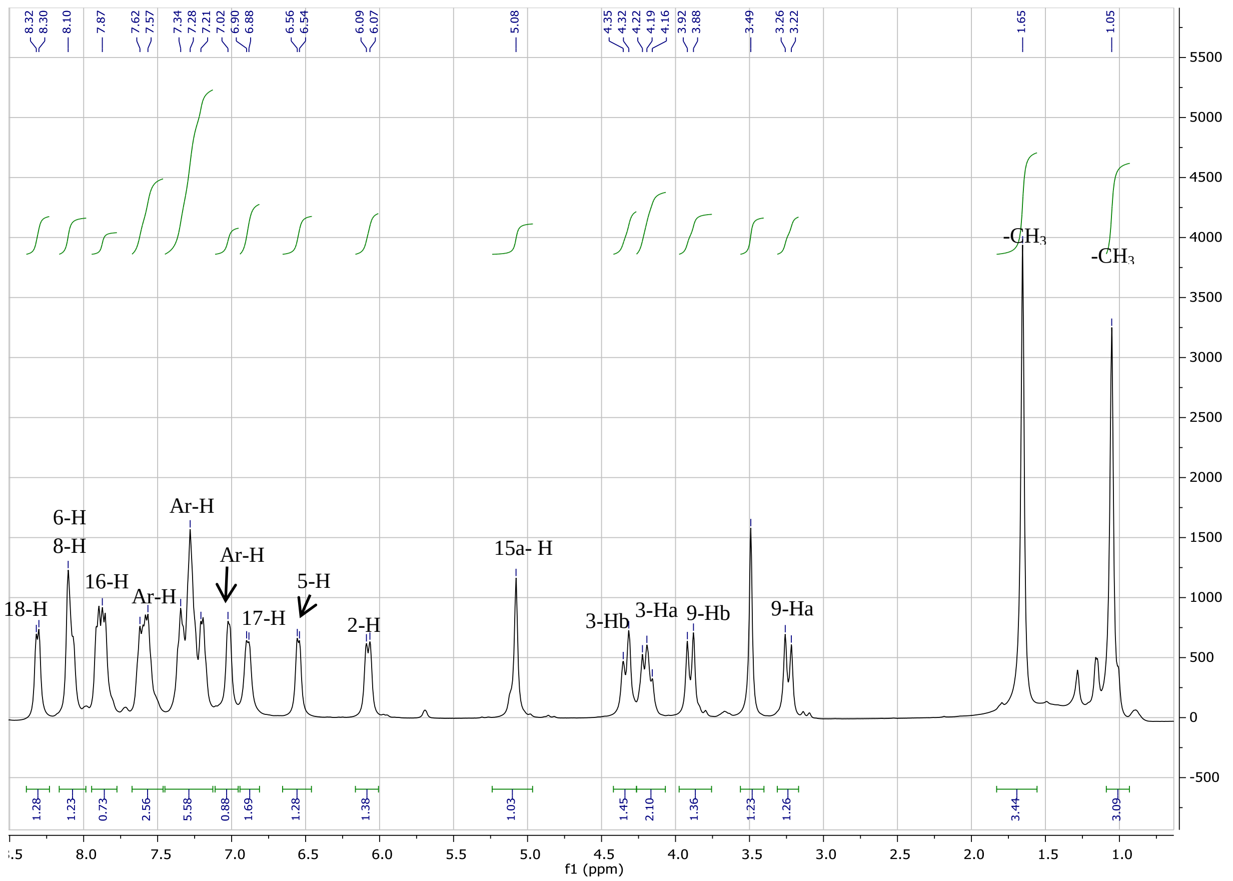

Figure S53. ^{1}H-NMR spectrum of *rac*-**11b** measured in $CDCl_3$ (400 MHz)

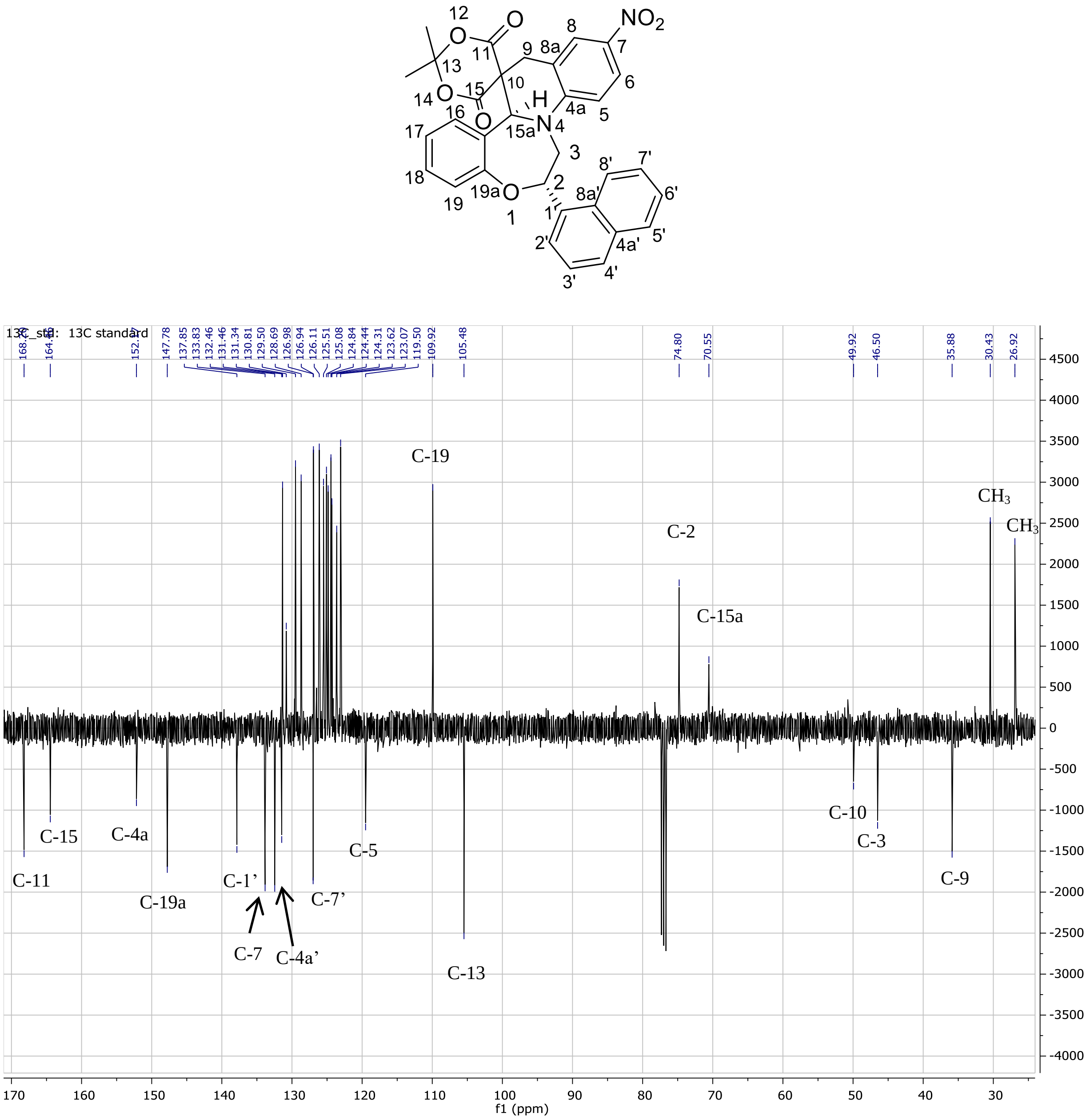

Figure S54. J-modulated ^{13}C-NMR spectrum of *rac*-**11b** measured in $CDCl_3$ (100 MHz)

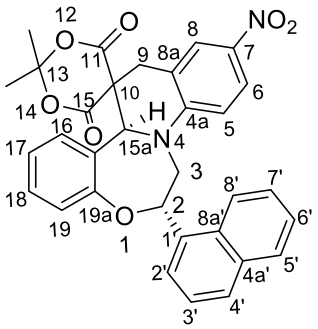

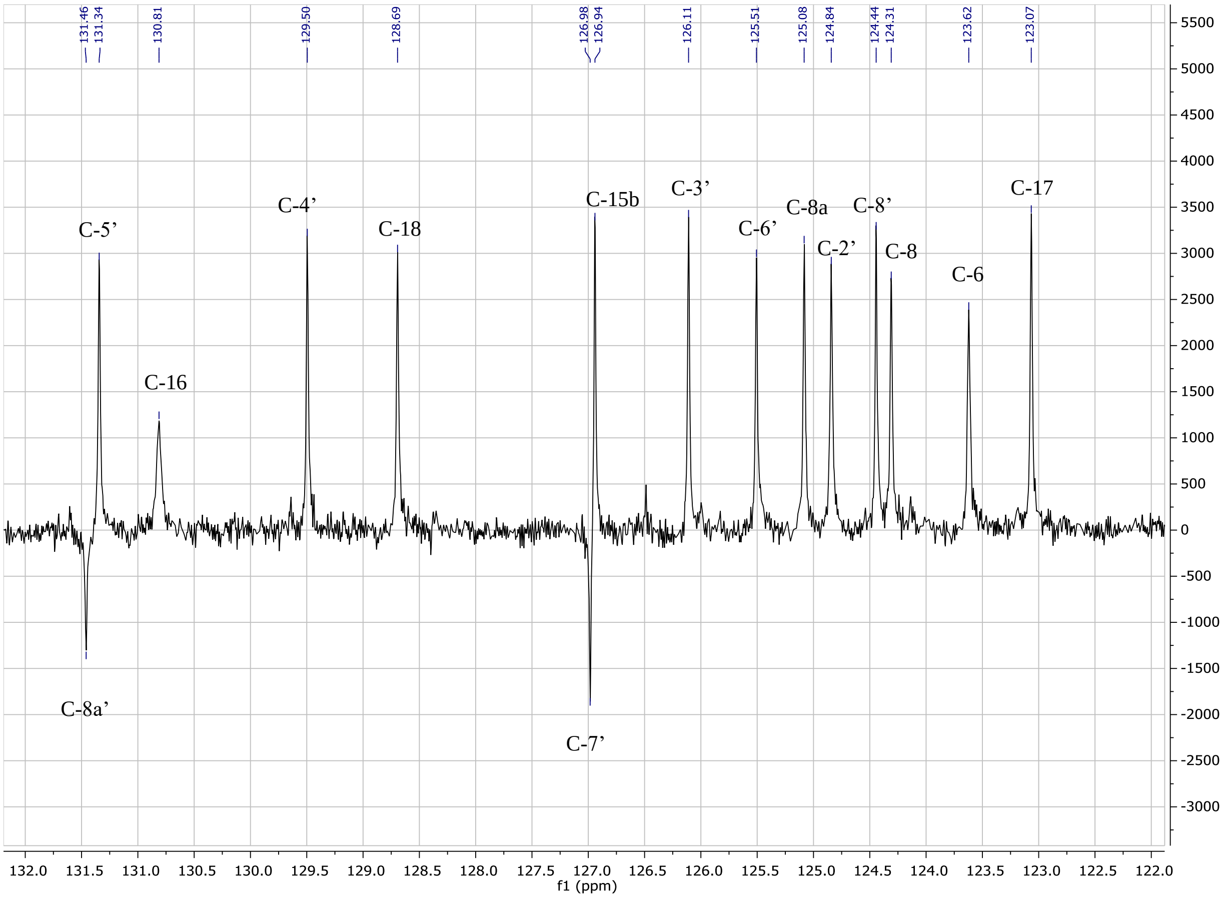

Figure S55. J-modulated ^{13}C-NMR spectrum of *rac*-**11b** measured in $CDCl_3$ (100 MHz)

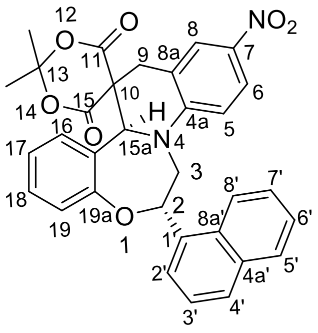

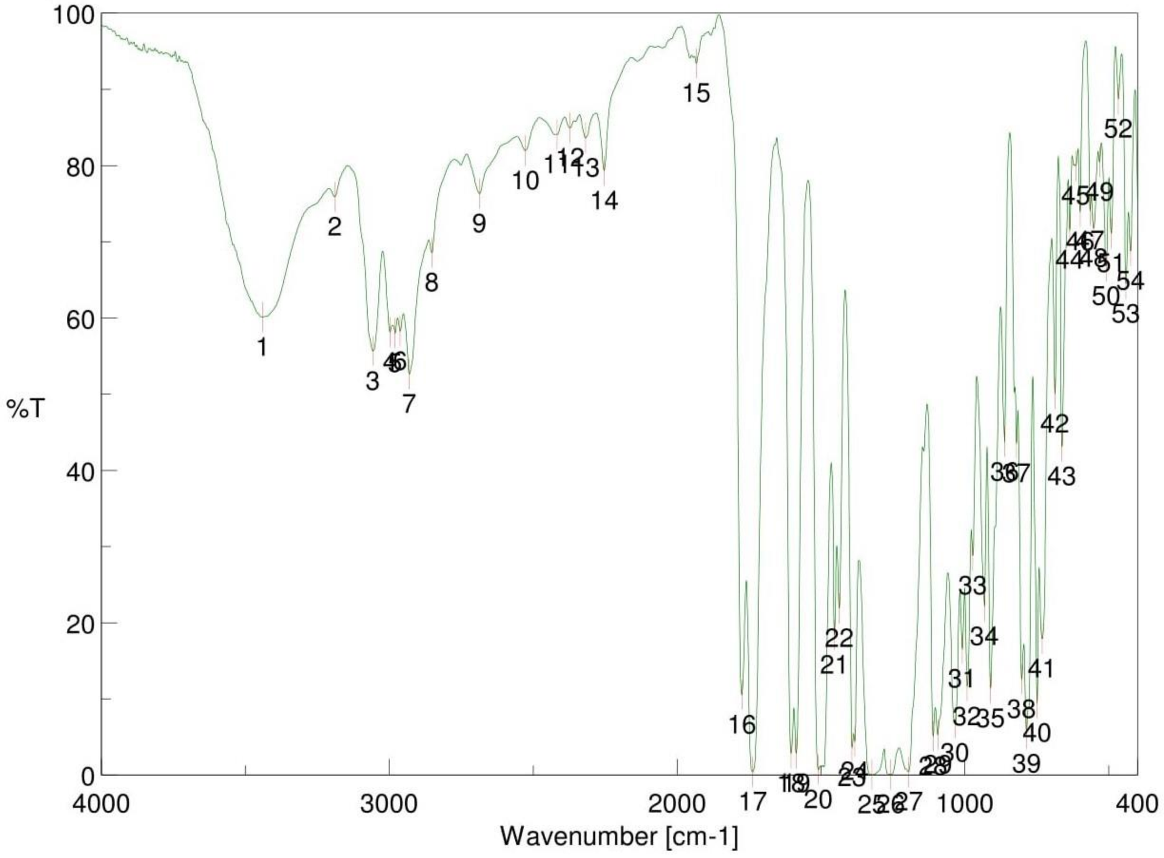

Figure S56. IR spectrum of *rac*-**11b** recorded as KBr disc

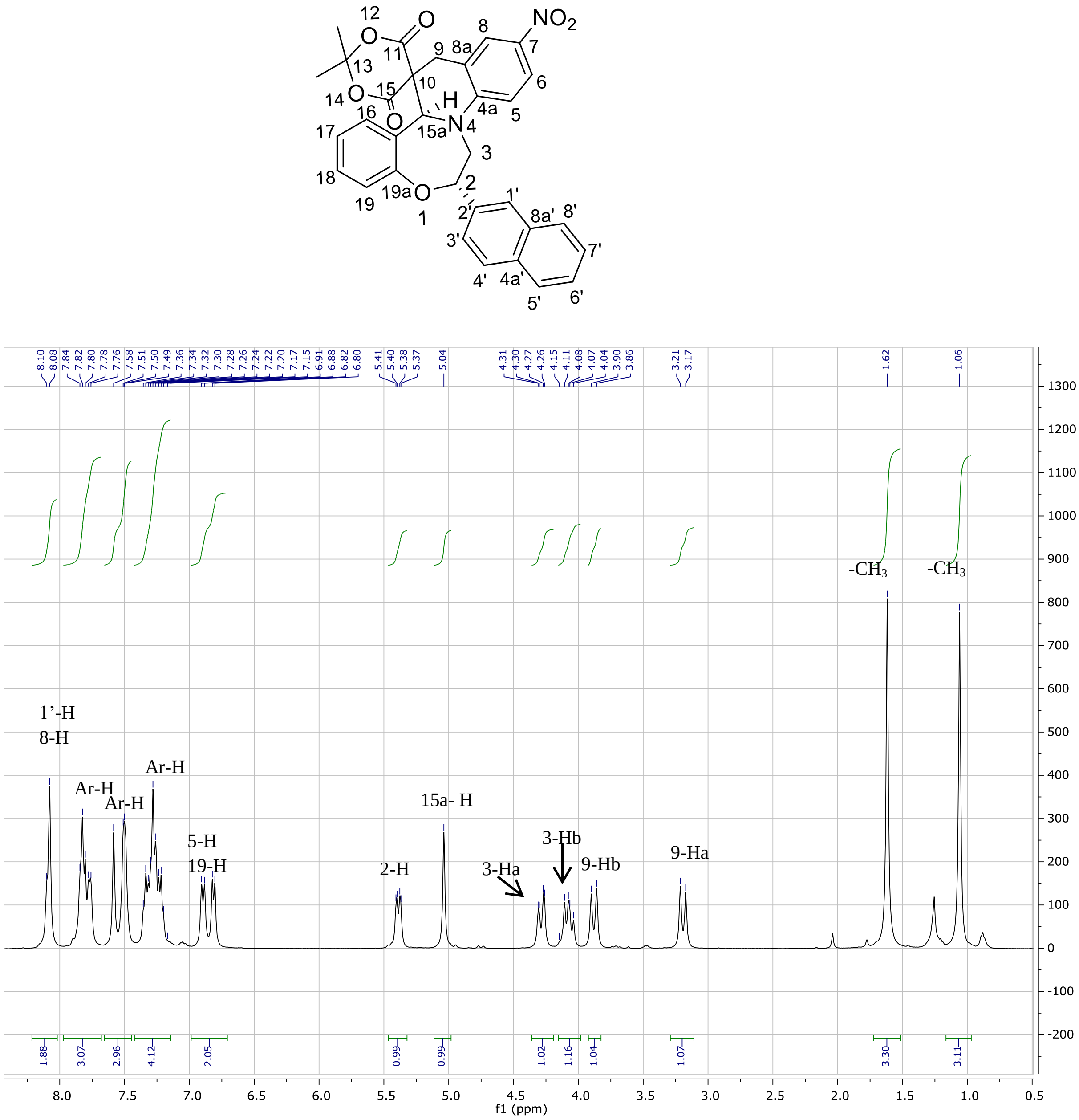

Figure S57. ^{1}H-NMR spectrum of *rac*-**11c** measured in $CDCl_3$ (400 MHz)

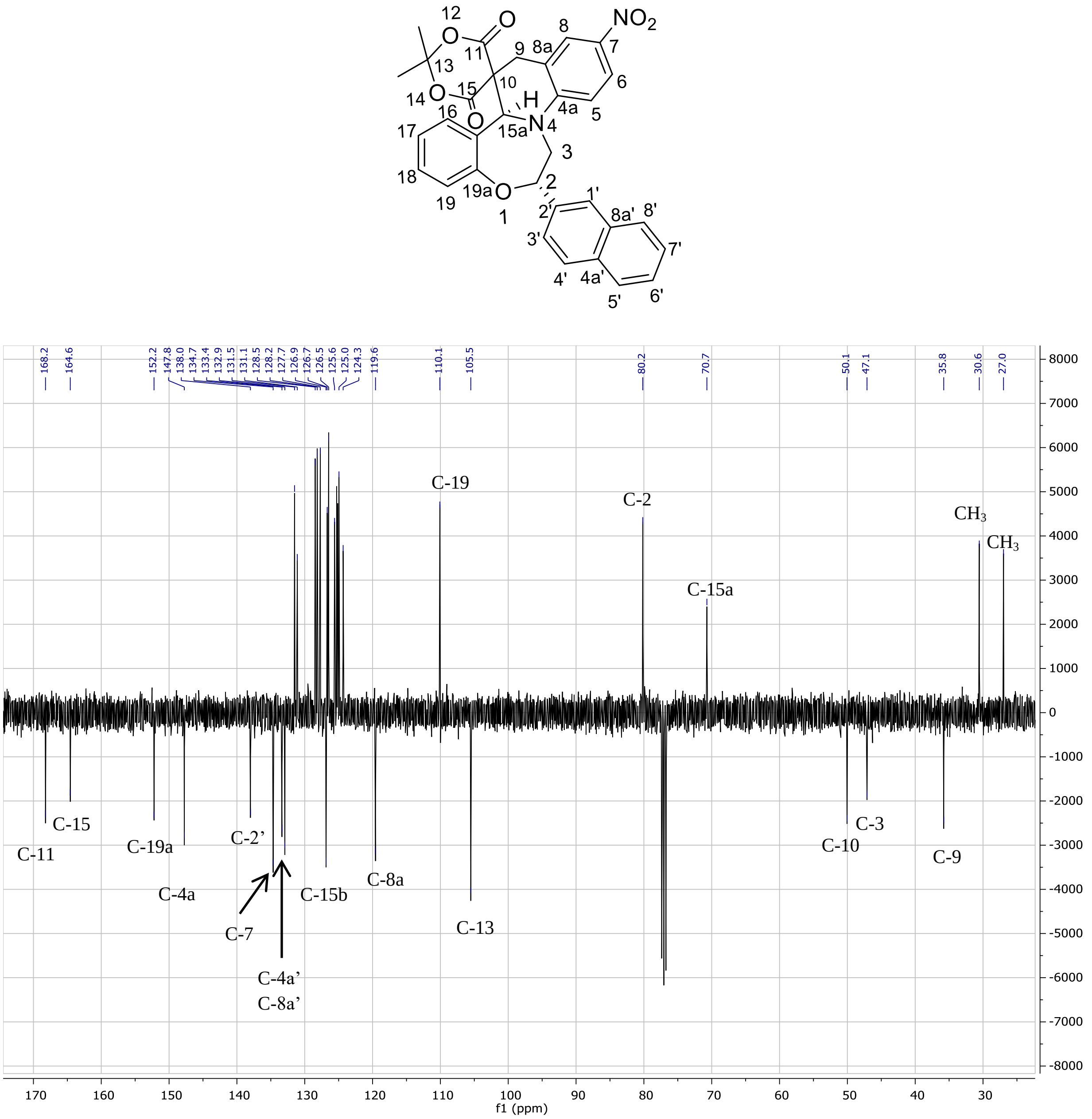

Figure S58. J-modulated ^{13}C-NMR spectrum of *rac*-**11c** measured in $CDCl_3$ (100 MHz)

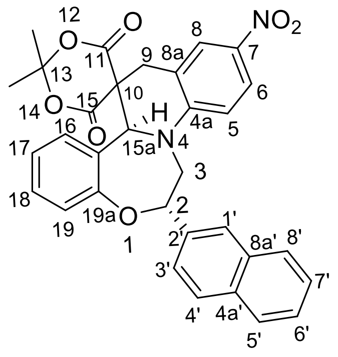

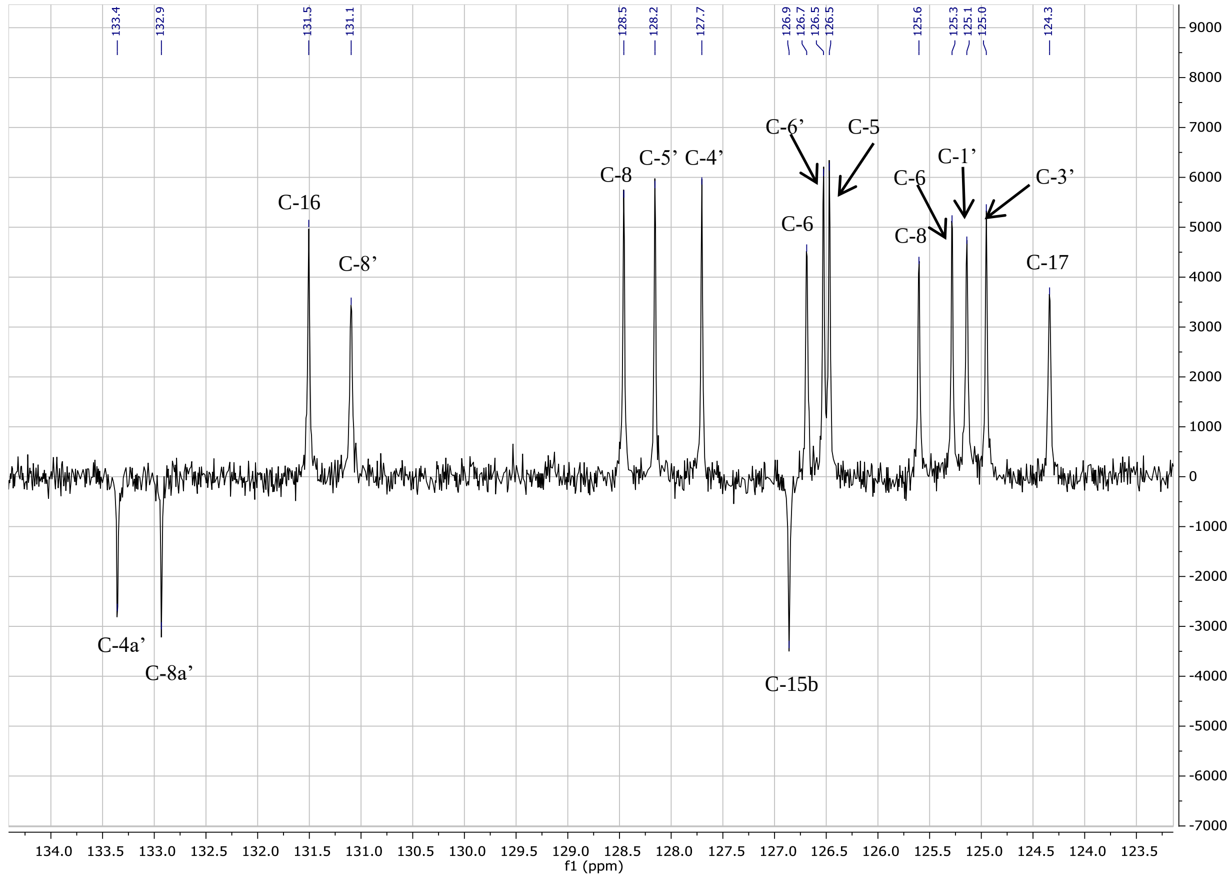

Figure S59. J-modulated ^{13}C-NMR spectrum of *rac*-**11c** measured in $CDCl_3$ (100 MHz)

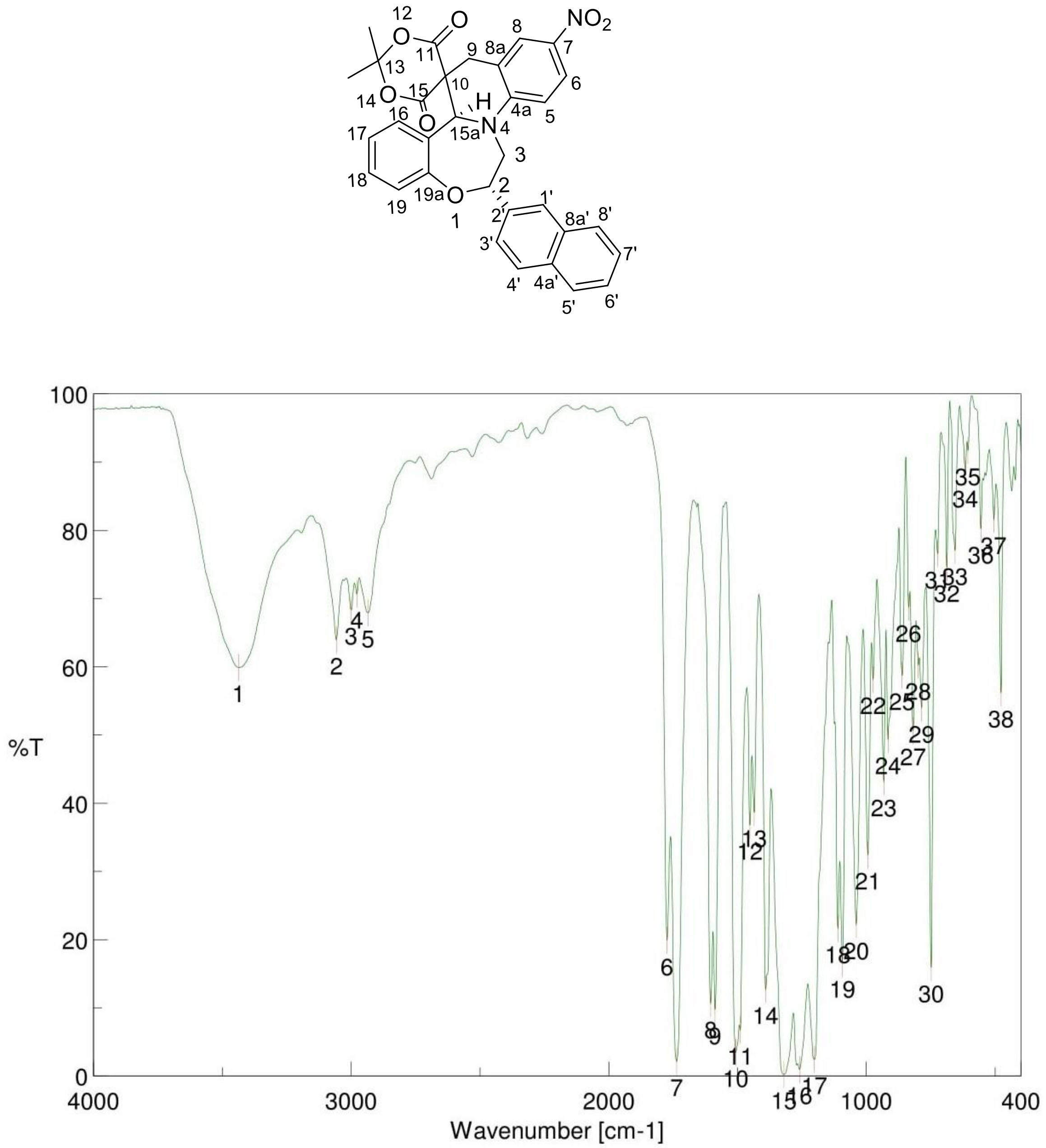

Figure S60. IR spectrum of *rac*-**11c** recorded as KBr disc

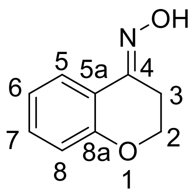

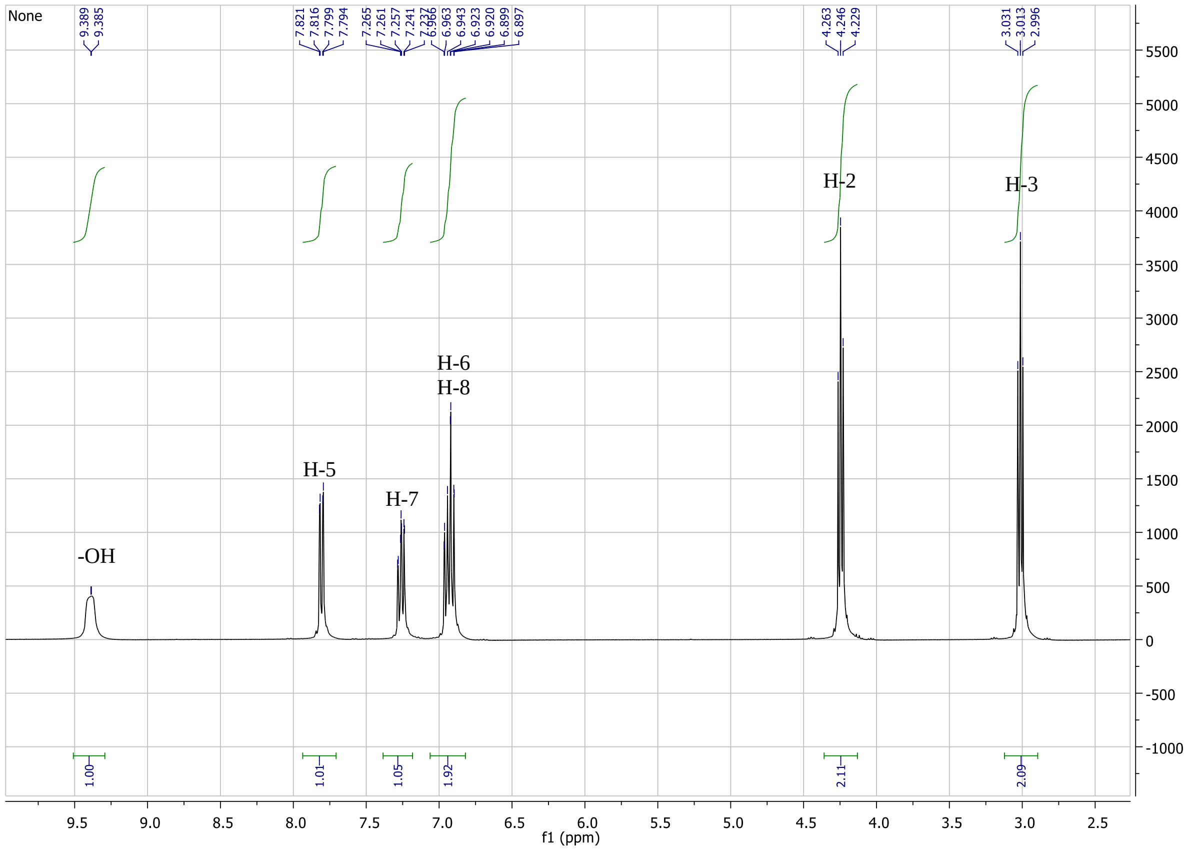

Figure S61. ^{1}H-NMR spectrum of **13** measured in $CDCl_3$ (360 MHz)

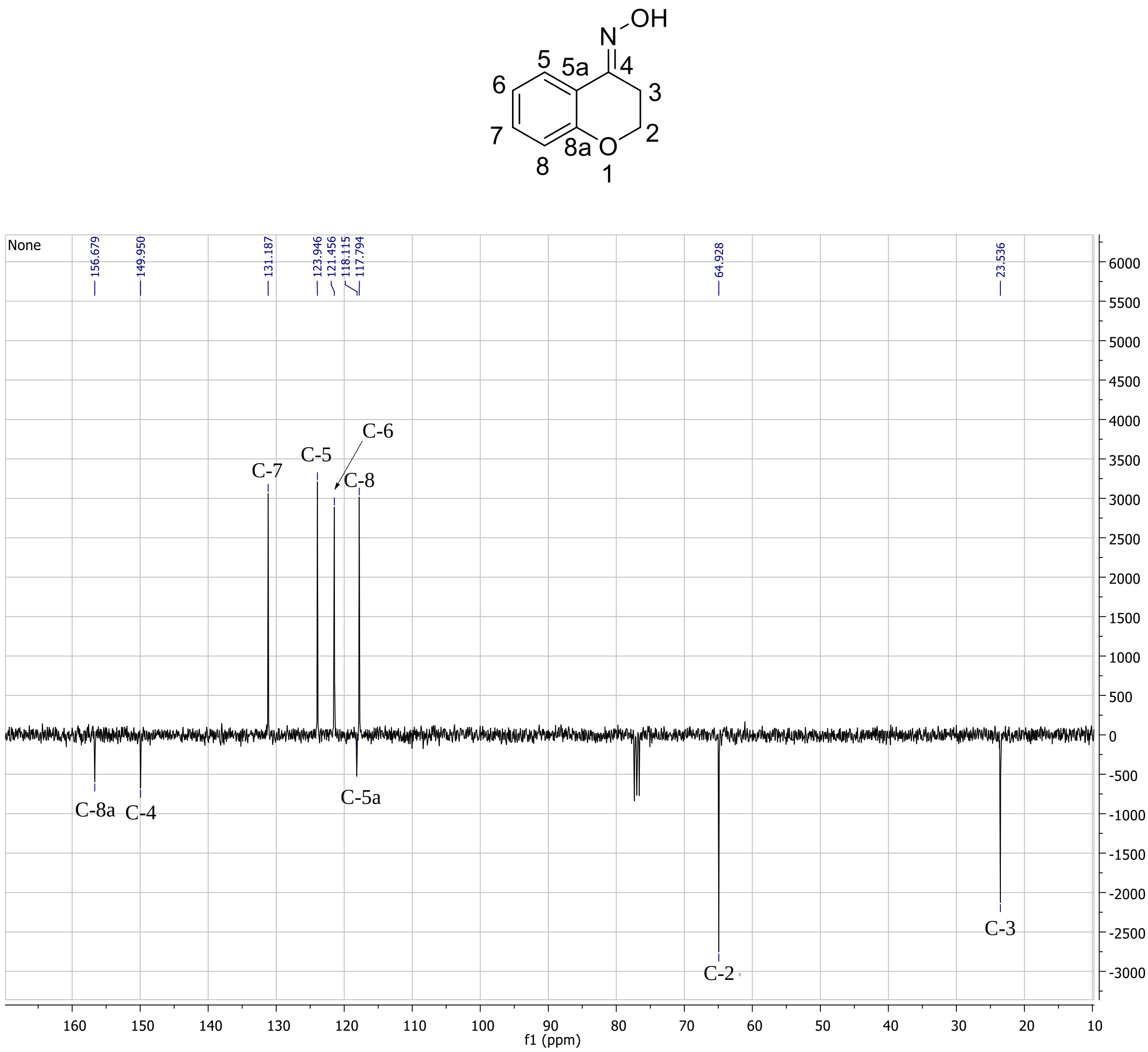

Figure S65. J-modulated ^{13}C-NMR spectrum of **13** measured in $CDCl_3$ (90 MHz)

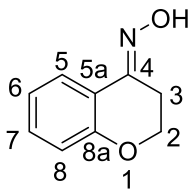

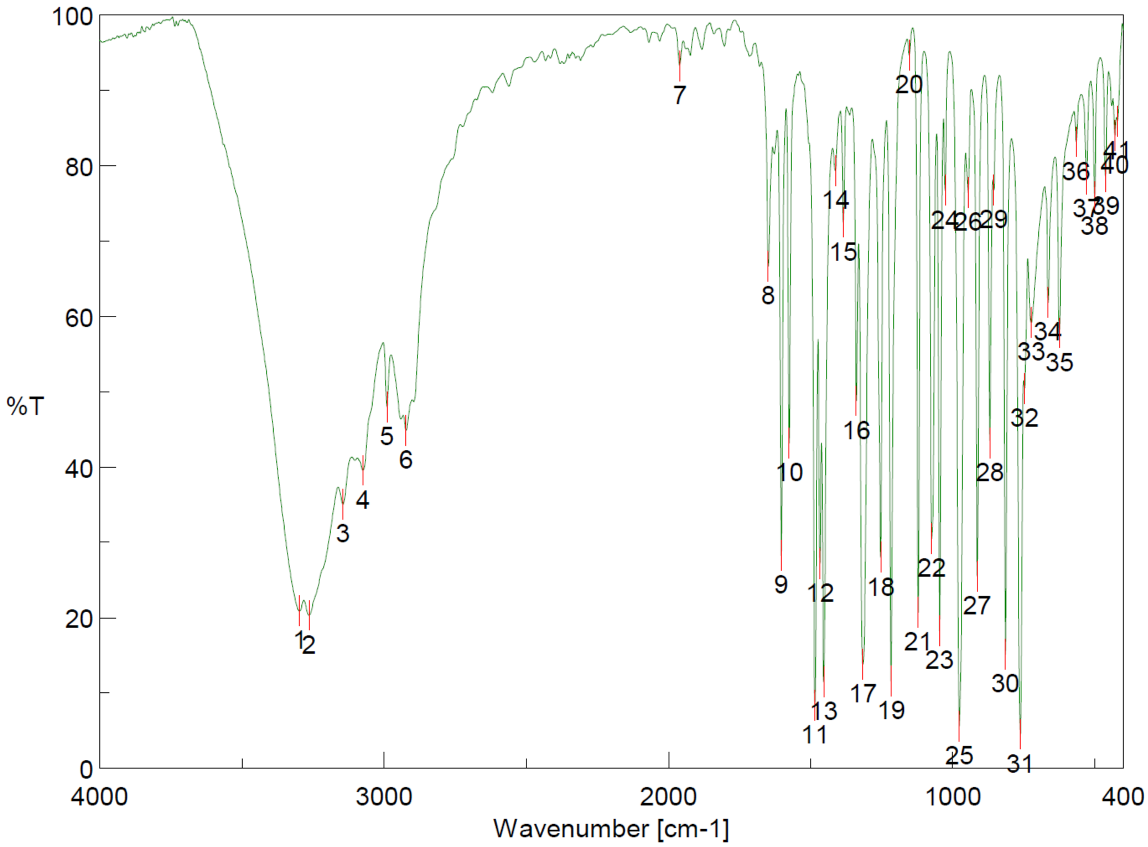

Figure S66. IR spectrum of **13** recorded as KBr disc

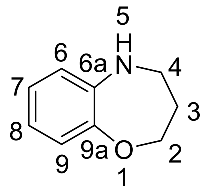

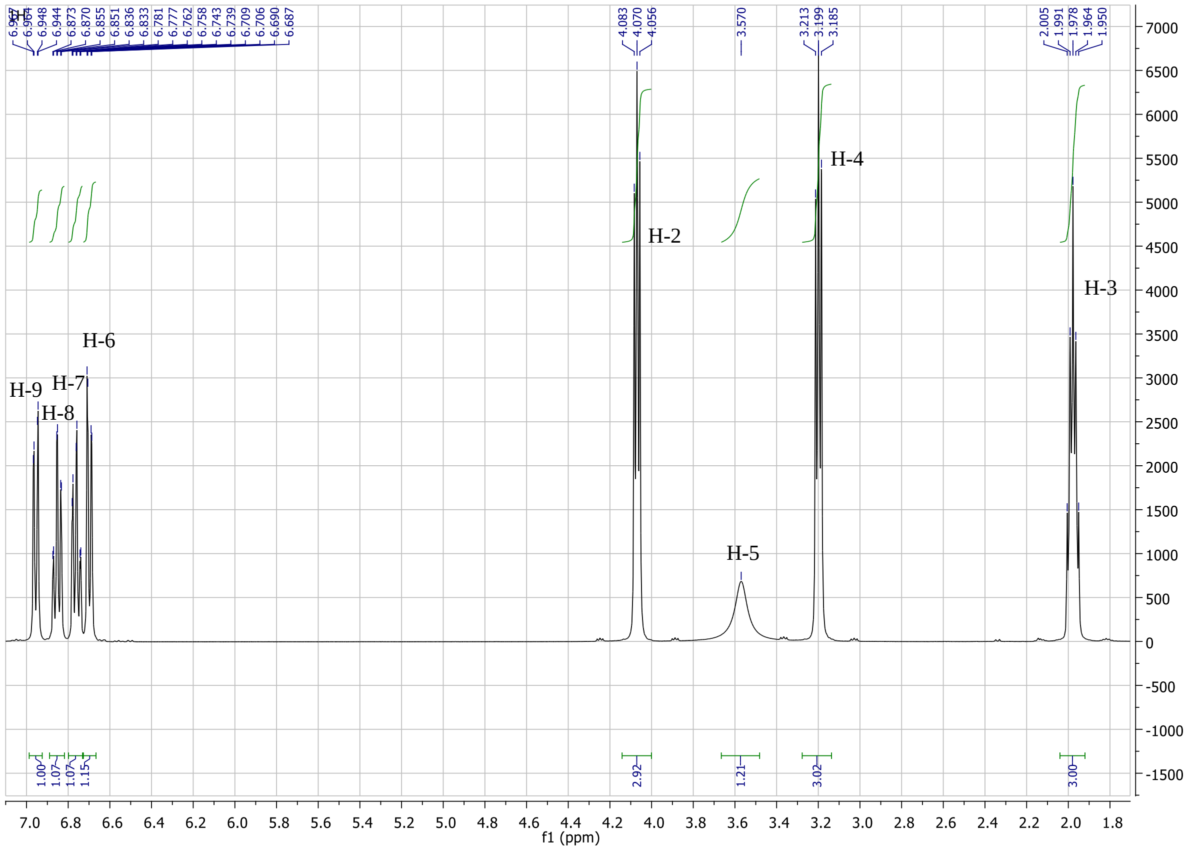

Figure S67. ^{1}H-NMR spectrum of **15** measured in $CDCl_3$ (400 MHz)

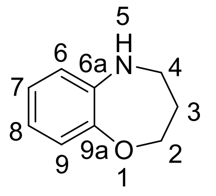

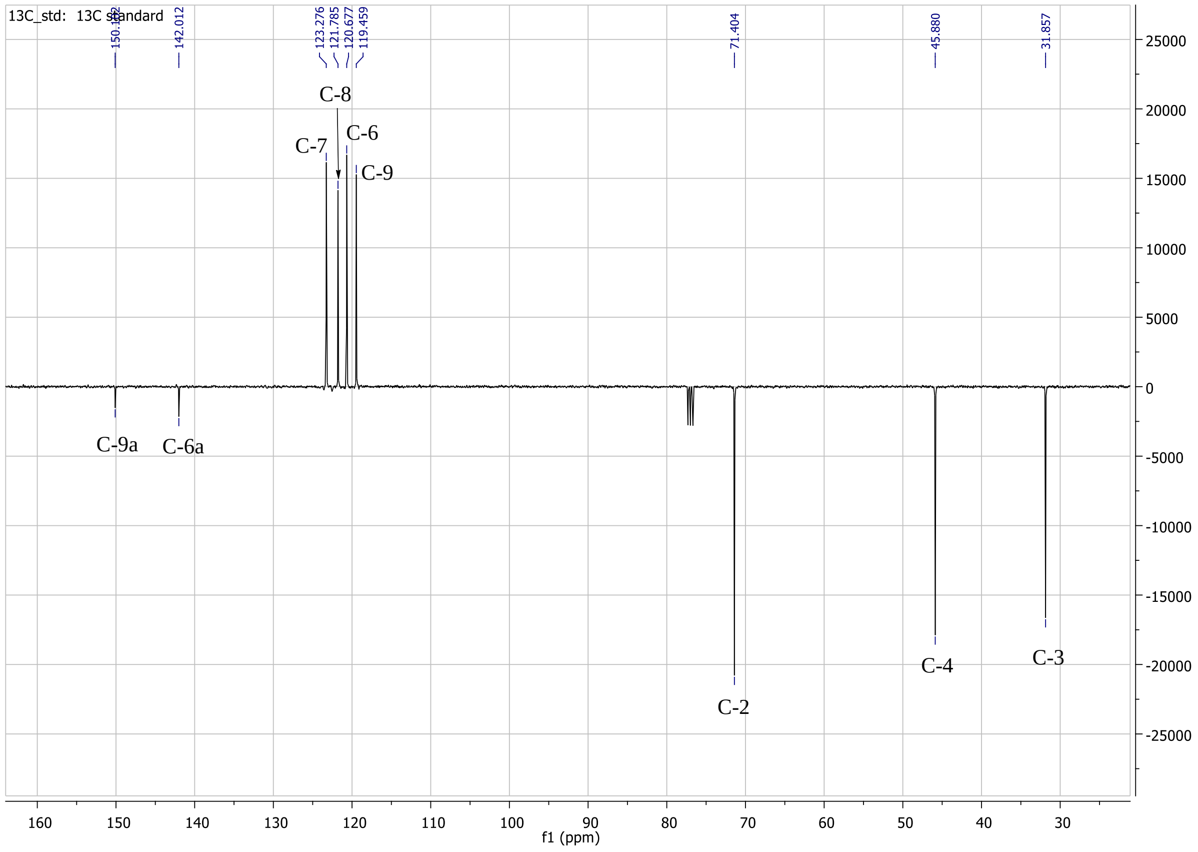

Figure S68. J-modulated ^{13}C-NMR spectrum of **15** measured in $CDCl_3$ (100 MHz)

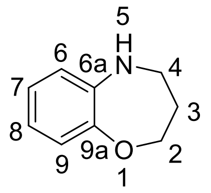

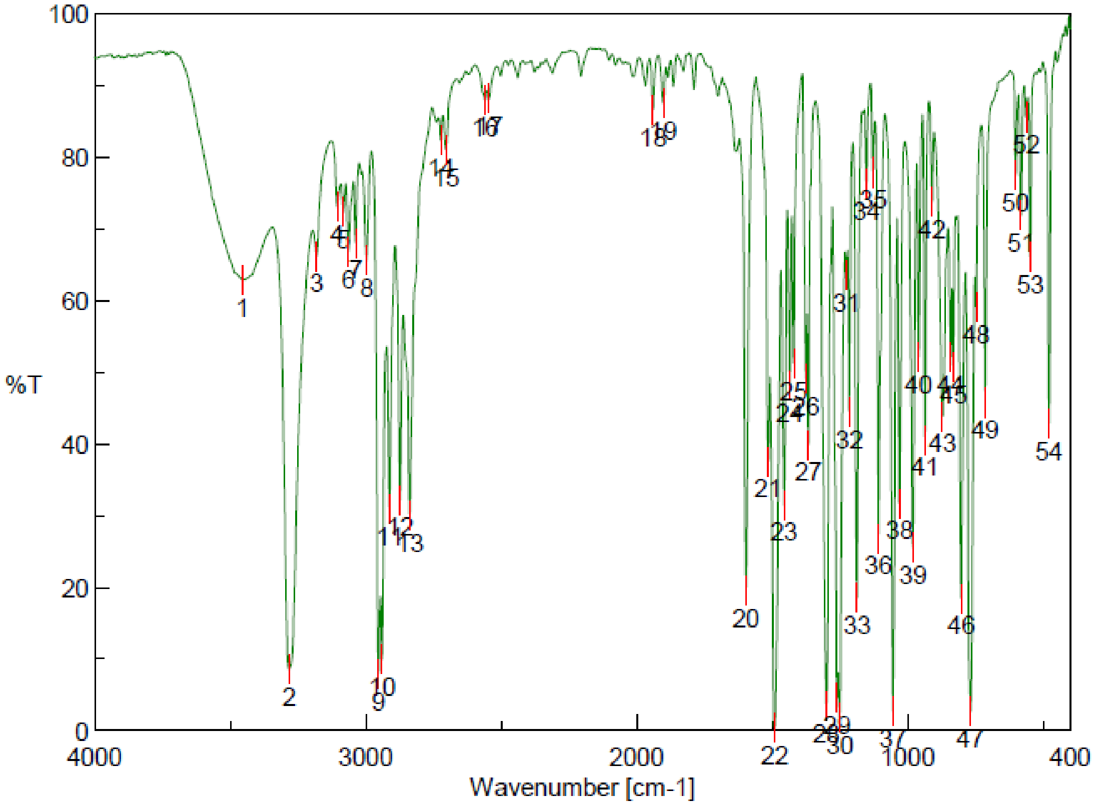

Figure S69. IR spectrum of **15** recorded as KBr disc

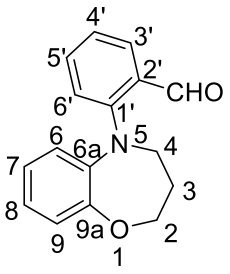

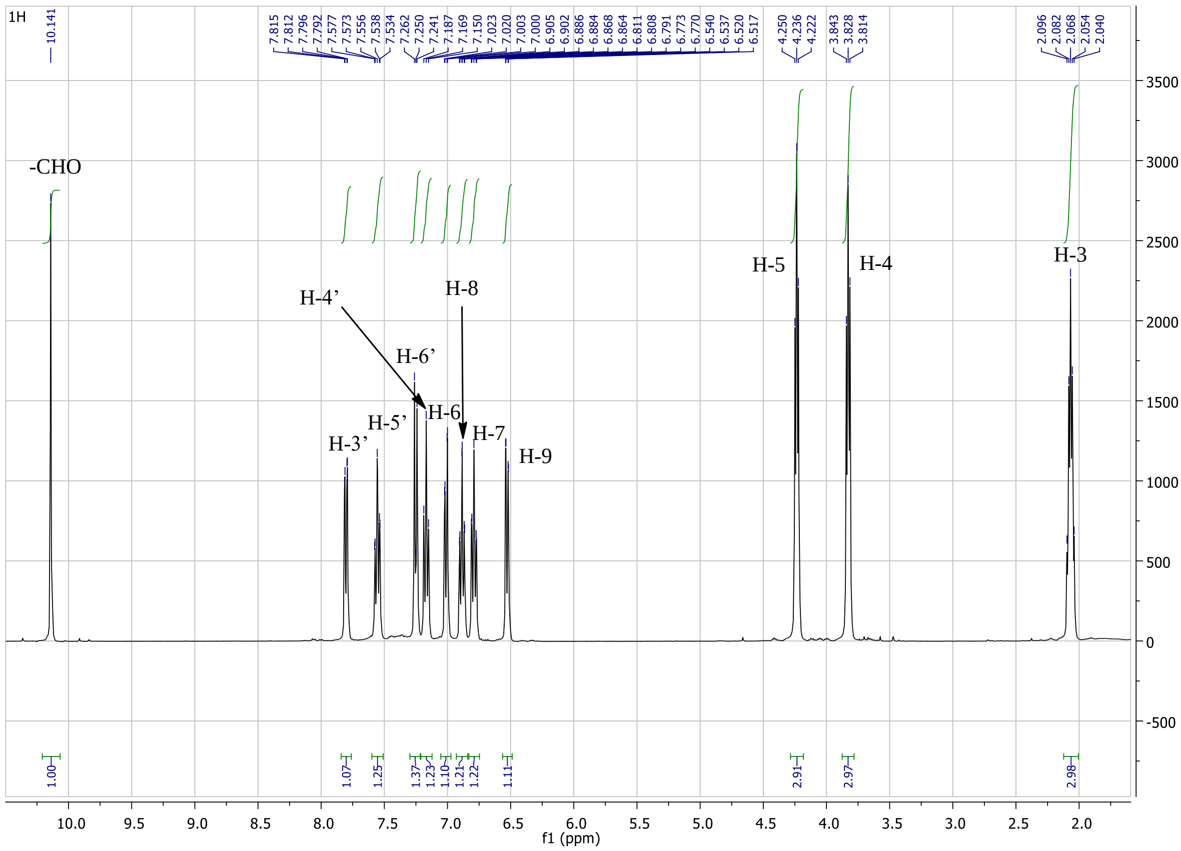

Figure S70. ^{1}H-NMR spectrum of **4** measured in $CDCl_3$ (400 MHz)

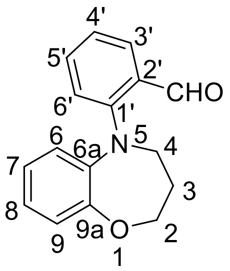

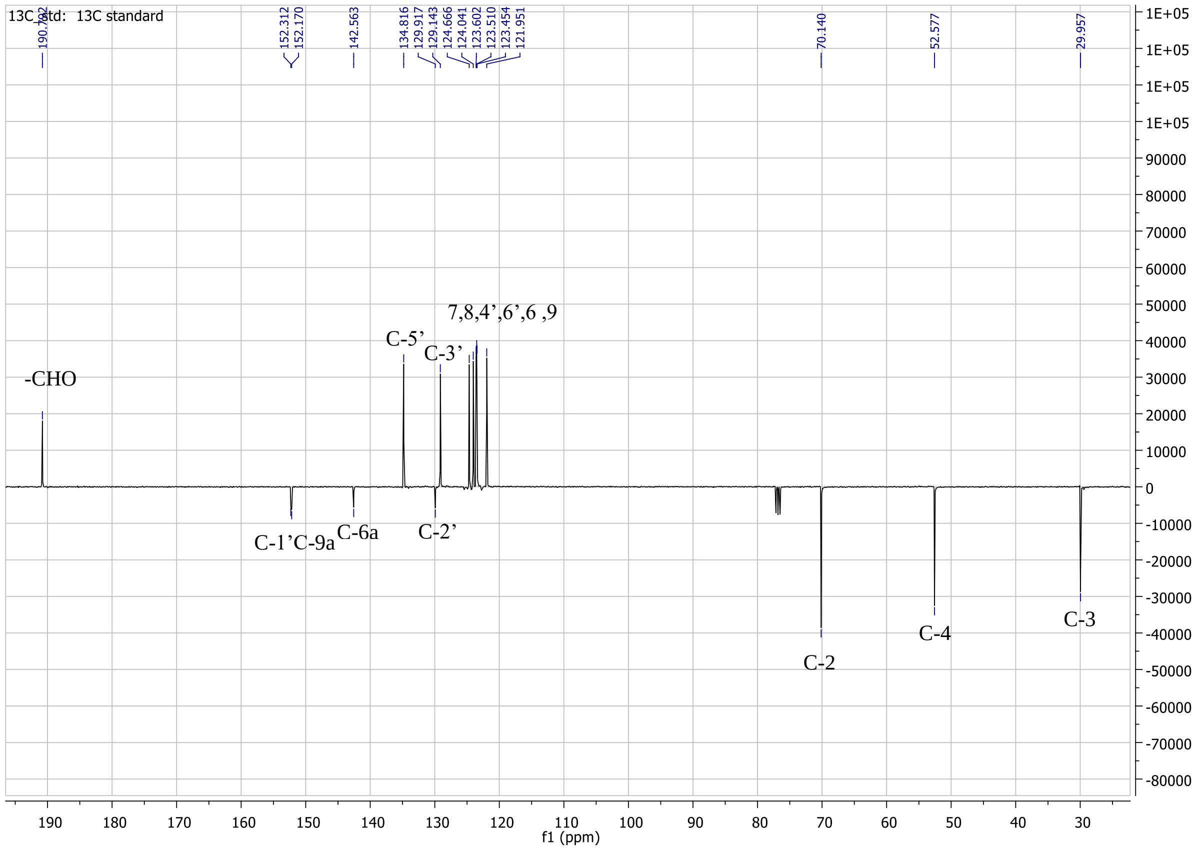

Figure S71. J-modulated ^{13}C-NMR spectrum of **4** measured in $CDCl_3$ (100 MHz)

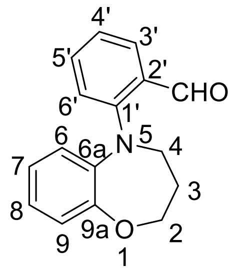

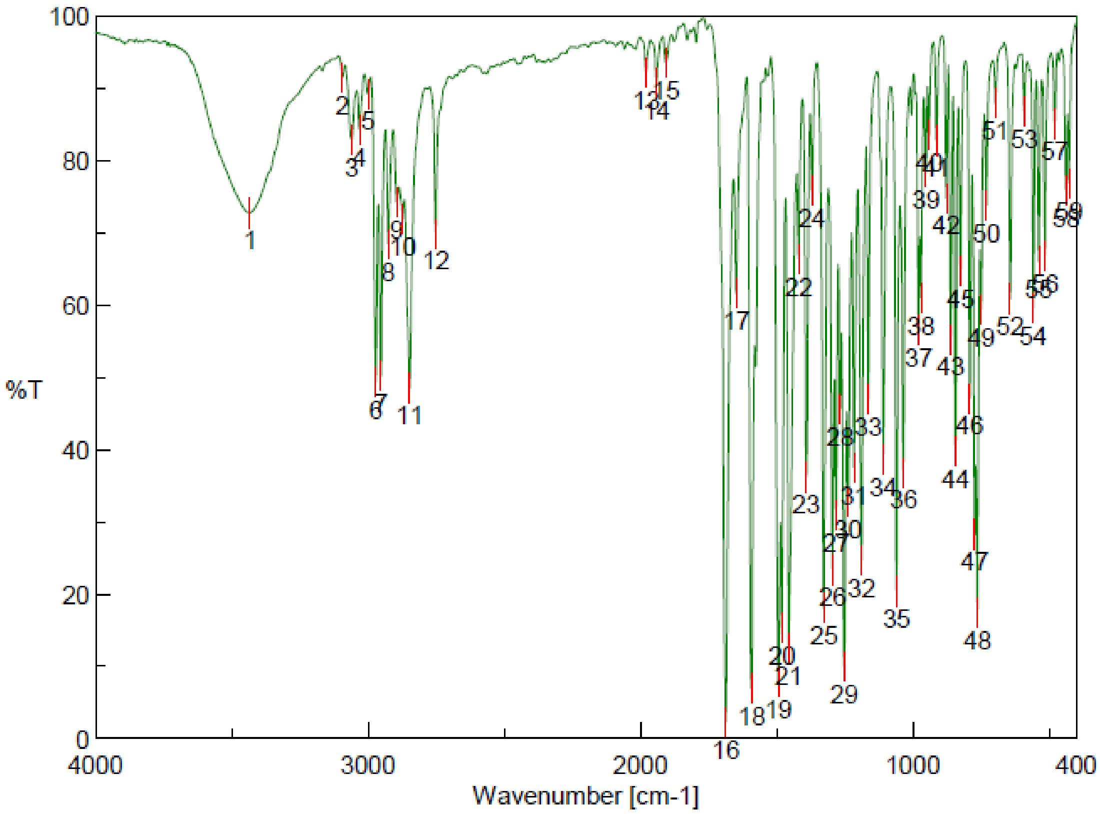

Figure S72. IR spectrum of **4** recorded as KBr disc

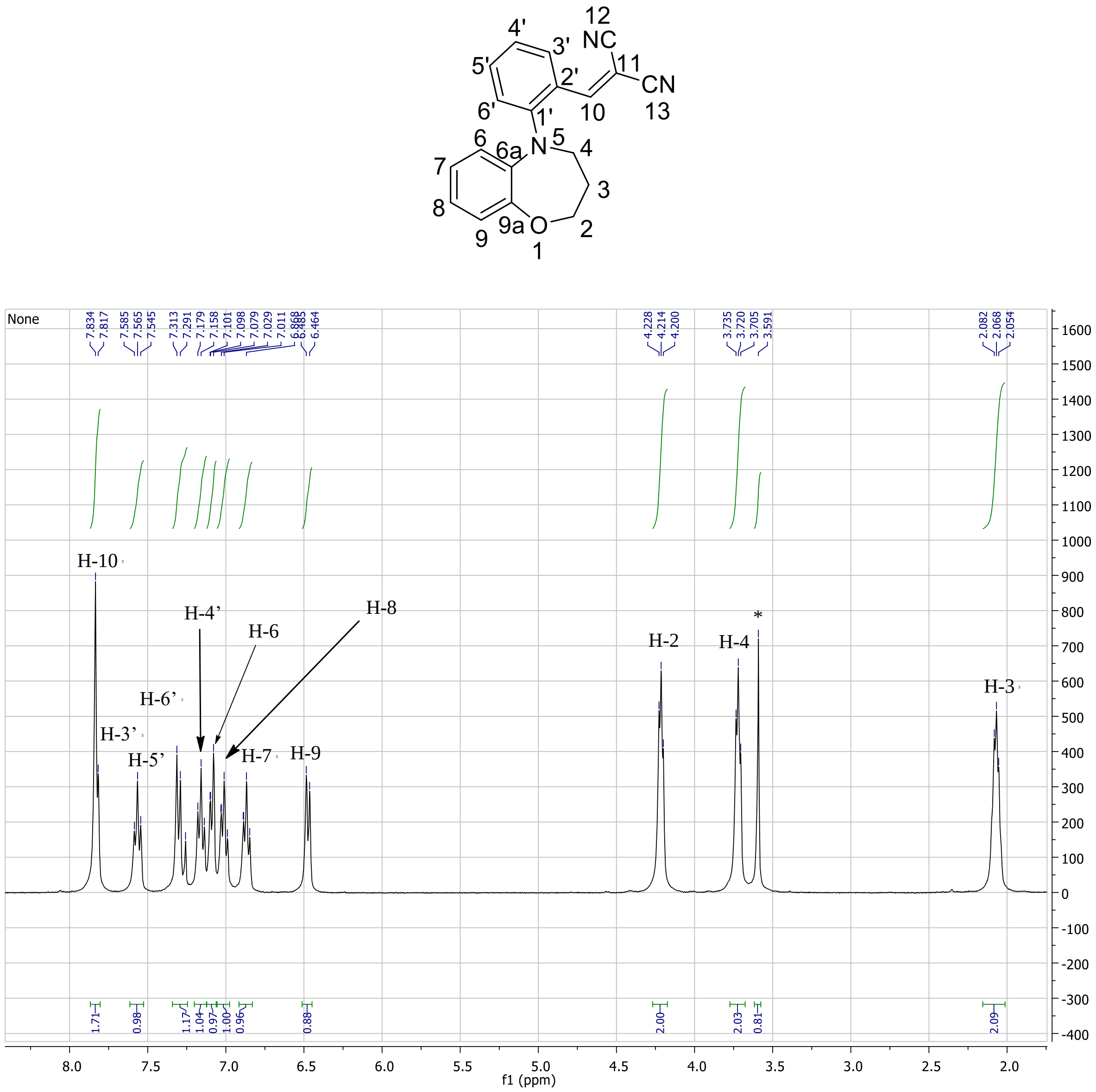

Figure S73. ^{1}H-NMR spectrum of **20** measured in $CDCl_3$ (360 MHz)

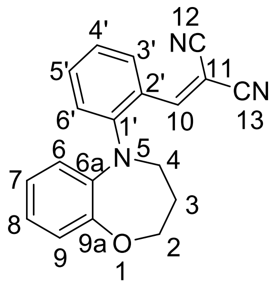

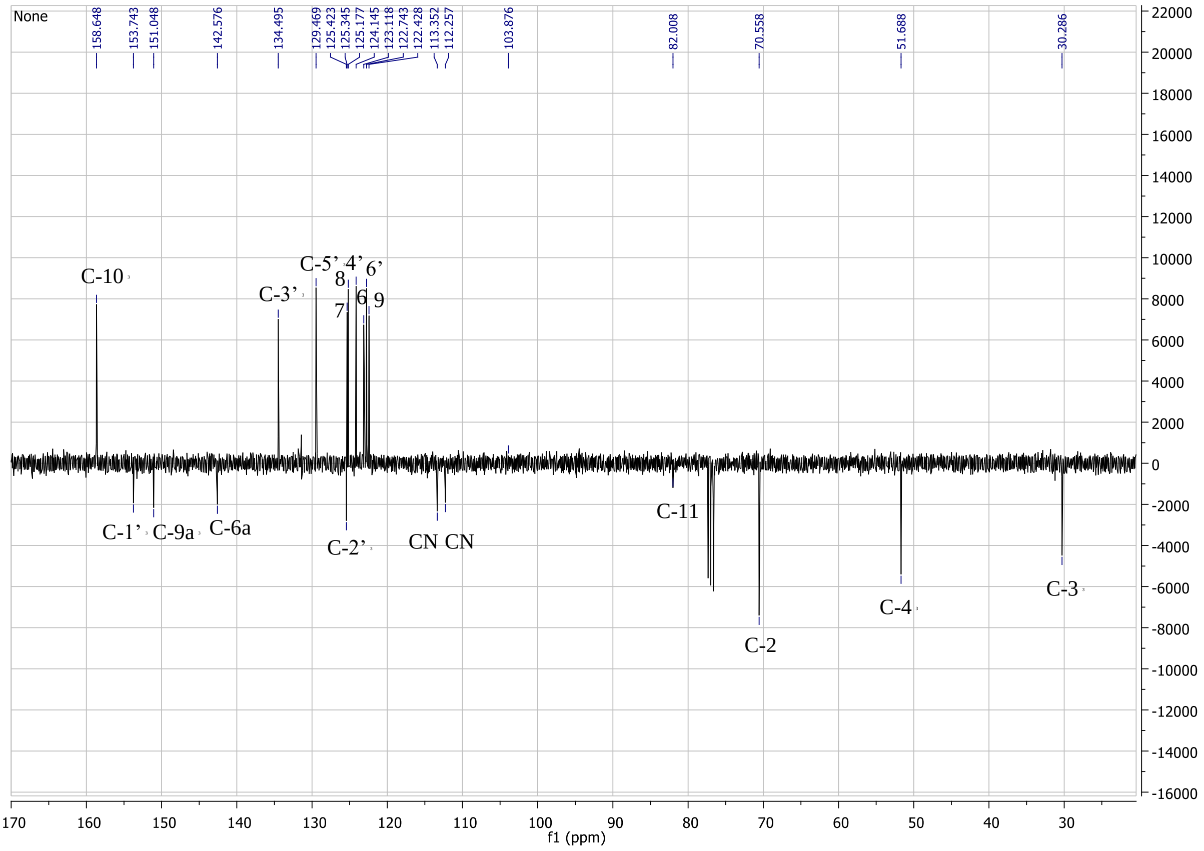

Figure S74. J-modulated ^{13}C-NMR spectrum of **20** measured in $CDCl_3$ (90 MHz)

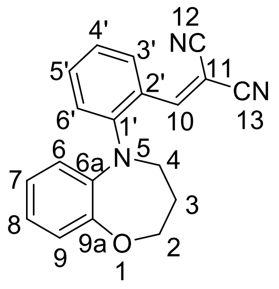

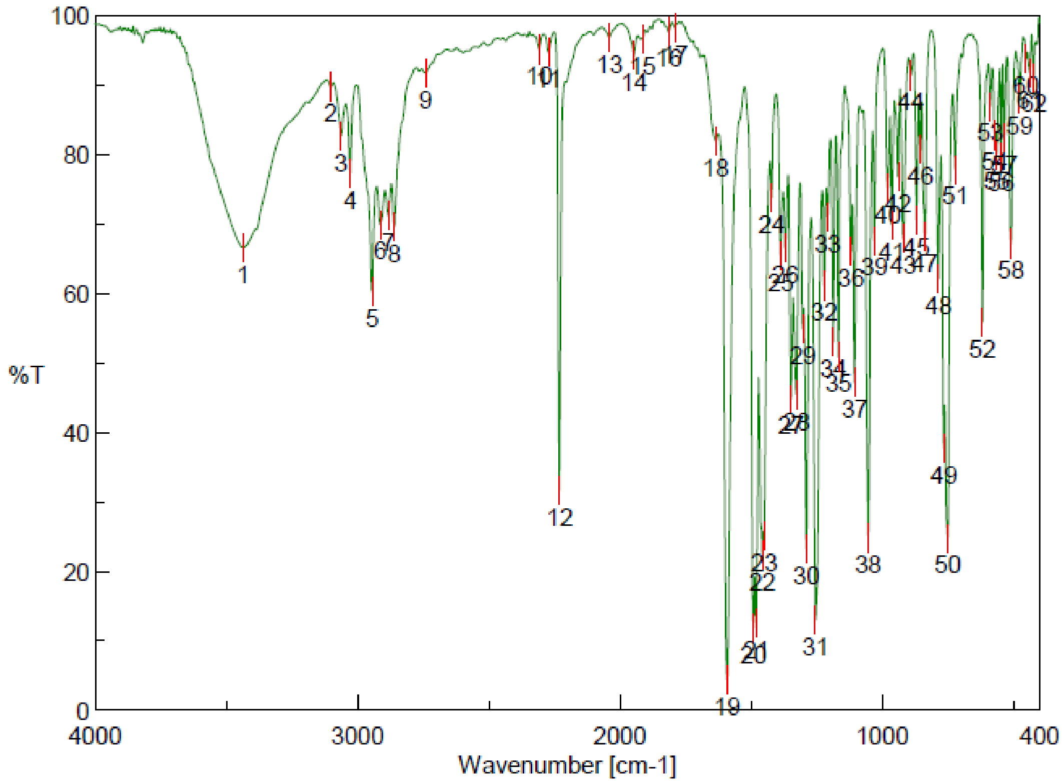

Figure S75. IR spectrum of **20** recorded as KBr disc

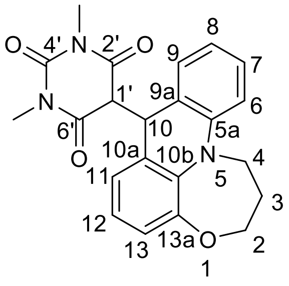

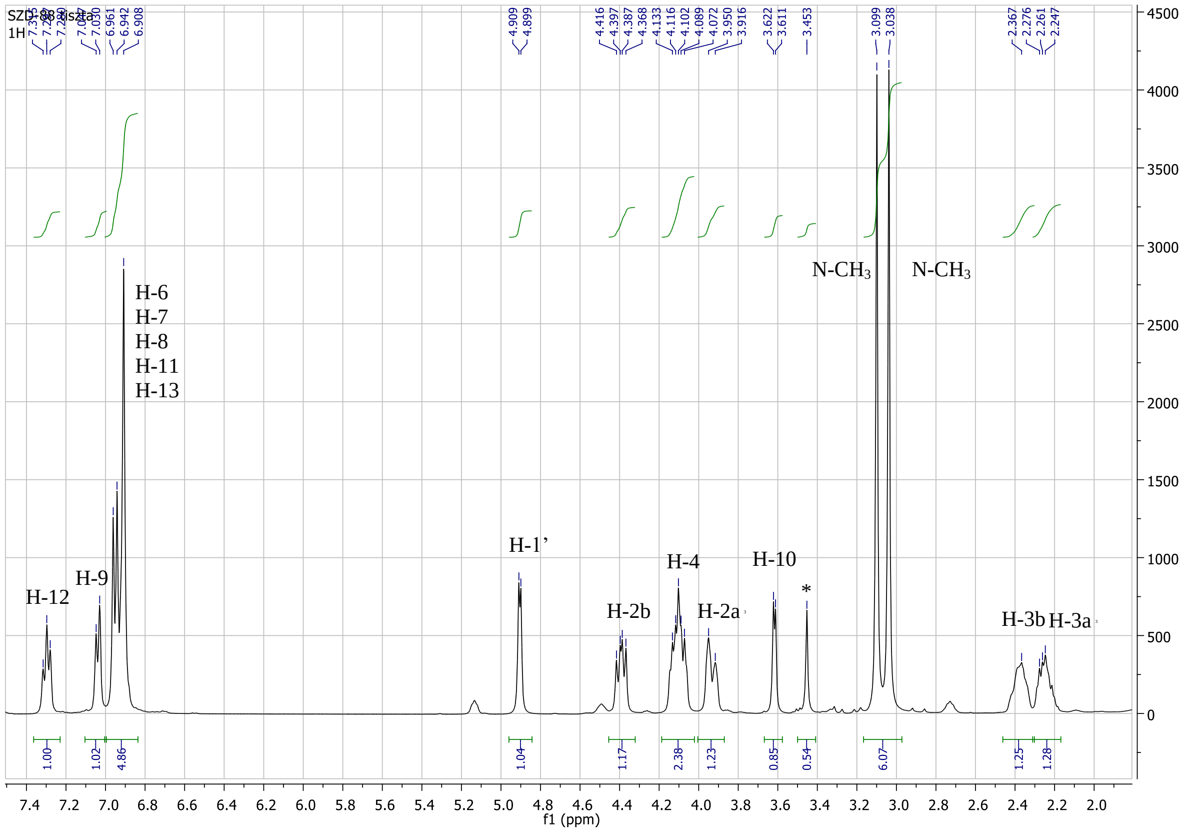

Figure S76. ^{1}H-NMR spectrum of **6** measured in $CDCl_3$ (400 MHz)

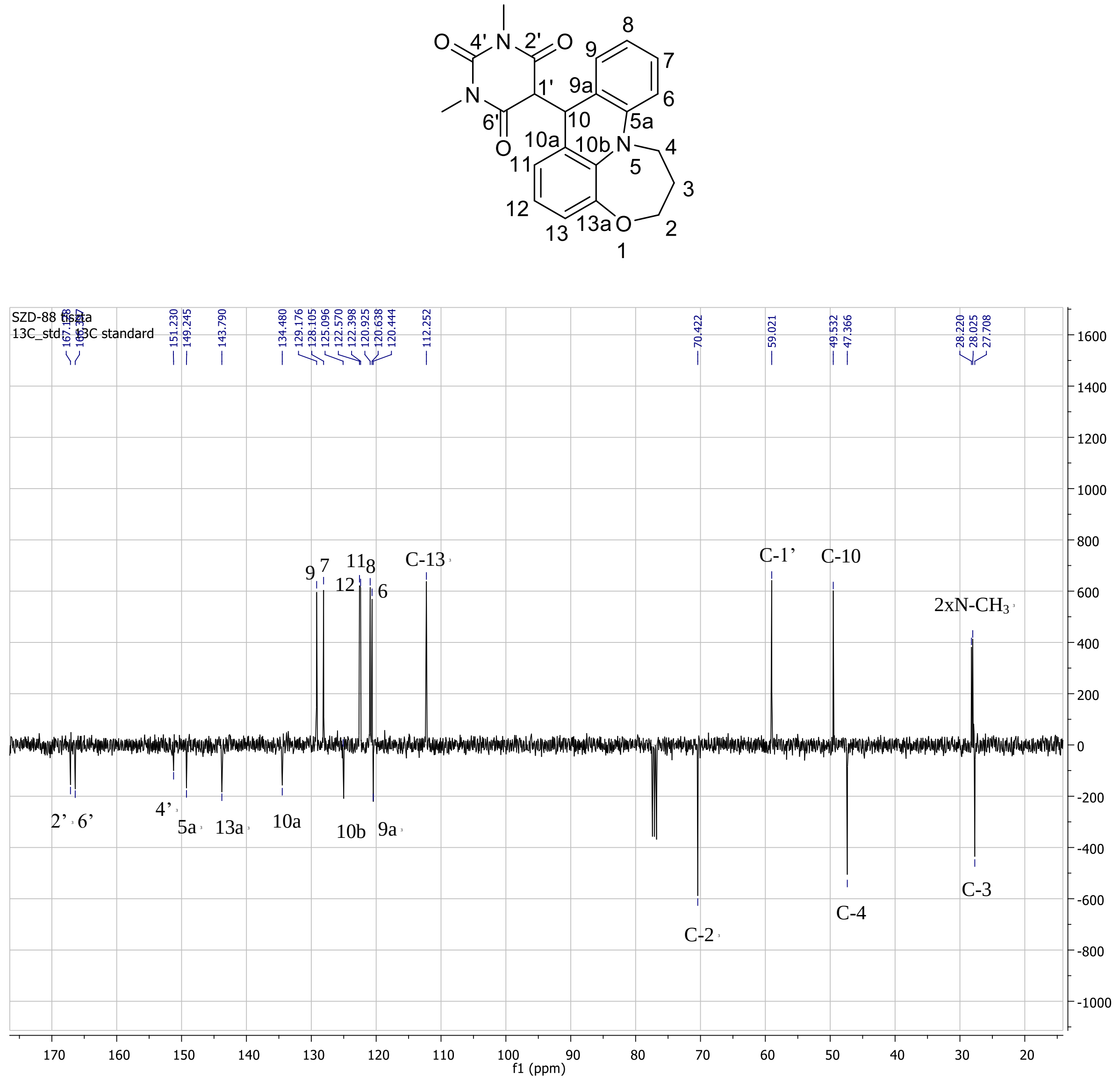

Figure S77. J-modulated ^{13}C-NMR spectrum of **6** measured in $CDCl_3$ (100 MHz)

organocatalyst	Reaction time (day)		Conversion (%)		ee (%)	
	A	B	A	B	A	B
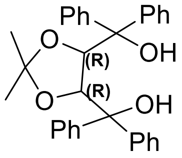	21	9	22	25	6.1	6.9
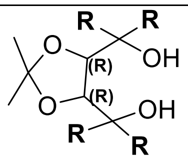 **R**: α-naftil	21	9	26	26	7.3	5.4
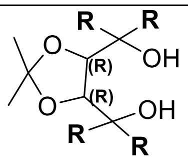 **R**: β-naftil	21	9	17	29	7.2	3.8
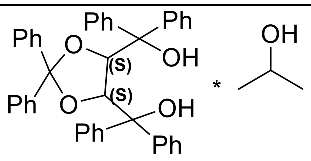	21	9	13	30	4.6	2.9
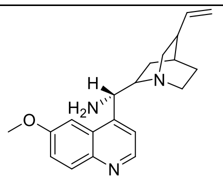	8	8	10	11	2.9	1.9
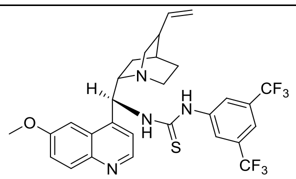	8	8	9	8	0.0	1.0

Table S1. Organocatalytic transformations of **1a** to *trans*-**10a** (columns A) and **1a** to **11a** (columns B); reaction condition: $MgSO_4/CHCl_3$, 0.3 equivalent of organocatalyst, rt.

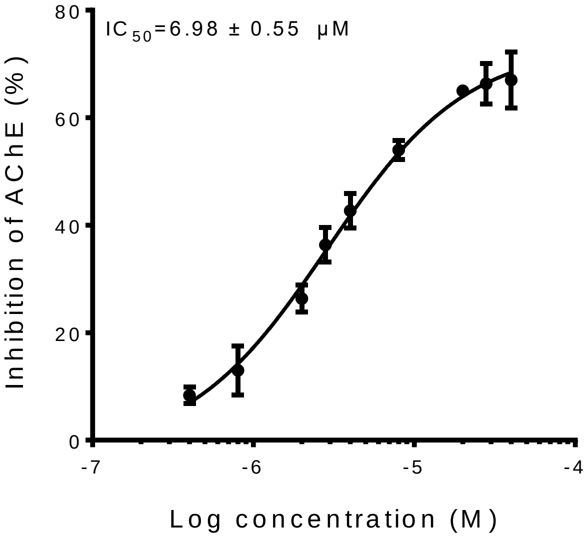

Figure S78. Concentration-dependent curve of **6** for the inhibition of AChE activity. IC_{50} of **6** was calculated to be 6.98 ± 0.55 μM. Every point is an average of three independent experiments (mean ± SD).
